# Murine typhus as the leading cause of non-focalized fever in the Canary Islands

**DOI:** 10.1007/s10096-024-04976-8

**Published:** 2024-11-29

**Authors:** M. Vélez-Tobarias, AM. Torres-Vega, E. Carmelo, J. Morais-Martín, JA. Pérez, C. Gonzalo-Hernández, G. Clot, C. Ascaso-Terrén

**Affiliations:** 1 Servicio de Medicina Interna, Hospital Universitario de La Palma, La Palma, Spain; 2 Medicina i Recerca Translacional, Facultat de Medicina i Ciències de La Salut, Universitat de Barcelona, Barcelona, Spain; 3 Servicio de Medicina Interna, Hospital Insular Ntra. Sra. de los Reyes (HINSR), Valverde, El Hierro Spain; 4 Instituto Universitario de Enfermedades Tropicales y Salud Pública de Canarias (IUETSPC), Universidad de La Laguna (ULL), Avenida Astrofísico Francisco Sánchez S/N, La Laguna, Tenerife Spain; 5 Departamento de Obstetricia y Ginecología, Pediatría, Medicina Preventiva y Salud Pública, Toxicología, Medicina Legal y Forense y Parasitología, Universidad de La Laguna, La Laguna, Spain; 6 Departamento de Bioquímica, Microbiología, Biología Celular y Genética, Facultad de Ciencias, Universidad de La Laguna, La Laguna, Spain; 7 Institut d’Investigacions Biomèdiques August Pi i Sunyer (IDIBAPS), Barcelona, Spain; 8 Department of Basic Clinical Practice, University of Barcelona, Barcelona, Spain

**Keywords:** Non-focalized fever, Murine typhus, Q fever, Molecular diagnosis, Serology, Incidence of zoonoses

## Abstract

**Purpose and methods:**

This prospective study aims to diagnose the etiology of non-focalized fever lasting between 5 and 28 days in the islands of La Palma and El Hierro (Canary Islands, Spain) during 2021, using serology and PCR.

**Results:**

The etiological profile described in this study aligns with that of fever of intermediate duration (FID), with zoonoses being the primary cause. Murine typhus (MT) is identified as the leading cause, followed by Q fever (QF). The incidence of MT is the highest reported nationally and comparable to the highest in Europe, with 39.6 cases per 100,000 inhabitants in La Palma and 79.7 cases per 100,000 inhabitants in El Hierro. Q fever, known to be endemic to the Canary Islands, presents incidences of 26.5 cases per 100,000 inhabitants in La Palma and 15.6 cases per 100,000 inhabitants in El Hierro. MT shows no gender differences and has a homogeneous geographical distribution. In contrast, QF is more prevalent in men and has a heterogeneous geographical distribution.

**Conclusions:**

The high incidence of MT found in both urban and peri-urban areas is particularly noteworthy. Its potential connection with climate change and/or the growth of the reservoir population in the Canary Islands remains unknown. MT's similarity to QF in terms of clinical signs and treatment, coupled with the absence of a specific protocol for early diagnosis, may have contributed to its underdiagnosis. MT can lead to significant health concerns, including risk of hospitalization, complications, and even death. Therefore, the registration of cases for epidemiological control is deemed essential.

**Supplementary information:**

The online version contains supplementary material available at 10.1007/s10096-024-04976-8.

## Introduction

Fever is the most frequent reason for medical consultation in both Primary Care and Emergency Department settings. The scientific literature offers various definitions of fever. For this study, non-focalized fever is defined as a temperature higher than 38 °C without signs or symptoms indicating its origin. Based on duration, we include short-duration fever (SDF), defined as fever lasting less than 7 days, and fever of intermediate duration (FID). The latter is a term used in Spain referring to fever lasting between 7 and 28 days without a history of hospital stay, immunodeficiency, or other chronic underlying disease that might explain its presence. After basic clinical and complementary evaluation, the fever remains undiagnosed. SDF frequently has an infectious origin, either viral or bacterial, and often follows an unremarkable clinical course. However, in some instances, the fever persists, and a diagnosis cannot be identified with initial diagnostic tests [1, 2].

Studies conducted in southern Spain have indicated a predominance of acute systemic infectious diseases as the main causes of FID, with zoonoses being the principal cause. Among these, the most frequent are Q Fever (QF), brucellosis (Br), Mediterranean spotted fever (MSF), and Murine Typhus (MT). Less frequent causes include typhoid fever, Epstein-Barr virus (EBV) mononucleosis, Cytomegalovirus (CMV), human immunodeficiency virus (HIV), toxoplasmosis (Tox), and leptospirosis (Lpt). Furthermore, there is a much lower percentage of causes associated with non-infectious pathologies and a variable percentage, depending on each study, of unknown cause (19–66%) [3, 4]. The etiological spectrum of FID varies according to multiple factors, including time and geographic location [5]. However, the number of national and international studies [1] on this subject remains scarce.

Publications have reported cases of QF [6–9] and, in recent years, MT [9–12] in the Canary Islands as sources of FID. Internationally, cases of MT have also been reported as a cause of FID in travelers returning from the Canary Islands [13, 14]. However, there is a lack of prospective and incidence studies on the main sources of FID in the archipelago [11, 12].

The aim of this study is to analyze in detail the etiologic spectrum of non-focalized fever lasting from 5 to 28 days on the islands of La Palma and El Hierro. Their insularity, combined with their small and manageable populations, make them ideal locations for this purpose. For methodological considerations, we encompassed a broader spectrum, starting at 5 days following the onset of fever and incorporating cases of FID. This approach was undertaken to identify and quantify the incidence of the most prevalent etiologies, determine their geographical distribution, and identify populations at risk.

## Method

This prospective study focused on diagnosed cases of non-focalized fever lasting between 5 and 28 days on the islands of La Palma and El Hierro during 2021.

The study was conducted amidst the SARS-CoV-2 pandemic, characterized by a high clinical suspicion of coronavirus infection among cases presenting with fever. Patients typically sought clinical consultation at early stages, often within the first 24 h of fever onset. During the case recruitment stage, we initially incorporated the defining criteria of FID. However, given the substantial number of patients with prolonged non-focalized fever within 7 days of onset, we decided to include patients experiencing fever persisting for a duration of 5 days or more.

Study Setting: The Spanish islands of El Hierro and La Palma are located in the Canary Archipelago, one of the outermost regions of the European Union. Situated in the Atlantic Ocean, northwest of Africa and close to the southern coast of Morocco, these islands enjoy a subtropical climate.

The Canary Islands Health Service is organized geographically into seven Healthcare Divisions, one for each island. Each Division has at least one General Hospital, providing services tailored to the specific health needs and demographic characteristics of the local population.

La Palma, the second westernmost island of the archipelago, has a surface area of 708.33 km^2^. Its Healthcare Division is organized into 7 Basic Health Areas and one General Hospital, the University Hospital of La Palma.

El Hierro, the smallest and westernmost island, has a surface area of 268.71 km^2^. Its Healthcare Division is organized into two Basic Health Areas and one hospital, the Nuestra Señora de los Reyes Hospital.

Study Population: The study population comprised the entire population of La Palma and El Hierro, corresponding to 83,380 and 11,298 inhabitants respectively, as of January 1, 2021, according to data from the Canary Islands Statistics Institute (Instituto Canario de Estadística, ISTAC) [15].

Recruitment and Sample Collection: We established a sentinel primary care physician network in each Basic Health Area of La Palma and El Hierro for patient recruitment and sample submission. The Internal Medicine Services, Emergency Departments, and COVID-19 units were also included in this network. The sentinel physicians served as study referrers in their respective areas. The inclusion criteria are defined in Table 1.

**Table 1.** Inclusion Criteria

>38ºC fever, without obvious source of infection from 5 to 28 days of duration.
1) No hospital admission in the last 2 weeks.
2) No HIV with CD4<500 or other known immunodeficiencies.
3) Non-diagnosis despite basic preliminary studies, including clinical history, physical examination, complete blood count, urine sediment, basic biochemistry with liver function test, kidney function test, PCR, and chest X-ray.
4) Immigrants with more than 6 months residency.
6) Patients who are not on dialysis or with intravascular catheters.
7) Non-active drug users
8) Not having travelled outside Spain in the last 3 months.
9) Only patients above 14 years of age are included.

In the Health Service System, a standardized protocol was implemented to rule out SARS-CoV-2 infection in individuals presenting with fever. Within the initial 24 h of fever onset, patients underwent a SARS-CoV-2 PCR test, with a follow-up test scheduled 72 h thereafter if the initial result was negative. Patients without evidence of SARS-CoV-2 infection who continued to exhibit fever symptoms were referred to the sentinel network of physicians for further evaluation including anamnesis, physical examination, complete blood count, biochemistry profile, urine sediment and chest X-ray. Those patients with persisting fever beyond 5 days despite negative findings were enrolled in the study. Patients diagnosed with SARS-CoV-2 infection, focal fever, or those who became afebrile prior to the 5-day mark were excluded from the study. Treatment decisions for patients not meeting the study criteria were determined based on clinical judgment by the attending physician.

Upon inclusion in the study, patients were required to sign an informed consent form. A 2 ml blood sample was drawn into an Ethylenediaminetetraacetic acid (EDTA) tube and frozen at -80 °C for molecular detection [16] of: *Coxiella burnetii*, *Rickettsia* spp., *Bartonella* spp., and *Anaplasma/Ehrlichia* spp. at the Institute of Tropical Diseases and Public Health of the Canary Islands (IUETSPC).

Serological studies were conducted regularly by the Canary Islands Health Service. Initially, serologies for *Coxiella burnetii*, *Rickettsia typhi*, Cytomegalovirus, and Epstein-Barr virus were requested. When no diagnosis was found, serial serology for rickettsiosis or QF was performed between 14 and 30 days after the first serology. In cases with negative results, the serological study was extended to include *Chlamydophila pneumoniae*, *Mycoplasma pneumoniae*, HIV, Parvovirus B19, *Treponema pallidum, Borrelia burgdorferi*, and *Brucella melitensis*. The serological techniques and cutoff points used are detailed in Table 2.

**Table 2:** Serological techniques used. IIF: Indirect immunofluorescence, **ELISA:** enzyme-linked immunosorbent assay, **CL:** chemiluminescence

Microorganism	Technical Serology	Cutoff points
*Coxiella burnetii*	IIF (Vircell Laboratories)	IgM ≥ 1/64 and/or IgG ≥ 1/1024 or quadrupling of IgG titers between 2 consecutive samples
*Rickettsia typhi*	IIF (Pasteur Laboratories)	IgM ≥ 1/192 or quadrupling of IgG titers in 2 consecutive samples
*Rickettsia conorii*	IIF (Pasteur Laboratories)	IgM ≥ 1/192 or quadrupling of IgG titers in 2 consecutive samples
EBV	ELISA CL (Abbott Laboratories)	Qualitative
CMV	ELISA CL (Abbott Laboratories)	Qualitative
*Chlamydophila pneumoniae*	IIF (Vircell Laboratories)	IgM ≥ 1/64 y/o IgG ≥ 1/512
*Mycoplasma pneumoniae*	ELISA CL (Vircell Laboratories)	Qualitative
*Parvovirus B19*	ELISA CL (Vircell Laboratories)	Qualitative
*Treponema pallidum*	ELISA CL (Abbott Laboratories)	Qualitative
*Borrelia burgdorferi*	ELISA CL (Vircell Laboratories)	Qualitative
HIV	ELISA CL and Western- Blot (Abott Laboratories)	Qualitative
*Brucella melitensis*	Rosa Bengala agglutination test (Linear)	Qualitative

All patients who met the inclusion criteria and had clinical suspicion of rickettsiosis or QF were administered empirical antimicrobial therapy with doxycycline after blood sampling. All cases diagnosed by serology and/or PCR test were considered positive.

Statistical Analysis: Quantitative variables were described by calculating their means, standard deviations, maximum and minimum values. Qualitative variables were represented with frequency tables. Subpopulations were compared using Chi-squared test, Student's t-distribution, and logistic regression models. Statistical analyses were performed with the R 4.2.2 package. Hypothesis tests were evaluated with an alpha risk of 5%, and confidence intervals (CI) were computed at the 95% level.

## Results

During 2021, we registered 79 patients: 65 on La Palma and 14 on El Hierro. Among the selected cases, 48 (60.7%) were from the Emergency Department, 21 (26.6%) from Internal Medicine hospitalization, and 3 (3.8%) from the Intensive Care Unit (ICU). The remaining 7 cases (8.9%) came from Primary Care. A significant majority (88.6%) of the cases were registered within 10 days of fever onset, with 45.6% occurring between 5 and 6 days.

Of the 79 cases, we conducted PCR combined with amplicon sequencing on 71 patients and serology on 73 individuals. Serological analysis revealed: 23 positive results of *Rickettsia typhi*, 13 of *Coxiella burnetii*, 8 of *Mycoplasma pneumoniae*, 5 of cytomegalovirus (CMV), 3 positive results each of Epstein-Barr virus (EBV) and Parvovirus B19, 2 of *Chlamydia pneumoniae *and 1 of *Rickettsia conorii.*

Molecular diagnosis identified 38 positive results of *Rickettsia typhi*, 10 of *Coxiella burnetii* and 1 case of *Bartonella henselae*.

No detections of *Anaplasma/Ehrlichia* spp. were found. Notably, no *Rickettsia* species other than *Rickettsia typhi* were identified. Nineteen patients tested positive for *Rickettsia typhi* and 7 patients for *Coxiella burnetii* by both PCR and serology. Blood cultures from one patient yielded *Enterococcus faecalis*. Thirteen patients exhibited negative results on both PCR and serological testing, with one patient clinically diagnosed with Central Fever. Table 3 provides a detailed description of patient diagnosis taking into account possible serological cross-reactions.

**Table 3:** Distribution of diagnosed cases, ICU admissions, and deaths for each pathology diagnosed

Positive results	No. of cases	Hospitalization	ICU	Deaths	Possible Cross-reaction	Diagnosis
*Rickettsia typhi*	34	18	2	2	No	MT
*Coxiella burnetii*	10	4			No	QF
*Rickettsia typhi & Coxiella burnetii*	2				No	TM/QF
*Rickettsia typhi* + *Parvovirus B19* + *Mycoplasma pneumoniae*	1				Yes	MT
*Rickettsia typhi* + *Cytomegalovirus* + *Mycoplasma pneumoniae*	1				Yes	MT
*Rickettsia typhi* + *Mycoplasma pneumoniae*	2				Yes	MT
*Rickettsia typhi* + *Cytomegalovirus*	1				Yes	MT
*Rickettsia conorii* & *Rickettsia typhi*	1				Yes	MT
*Coxiella burnetii* + *Cytomegalovirus* + *Parvovirus B19* + *Mycoplasma pneumoniae*	1				Yes	MT
*Coxiella burnetii* + *Chlamydia pneumoniae*	2				Yes	QF
*Coxiella burnetti* + *Parvovirus B19*	1				Yes	QF
*Cytomegalovirus* + *Epstein Barr Virus* + *Mycoplasma pneumoniae*	1				Yes	CMV
*Cytomegalovirus* + *Epstein Barr Virus*	2				Yes	EBV
*Mycoplasma pneumoniae*	2				No	Myc
*Bartonella henselae*	1	1			No	Bar
*Enterococcus* bacteremia	1	1				Bacteriemia
Central Fever	1	1		1		Central Fever
TBC	1	1				TBC
Unknown Origin	14	5	1			-
Total	**79**	**31**	**3**	**3**		

MT: Murine typhus; QF: Q fever; CMV: Citomegalovirus; EBV: Epstein Barr Virus; Myc: Mycoplasmosis; Bar: Bartonellosis; TBC: tuberculosis.

The most common causes of non-focalized fever were Murine Typhus (MT) and Q Fever (QF), accounting for 73.4% of all cases. The diagnosed cases of TM (42 cases) and QF (16 cases) are shown in Table 4 and 5, respectively. Up to 17.7% of fever cases remained of unknown cause.

**Table 4:** Positive Tiphus Murine cases

Case	Age	Gender	Fever onset days	Max. Tª (ºC)	Positive results RT	SER RT IgM/IgG	SC	Other positive SER	Possible Cross-reaction
1	55	F	8	38.5	SER/PCR	1536/40	Yes	No	No
2	34	M	5	38.5	SER/PCR	384/160	No	No	No
3	37	F	8	39	SER/PCR	768/160	Yes	No	No
4	15	M	5	39	SER/PCR	192/40	-	No	No
5	72	M	8	38.8	SER/PCR	3072/160	Yes	No	No
6	21	M	6	40	SER/PCR	384/40	Yes	No	No
7	56	M	10	39	SER/PCR	384/80	No	VEB	Yes
8	28	M	5	40.2	SER/PCR	1536/320	Yes	No	No
9	24	F	6	39.4	SER/PCR	1536/80	Yes	Myc	Yes
10	61	M	11	39.5	SER/PCR	160/640	-	No	No
11	60	F	9	39	SER/PCR	640/384	Yes	No	No
12	18	F	8	40	SER/PCR	3072/160	Yes	No	No
13	56	M	5	39	SER/PCR	768/0	-	No	No
14	56	M	7	39.8	SER/PCR	1536/40	Yes	B19/Myc	Yes
15	40	M	5	39	SER/PCR	768/80	Yes	No	No
16	60	F	7	38.5	SER/PCR	3072/80	Yes	No	No
17	18	M	7	39.5	SER/PCR	1536/160	Yes	No	No
18	66	M	7	38.7	SER/PCR	1536/80	Yes	No	No
19	72	M	7	39	SER/PCR	1536/80	Yes	No	No
20	22	M	12	39.9	SER	3072/320	Yes	No	No
21	27	F	40	19	SER	384/0	Yes	No	No
22	83	M	38	5	SER	384/640	Yes	No	No
23 (D)	87	F	38	5	SER	1536/160	Yes	No	No
24	69	M	10	39	PCR			No	No
25	25	M	6	40	PCR			No	No
26	37	F	6	38.8	PCR			Myc	Yes
27	49	F	8	38.5	PCR			No	No
28	31	F	7	40	PCR			CMV	Yes
29	40	M	7	42	PCR			No	No
30	42	M	7	38	PCR			No	No
31	74	F	9	39	PCR			No	No
32	53	M	6	39	PCR			No	No
33	30	M	7	39	PCR			EBV	Yes
34	52	M	8	39	PCR			No	No
35	58	F	10	38	PCR			Myc/CMV	Yes
36	54	M	13	39	PCR			No	No
37 (D)	84	F	5	38.5	PCR			No	No
38	43	M	5	39	PCR			No	No
39	21	F	7	40	PCR			No	No
40	60	F	7	39	PCR			No	No
41	18	M	6	39	PCR			No	No
42	14	M	40	7	PCR			No	No

(D): Death cases; Max. Tª: máximum temperatura; SC: seroconversión; SER: serology; SER RT IgM/IgG: *Rickettsia typhi* serology titre** (**1:IgM/1:IgG**)** RT: *Rickettsia typhi*; CMV: *Citomegalovirus*; EBV: *Epstein Barr Virus*; B19: *Parvovirus B19*; Myc: *Mycoplasma pneumoniae*

**Table 5:** Positive Q fever cases

Case	Age	Gender	Fever onset days	Max. Tª (ºC)	Positive results CB	SER CB IgM/IgG	SC	Other positive SER	Possible Cross-reaction
1	56	M	7	38,7	PCR			–	-
2	15	M	9	40	PCR			Chla	Yes
3	70	M	10	40	PCR			No	No
4	58	F	7	39.6	SER	0/2048	Yes	Chla	Yes
5	58	F	8	39.9	SER	0/4096	Yes	No	No
6	81	M	17	39	SER	0/32768	–	No	No
7	68	F	6	38.5	SER	128/8192	Yes	B19	Yes
8	47	M	6	39.5	SER	512/128	Yes	No	No
12	54	M	13	39	SER	2048/256	–	No	No
9	45	M	5	40	SER/PCR	0/1024	Yes	No	No
10	81	M	5	39.5	SER/PCR	512/2048	Yes	No	No
11	46	F	5	38	SER/PCR	128/16384	–	No	No
13	71	M	5	39	SER/PCR	8192/1024	–	No	No
14	38	M	5	39.5	SER/PCR	1024/512	Yes	No	No
15	24	M	5	38.5	SER/PCR	512/2048	Yes	Myc/B19/CMV	Yes
16	52	M	8	39	SER/PCR	256/2048	Yes	No	No

Max. Tª: máximum temperatura; SC: seroconversión; SER: serology; SER CB IgM/IgG: *Coxiella burnetii* serology titre (1:IgM/1:IgG); CB: *Coxiella burnetii*; Chla: *Chlamydia pnemoniae*; B19: *Parvovirus B19*; Myc: *Mycoplasma pneumoniae*; CMV: *Cytomegalovirus*

The incidence of MT was 39.6 cases per 100,000 inhabitants/year in La Palma (95% CI, 25–54) and 79.7 cases per 100,000 inhabitants/year in El Hierro (95% CI, 23–136). QF was the second most common cause with 26.5 cases per 100,000 inhabitants/year in La Palma (95% CI, 5–78) and 15.6 cases per 100,000 inhabitants/year in El Hierro (95% CI, 7–25). For both etiologies, El Hierro had the highest occurrences, with approximately twice the incidence of La Palma **(**Table 6**).**

**Table 6:** Incidences by pathology on the islands of La Palma and El Hierro. Confidence interval (CI) at 95% level

	**LA PALMA**		**EL HIERRO**	
**Diagnoses**	**N**	**Incidence per 100000** **CI (95%)**	**N**	**Incidence per 100000** **CI (95%)**
Q fever	13	15.59 (7–25)	3	26.55 (5–78)
Murine Typhus	33	39.58 (25–54)	9	79.66 (23–136)
Cytomegalovirus Mononucleosis	1	1.20 (0–7)	0	8.85 (0–49)
Epstein Barr Virus Mononucleosis	2	2.40 (0–9)	0	0
Mycoplasmosis	2	2.40 (0–9)	0	0
Bartonellosis	0		1	8.85 (0–49)
Bacteriemia	1	1.20 (0–7)	0	0
Unknown origin	13	15.59 (7–25)	1	8.85 (0–49)
Non-infectious cause	1	1.20 (0–7)	0	0

Of the 79 cases, 51 were males (64.6%) and 28 females (35.4%). There were no significant differences in age between men and women (*p* = 0.39), with a mean age of 49.6 years (range: 14–90). No age differences were found between men and women diagnosed with MT (p = 0.45; mean age 45.7 years, 95% CI 39.2–52.2) or QF (*p* = 0.67; mean age 54 years, 95% CI 44.2–63.8). While no gender differences were found for MT (*p* = 0.12), significant differences were observed for QF (*p* = 0.05), with three times more men affected than women.

**Fig. 1:**
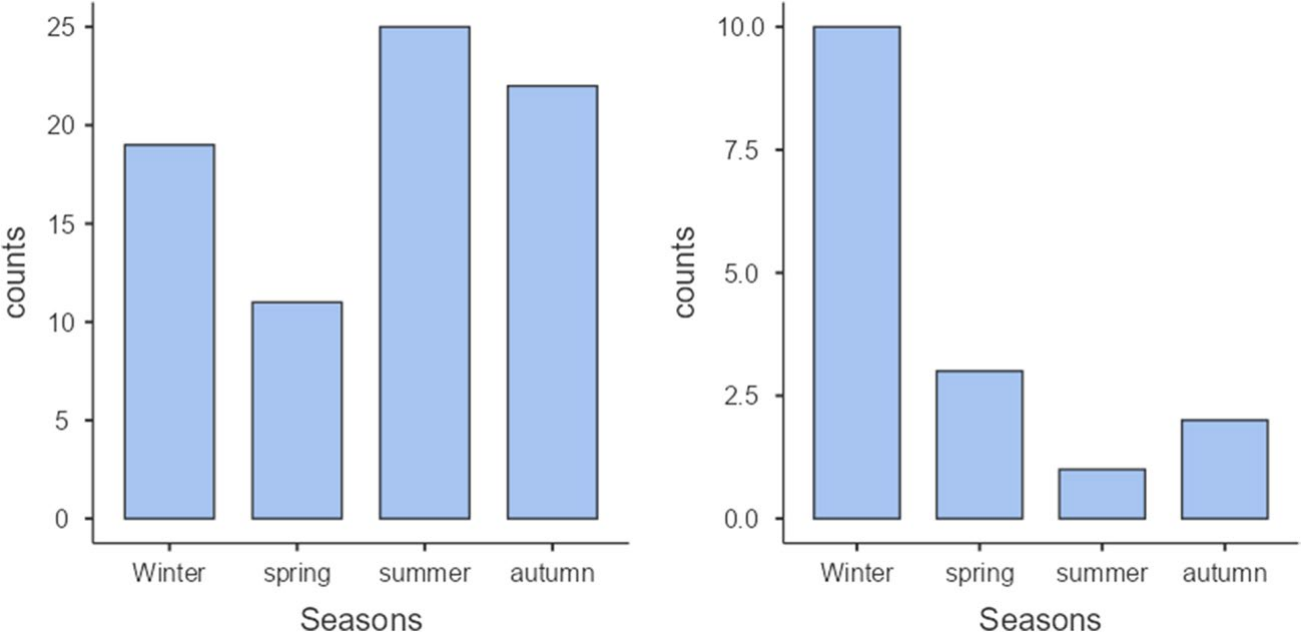
Distribution of the number of cases of MT (left) and QF (right) by season

Regarding seasonality, no statistically significant differences were observed in the overall distribution of cases per month (*p* = 0.10), likely due to insufficient statistical power. However, MT showed peaks during summer and fall months (*p* = 0.06), while QF demonstrated clear seasonality with a peak during winter months (*p* = 0.01) (Fig. 1).

MT cases were registered in all Basic Health Areas, with average incidences of 30–50 cases per 100,000 inhabitants/year in urban and peri-urban areas, and 50–120 cases in rural areas. For QF, incidences typically ranged between 20 and 25 cases per 100,000 inhabitants/year.

Of the total cases, 31 (39.2%) required hospitalization, with the majority (58.1%) associated with MT. Complications developed in 33% of cases: 42.5% were respiratory (pneumonia, interstitial infiltrates on chest imaging or pleural effusion), 24% involved alterations in renal function, 1% cardiac decompensation and other 1% neurological involvement.

Among MT cases, 42.8% were hospitalized, with 2 patients requiring ICU admission. Eleven cases (26%) presented one or more complications: 19% (8) respiratory, 9.5% (4) renal failure, 2.4% (1) heart failure, and 2.4% (1) neurologic involvement. Two patients with MT died, both over 80 years of age, one from the ICU and the other from Internal Medicine.

Regarding QF patients, 25% were hospitalized, and only one case presented complications with an infrarenal aortic aneurysm.

## Discussion

The implementation of a specific protocol via a network of sentinel physicians facilitated the early detection of non-focalized fever cases. Ninety percent of cases were identified within 10 days, with 46% diagnosed within 5–6 days of fever onset. Early blood collection, conducted prior to any therapeutic intervention, enabled the acquisition of optimal samples for molecular diagnosis [17], resulting in a 67.7% positivity rate among the total PCR tests conducted.

This prospective study is the first published in the Canary Islands to describe the etiological profile of non-focalized fever. Despite including cases from 5 days of fever onset, all identified diagnoses align with the FID profile. Zoonoses emerge as the primary cause of fever of unknown origin, with Murine Typhus (MT) as the predominant etiology, followed by Q Fever (QF). Previous publications have not reported such a high prevalence of MT, despite its consideration as an emerging cause of FID [11, 12]. These disparities may stem from the prospective nature of this study, incorporating molecular diagnosis [16] and earlier case recruitment.

Notably, despite the significant number of rickettsiosis cases in our series, *Rickettsia felis* was not detected. Although human cases of *R. felis* have been documented in the Canary Islands via serology [18], its absence in our series could be attributed to stringent clinical recruitment criteria, primarily focused on high and persistent fever. Consequently, these findings lend support to the hypothesis that the potential pathology of *R. felis* in humans may be less aggressive than that of *R. typhi*, albeit this remains an unresolved question [19].

No cases of anaplasmosis, brucellosis, or Mediterranean spotted fever were reported, although some positive results have been described in studies conducted in mainland Spain [3, 4, 20]. Additionally, although one positive serological result for *R. conorii* were found, they were considered to be false positive due to cross-reaction between *Rickettsia* species, as described in other publications [12]. Furthermore, several positive serological results of mycoplasmosis associated with other infections were identified. However, as the diagnostic approach for mycoplasmosis has been solely serological, the possibility of serological cross-reactivity should be contemplated in future investigations to elucidate its role as an etiological factor in FID or non-focalized fever.

Our findings indicate a higher incidence of non-focalized fever in males than in females, although there were no differences in terms of age, with a mean age of 49.6 years. Upon analysis by pathology, we observed that this gender difference was attributed to QF (p < 0.05), with three times more cases in males than in females. Consistent with other studied series [7, 9, 21], QF was also more prevalent in males and is associated with occupations related to livestock farming. No gender disparities were identified in cases of MT, consistent with findings from other series [9, 11].

The highest nationally reported incidence of MT is documented at 39.6 cases per 100,000 inhabitants/year in La Palma and notably at 79.7 cases per 100,000 inhabitants/year on El Hierro. According to the study by Beatriz Rodríguez-Alonso et al., the Canary Islands exhibit the highest national incidence of MT, with a rate of 1.2 cases per 100,000 inhabitants/year, significantly lower than our findings [12]. Such variations may originate from differences in sample study areas, sampling timeframes, the prospective design employed in this study, and the early diagnosis due to the implementation of molecular diagnostics. Thus, while the observed incidences are remarkably high, they may accurately reflect the realities of our geographic context.

Internationally, our reported incidences may be comparable to those of Croatia [22], with 57 cases per 100,000 inhabitants/year, and Greece [23] with 87 cases per 100,000 inhabitants/year, as published by Punda-Polic et al. and George Chaliotis et al., respectively.

Q Fever, the second most prevalent cause of non-focalized fever in our study, showed incidences of 26.5 cases per 100,000 inhabitants/year on La Palma and 15.6 cases per 100,000 inhabitants/year on El Hierro. These rates exceed those estimated in Gran Canaria (2003) and are comparable to those observed in Lanzarote (1997) with 5 and 12 cases per 100,000 inhabitants/year, respectively [7, 24]. However, they are lower than those reported by Vélez-Tobarias in 2013 in La Palma (43 cases per 100,000 inhabitants/year) in a thesis for the master's degree in International Health and Tropical Medicine at the University of Barcelona. Recent literature on the incidence of QF in the Canary Islands is scarce. Based on the available data, it could be interpreted that the incidence of QF is declining in the Canary Islands, possibly attributable to enhanced sanitary measures concerning the cattle population. Internationally, there are documented case series detailing QF outbreaks and some incidence studies reporting lower rates than those observed in this study. Raoult et al. described a rate of 2.5 cases per 100,000 inhabitants/year in France [25], with even lower rates in Switzerland.

The geographical distribution of MT is notably high, with a remarkable absence of cases in the north of La Palma, where temperatures are lower.Regarding QF, there is a spatial distribution by focus of infection, which could be associated with the animal health management of some goat herds. The highest incidence, about 100 cases per 100,000 inhabitants/year, was found in the south of El Hierro.

QF displays a pronounced seasonal trend, particularly notable during the winter months. This pattern is subject to variation depending on the specific geographical location under examination [21, 25]. It is particularly associated with the annual goat kidding cycle that farmers establish for the sale of kids during Christmas. Conversely, in other islands, calving occurs biannually, resulting in concentrated cases during two distinct seasonal spikes [6, 7, 9]. In contrast, cases of MT demonstrate a seasonal increase during the summer and fall months, aligning with the period of peak reproductive activity of its vector, the flea [9, 11]. This observation may account for the diminished presence of MT in colder regions and seasons. These biological cycles are in line with those described in previous studies [19].

Nearly 43% of MT cases were identified during hospitalization, with 26% experiencing medical complications, 4.7% requiring admission to the ICU, and almost 5% resulting in fatalities. These findings are consistent with the existing literature [12, 26]. Regarding QF, 25% of the cases were also identified during hospitalization, with 6% presenting complications. No ICU admissions or fatalities were attributed to QF. In contrast to other studies [6–9, 27], the earliest recruitment and treatment could explain the lowest number of admissions, complications, and fatalities observed.

In conclusion, the most frequent causes of non-focalized fever in this region are zoonoses, specifically MT followed by QF. Given the patchy distribution of QF, it is essential to define public health strategies targeting active emission sources. The absence of cases in certain municipalities suggests the effectiveness of public health measures implemented. Concerning MT, our results highlight its significant underestimation as a disease. There is a compelling need to establish an interdisciplinary epidemiological surveillance network. Additionally, reservoir and vector control measures, coupled with prompt case identification for appropriate therapeutic intervention to mitigate potential complications and mortality risks, should be implemented. While MT has been overlooked, the significantly high incidences warrant attention. Uncertainty persists regarding its potential correlation with climate change or the expanding reservoir population in the Canary Islands. Furthermore, the possibility of MT being a re-emerging disease influenced by uncontrolled variables cannot be dismissed. Rigorous case registration is paramount for effective epidemiological control.

## Supplementary information

Below is the link to the electronic supplementary material.

Supplementary file1 (PDF 749 KB)

Supplementary file2 (PDF 53 KB)

Supplementary file3 (XLSX 24 KB)

Supplementary file4 (PDF 390 KB)

Supplementary file5 (PDF 248 KB)

Supplementary file6 (PDF 573 KB)

## Data Availability

Data is provided within the supplementary information.
