# Supplementary material for: Murine typhus as the leading cause of non-focalized fever in the Canary Islands
Source: Eur J Clin Microbiol Infect Dis. 2024 Nov 29;44(2):323–32. doi: 10.1007/s10096-024-04976-8 (PMC11754304; doi:10.1007/s10096-024-04976-8)
Supplement: Supplementary file 1 — Supplementary file1 (PDF 749 KB) [file 10096_2024_4976_MOESM1_ESM.pdf]

Servicio Canario de la Salud

CONVENIO DE COOPERACIÓN ENTRE LA UNIVERSITAT DE BARCELONA, LA UNIVERSIDAD DE LA LAGUNA Y EL SERVICIO CANARIO DE LA SALUD PARA LA EJECUCIÓN DEL PROYECTO DE INVESTIGACIÓN SOBRE FIEBRE DE DURACIÓN INTERMEDIA EN LAS ISLAS DE LA PALMA Y EL HIERRO.

De una parte, **la Universidad de Barcelona**, y en su nombre y representación el Sr. Joan Elías García, Rector Magnífico, en virtud del nombramiento por Decreto 329/2016, de 13 de diciembre (DOGC 7267, de 15 de diciembre), en ejercicio de las competencias que están previstas en el Estatuto de la Universidad de Barcelona aprobado por Decreto 246/2003, de 8 de octubre (DOGC núm. 3993, de 22 de octubre).

De otra parte, la **Universidad de La Laguna**, con C.I.F. Q 3818001D, y domicilio en la calle Molinos de Agua s/n, CP 38270, de San Cristóbal de La Laguna y en su nombre y representación, D. Ernesto Pereda De Pablo, con N.I.F. 43.809.972-D, Vicerrector de Investigación, Transferencia y Campus Santa Cruz y Sur, con poderes suficientes para la celebración de este acto, conforme a la resolución de la Rectora de fecha 27 de junio de 2019 (BOC nº131 de 10/07/2019, resolución tercera), por la que se aprueban la delegación de competencias de la Rectora y las suplencias de la Universidad de La Laguna, poderes que no le han sido derogados ni modificados.

Y de otra, el **Servicio Canario de la Salud**, y en su nombre y representación, D. Antonio José Olivera Herrera, nombrado para la suplencia temporal por Decreto 29/2020, de 26 de marzo (BOC nº 62 de 27.03.2020), en el ejercicio de las competencias delegadas por la Consejería de Sanidad mediante Orden de 29 de julio de 1997 (BOC Nº 113. Viernes 29 de Agosto de 1997).

Las partes, en el ejercicio de las funciones que les han sido legalmente atribuidas, reconociéndose recíprocamente la capacidad legal necesaria,

EXPONEN

I.- La Universitat de Barcelona, como institución de derecho público, tiene atribuida, entre otras, la función de colaborar con las administraciones públicas, instituciones y entidades privadas con la finalidad de elaborar, participar y desarrollar planes de acciones que contribuyan al progreso de la ciencia, de la difusión de la cultura y el desarrollo de la sociedad.

II.- La Universidad de La Laguna es una Institución de derecho público que rige por sus Estatutos y por lo contenido en la Ley Orgánica 6/2001, de 21 de diciembre de Universidades y que, con arreglo a lo dispuesto en los artículos 2 y 3 de sus Estatutos, tiene entre sus fines contribuir a la creación y desarrollo del conocimiento a través de la investigación, la discusión, la reflexión y la crítica, al igual que formar profesionales en los campos de las ciencias, las tecnologías, las artes y las letras e inspirar el avance tecnológico orientado a mejorar las condiciones y calidad de vida del entorno social y para ello, fomentará relaciones de intercambio y colaboración con organismos e instituciones académicas, culturales y científicas tanto nacionales como extranjeros.

Este documento incorpora firma electrónica, y es copia auténtica de un documento electrónico archivado por la ULL según la Ley 39/2015.
Su autenticidad puede ser contrastada en la siguiente dirección https://sede.ull.es/validacion/

Identificador del documento: 2483783 Código de verificación: SSH97Xun

Firmado por: Ernesto Pereda de Pablo Fecha: 20/05/2020 13:18:35
UNIVERSIDAD DE LA LAGUNA

En la dirección https://sede.gobcan.es/apjs/verifica_doc puede ser comprobada la autenticidad de esta copia, mediante el número de documento electrónico siguiente:
06hRIWtMLCkqSjtjsILfyf7WU3xVpNOuV

III.- La estructura sanitaria pública canaria, a través de las Administraciones públicas en el ámbito de sus respectivas competencias tiene atribuidas funciones de investigación en el mundo de las ciencias de la salud así como la formación continuada del personal sanitario (art. 23.3 de la Ley 11/1994, de 26 de julio, de Ordenación Sanitaria de Canarias).

En este sentido, corresponde al Servicio Canario de la Salud fomentar la investigación en los diferentes niveles de atención del Sistema de Salud (art. 110.a). Con carácter específico, y entre otros órganos o agentes del Sistema Canario de Salud, tales funciones corresponden a Hospitales (art. 82). A tal fin este organismo podrá establecer convenios con universidades, colegios y asociaciones profesionales y con otras instituciones públicas y privadas de carácter científico y cultural (art. 111).

IV.- Es intención del Servicio Canario de Salud, de la Universidad de la Laguna, a través del equipo de investigación que dirige Dña. Emma Carmelo Pascual del Instituto Universitario de Enfermedades Tropicales y Salud Pública de Canarias, y del Dr. Carlos Ascaso Terren, profesor titular del Departament de Fonaments Clínics, de la Facultat de Medicina i Ciències de la Salut, campus Clínic, de la Universitat de Barcelona, colaborar en el desarrollo del proyecto de investigación titulado *«Fiebre de Duración Intermedia en la Isla de La Palma y la isla de El Hierro»*.

V.- La fiebre de duración intermedia (FDI) se define como fiebre mayor de 38ºC de 7 a 28 días de duración que permanece sin diagnóstico a pesar de una correcta anamnesis, exploración física y pruebas complementarias de rutina. Los agentes etiológicos más frecuentes de FDI pertenecen a los géneros: *Rickettsia, Ehrlichia, Orienta y Coxiella.* No existen estudios que hayan determinado el perfil etiológico de FDI en las islas Canarias aunque si extensa bibliografía sobre fiebre Q y, en los últimos años sobre tifus murino, las cuales figuran entre las causas más frecuentes de FDI. A nivel internacional también se han reportado casos de FDI en viajeros procedentes de las islas Canarias.

La hipótesis de partida del estudio implica que un elevado porcentaje de casos de FDI que se detectan en las islas de El Hierro y La Palma se deben a infecciones por bacterias emergentes como las pertenecientes a los géneros *Rickettsia, Ehrlichia, Anaplasma y Bartonella.* Por lo tanto, el objetivo general de este estudio es la identificación de las causas infecciosas de FDI en las islas de la Palma y el Hierro y su incidencia así como la determinación de su perfil epidemiológico, con el fin de identificar y validar predictores de riesgo de complicaciones y/o hospitalización.

VI.- El interés general del proyecto está fuera de toda duda. Las partes pretenden aunar esfuerzos para su correcta ejecución, de tal modo que la UB aporta el proyecto de investigación, el SCS aporta la parte asistencial necesaria para su ejecución, y el Instituto Universitario de Enfermedades Tropicales y Salud Pública de Canarias, de la ULL aporta las determinaciones específicas que el proyecto demanda, en las muestras obtenidas de los pacientes, necesarias para el diagnóstico.

En su virtud, las Partes reconociéndose plena capacidad desean celebrar el presente Convenio, y a tal efecto, acuerdan las siguientes:

CLÁUSULAS

Primera. OBJETO.

El objeto del presente Convenio es el de establecer las pautas de cooperación y especificar la estructura operacional para que las Partes puedan desarrollar con éxito el proyecto de investigación titulado *Fiebre de Duración Intermedia en la Isla de La Palma y la isla de El Hierro* (en adelante, el Proyecto) en su versión 2, del 22 de febrero de 2019, con código 2017_81, informado favorablemente por el Comité de Ética de la Investigación con medicamentos del Complejo Hospitalario Universitario de Canarias (Provincia de Santa Cruz de Tenerife) en su sesión del 11/04/2019), cuyo resumen figura como anexo I.

Este documento incorpora firma electrónica, y es copia auténtica de un documento electrónico archivado por la ULL según la Ley 39/2015.
Su autenticidad puede ser contrastada en la siguiente dirección https://sede.ull.es/validacion/

Identificador del documento: 2483783 Código de verificación: SSH97Xun

Firmado por: Ernesto Pereda de Pablo Fecha: 20/05/2020 13:18:35
UNIVERSIDAD DE LA LAGUNA

En la dirección https://sede.gobcan.es/apjs/verifica_doc puede ser comprobada la autenticidad de esta copia, mediante el número de documento electrónico siguiente:
06hRIWtMLCkqSjtjsILfyf7WU3xVpNOuV

Segunda. COMPROMISOS DE LA UNIVERSITAT DE BARCELONA (UB).

La Universitat de Barcelona, a través del Director del Proyecto, es responsable del diseño del proyecto y de las hipótesis de trabajo, así como de la epidemiología de campo, de las bases de datos, de los análisis estadísticos de las bases de datos y de la gestión clínica de los pacientes, obligándose a:

- Cumplir lo que se expresa en los apartados incluidos en el presente Convenio de colaboración.
- Dirigir y coordinar el proyecto.

Tercera.- COMPROMISOS DE LA UNIVERSIDAD DE LA LAGUNA (ULL)

La Universidad de la Laguna, a través de la Codirectora del Proyecto, se obliga a.

- Cumplir lo que se expresa en los apartados incluidos en el presente Convenio de colaboración.
- Realizar las purificaciones de ADN de las muestras de sangre recogidas, diseñar e implementar las técnicas específicas para el diagnóstico de laboratorio mediante la detección de ADN de los microorganismos sospechosos de causar las fiebres de duración intermedia.

Cuarta.- COMPROMISOS DEL SERVICIO CANARIO DE LA SALUD (SCS)

El Servicio Canario de Salud, a través del Servicio de Medicina Interna y el grupo de Trabajo de Atención Primaria de La Palma y El Hierro, se obliga a.

- Cumplir lo que se expresa en los apartados incluidos en el presente Convenio de colaboración.
- Reclutar los pacientes, recoger muestras de sangre, realizar las analíticas estándar de sangre en sus laboratorios, enviar las muestras a la Universidad de la Laguna y tratar a los pacientes que lo necesiten.

Quinta.- DESPLAZAMIENTOS DEL PERSONAL ENTRE LAS INSTALACIONES DE LAS PARTES.

El personal de cada una de las Partes que, para mejor cumplimiento de los objetivos del presente Acuerdo, hubiera de desplazarse y permanecer por un tiempo definido en las instalaciones de la otra Parte, conservará en todo momento su dependencia laboral o administrativa de la Parte de origen, la cual asumirá las obligaciones legales o de otro tipo de su condición. En ningún caso, dicha permanencia supondrá la existencia de relación laboral alguna entre el personal desplazado y la entidad receptora, pues ambos entienden y aceptan que las actividades que en ésta última se realicen tienen por único objeto facilitar el desarrollo del Proyecto con éxito y afectan exclusivamente a la misma.

Asimismo, el personal desplazado deberá someterse, durante su permanencia en el recinto y dependencias de la otra Parte, a todas las normas de trabajo y régimen interior que se apliquen en la misma, siendo los responsables del Proyecto, tanto por parte de la Universitat de Barcelona como por parte de la Universidad de la Laguna y del Hospital General de la Palma, los encargados de velar por el cumplimiento de dichas normas.

Sexta. RESPONSABLES DE LA EJECUCIÓN DEL PROYECTO Y FORMA DE CONTACTO.

Las personas responsables de la correcta ejecución del proyecto y la forma de contacto, en función de que se trate de comunicaciones científico-técnicas o de otra índole, son las que figuran en el anexo II de este convenio.

Este documento incorpora firma electrónica, y es copia auténtica de un documento electrónico archivado por la ULL según la Ley 39/2015.
Su autenticidad puede ser contrastada en la siguiente dirección https://sede.ull.es/validacion/

Identificador del documento: 2483783 Código de verificación: SSH97Xun

Firmado por: Ernesto Pereda de Pablo Fecha: 20/05/2020 13:18:35
UNIVERSIDAD DE LA LAGUNA

En la dirección https://sede.gobcan.es/apjs/verifica_doc puede ser comprobada la autenticidad de esta copia, mediante el número de documento electrónico siguiente:
06hRIWtMLCkqSjtjsILfyf7WU3xVpNOuV

Séptima. CONFIDENCIALIDAD.

1.- Se considerará información confidencial toda la información científica y técnica, datos, propuestas, esquemas, dibujos, planes de trabajo, informes, métodos, diseños, estudios u otras materias relativas al Proyecto; hasta ahora y en adelante suministrada o revelada directa o indirectamente por cualquiera de las Partes, excepto que dicha información científica y técnica, datos, propuestas, esquemas, planes de trabajo, etc.:

a. Estuviera en posesión del receptor, y se pudiera demostrar, antes de que la misma fuese revelada por la otra Parte.
b. Haya sido adquirida por el receptor de otros que no tengan directa ni indirectamente compromiso de confidencialidad con la otra Parte.
c. Lleguen a ser de dominio público sin culpa o participación del receptor, por publicaciones u otras vías.

2.- Las Partes no usarán ninguna información confidencial sin previo consentimiento escrito de la otra, más que para el desarrollo del Proyecto, ni revelarán dicha información confidencial a otra persona o corporación. Además, están de acuerdo en hacer todos los esfuerzos necesarios para que sus empleados y otras personas que, por necesidad, pudieran tener acceso a algún aspecto de dicha información confidencial, mantengan la misma estricta confidencialidad.

3.- Toda información proporcionada por una de las Partes será devuelta en el momento en que deje de ser utilizada y, en cualquier caso, siempre que sea solicitado.

4.- El compromiso de secreto estará en vigor indefinidamente a partir de la finalización del presente convenio.

Octava. PROTECCIÓN DE DATOS PERSONALES.

1.- Cada una de las partes se compromete a cumplir con la legislación vigente en materia de protección de datos de carácter personal, de acuerdo con el Reglamento (UE) 2016/679, del Parlamento Europeo y del Consejo, de 27 de abril de 2016, relativo a la protección de las personas físicas en lo que afecta al tratamiento de los datos personales y su libre circulación, la

Ley Orgánica 3/2018, de 5 de diciembre, de Protección de Datos Personales y garantía de los derechos digitales (LOPD-GDD) y demás normativa de aplicación.

2.- Los datos personales que figuran en el texto del convenio serán tratados por cada una de las partes con sujeción a lo que en dicha normativa se dispone.

En este sentido, la información exigida en el artículo 13 del RGPD y art. 11 de la LOPD-GDD, figura en el anexo III, que las partes declaran haber leído y haber informado a las personas vinculadas a ellas, previamente a la firma del convenio.

3.- Respecto a los datos personales de los pacientes del Servicio Canario de la Salud a los que, para la correcta ejecución del convenio, sea necesario que acceda y trate el personal de la ULL ésta tendrá la condición de encargado del tratamiento. A tal fin, en los términos del artículo 28 del Reglamento General de Protección de Datos, ambas partes suscriben, mediante la firma de este convenio, el correspondiente encargo de tratamiento que figura como anexo IV.

Sin perjuicio de otras medidas de seguridad que en el contrato de encargo de tratamiento se determinen, las partes se comprometen a que los datos a los que sea necesario acceder serán seudonimizados.

Este documento incorpora firma electrónica, y es copia auténtica de un documento electrónico archivado por la ULL según la Ley 39/2015.
Su autenticidad puede ser contrastada en la siguiente dirección https://sede.ull.es/validacion/

Identificador del documento: 2483783 Código de verificación: SSH97Xun

Firmado por: Ernesto Pereda de Pablo Fecha: 20/05/2020 13:18:35
UNIVERSIDAD DE LA LAGUNA

En la dirección https://sede.gobcan.es/apjs/verifica_doc puede ser comprobada la autenticidad de esta copia, mediante el número de documento electrónico siguiente:
06hRIWtMLCkqSjtjsILfyf7WU3xVpNOuV

Si se apreciase la existencia de un peligro real y concreto para la seguridad o salud de una persona o grupo de personas, o una amenaza grave para sus derechos o sea necesaria para garantizar una adecuada asistencia sanitaria, podrá procederse a la reidentificación de los datos de origen, contactando para ello con la responsable del proyecto en el Hospital de La Palma (Tfno. 922 18 51 60).

Novena. PROPIEDAD Y EXPLOTACIÓN DE LOS RESULTADOS

Los derechos de propiedad intelectual, industrial, los métodos de trabajo, tecnología, etc. (conjuntamente, el conocimiento); pertenecientes a cualquiera de las Partes antes del comienzo del Proyecto de colaboración establecido en el presente Acuerdo y, asimismo, aquellos que siendo propiedad de terceros hubieran sido transferidos a una de las Partes o sobre los que una de las Partes ostente derechos de uso en virtud de Acuerdos alcanzados con terceros, continuarán siendo propiedad exclusiva de sus titulares y no podrán ser utilizados por las otras fuera del ámbito del Proyecto objeto del presente Acuerdo, y quedarán sujetos a los Acuerdos bilaterales de Licencia, Sublicencia o Cesión de Uso que las Partes suscriban.

Siempre corresponderá a los investigadores que hayan participado en la generación del conocimiento todos los derechos morales de la propiedad intelectual, y en especial el de ser reconocidos como sus autores.

Cualquier invención o resultado realizados por cualquiera de las Partes de forma individual, estando en vigor el presente Acuerdo, y como resultado del desarrollo del mismo, será propiedad de dicha Parte.

Décima. PROTECCIÓN Y APROVECHAMIENTO DEL CONOCIMIENTO

Las Partes se comprometen a facilitar una protección efectiva y adecuada del conocimiento susceptible de aplicabilidad industrial o comercial.

Las Partes se obligan a aprovechar los resultados que poseen y/o a aprovecharlos en otras actividades de investigación o de explotación, de acuerdo con sus intereses.

Undécima. DERECHOS DE ACCESO PARA LA EXPLOTACIÓN DE LOS RESULTADOS

Las Partes podrán explotar o encargar que se exploten los resultados y los derechos adquiridos de conformidad con lo establecido en el presente Convenio. La decisión de esta explotación será comunicada por la Parte interesada al resto de las Partes.

En el caso de que una Parte esté interesada en la explotación de los resultados que sean propiedad completa o parcial de otra Parte, ambas podrán acordar la correspondiente licencia en Contrato de Transferencia, previo al inicio de la explotación.

Una vez concluido el Proyecto, las Partes pactan expresamente que cualquier investigación que fuera a efectuarse sobre los materiales o principios que han sido objeto de estudio, se efectuará con la intervención de las mismas, a cuyo fin deberá notificar una de las Partes a la otra el correspondiente Proyecto entendiéndose que si no es rechazado de forma expresa, es deseo de la otra abordarlo de forma conjunta.

Este documento incorpora firma electrónica, y es copia auténtica de un documento electrónico archivado por la ULL según la Ley 39/2015.
Su autenticidad puede ser contrastada en la siguiente dirección https://sede.ull.es/validacion/

Identificador del documento: 2483783 Código de verificación: SSH97Xun

Firmado por: Ernesto Pereda de Pablo Fecha: 20/05/2020 13:18:35
UNIVERSIDAD DE LA LAGUNA

En la dirección https://sede.gobcan.es/apjs/verifica_doc puede ser comprobada la autenticidad de esta copia, mediante el número de documento electrónico siguiente:
06hRIWtMLCkqSjtjsILfyf7WU3xVpNOuV

Duodécima. TRANSPARENCIA Y ACCESO A LA INFORMACIÓN PÚBLICA

1.- El Convenio suscrito podrá ser puesto a disposición de los ciudadanos en los correspondientes Portales de Transparencia en aplicación de lo dispuesto en la normativa estatal y autonómica sobre transparencia y acceso a la información pública.

2.- La información elaborada como consecuencia de la ejecución del convenio tiene la consideración de información pública.

Ante las solicitudes de acceso que pudieran presentar los ciudadanos, y sin perjuicio de la obligación de efectuar las valoraciones y ponderaciones que pudieran proceder, las partes deberán tener en cuenta que la misma establece la posibilidad de limitar dicho derecho cuando acceder a la información pueda suponer un perjuicio para el secreto profesional y la propiedad intelectual e industrial (art. 14.1.j de la Ley 19/2013, de 9 de diciembre, de transparencia, acceso a la información pública y buen gobierno).

Decimotercera. USO DE LA IMAGEN INSTITUCIONAL

La firma del presente Convenio no autoriza a la Universidad de la Laguna ni al Hospital General de la Palma a hacer uso del logotipo ni de la marca UB, salvo autorización expresa y por escrito de la Secretaria General de la Universitat de Barcelona.

Decimocuarta. COMISIÓN DE SEGUIMIENTO

1. Se crea una Comisión de Seguimiento del convenio que estará compuesto por dos representantes de cada una de las partes.

a) Por la Universitat de Barcelona, el Dr. Carlos Ascaso Terren y la Dra. Rosa Mari Abellana Sangra, o persona en quien deleguen.

b) Por la Universidad de La Laguna, Dª Emma Carmelo Pascual y José Antonio Pérez Pérez, o persona en quien deleguen.

c) Por el Servicio Canario de la Salud, Dª Mónica Vélez Tobarias (HGLP) y Dª Ana Mª Torres Vega, o persona en quien deleguen.

En caso de que alguno de los miembros de la comisión se desvincule de la institución a la que representa corresponderá a las partes firmantes la desinación de las personas que les sustituyan.

2. Corresponde a la Comisión de Seguimiento:

La vigilancia y control de las actuaciones previstas en el convenio.

- El dictado de instrucciones necesarias para asegurar su adecuada realización.
- El informe a las partes de las incidencias acaecidas durante la ejecución del convenio o la propuesta razonada de su modificación.
- Acordar la modificación del convenio en el supuesto previsto en el párrafo primero de la cláusula decimosexta.
- Emitir un informe anual y otro final de valoración del grado de cumplimiento del convenio.
- Solucionar de manera consensuada las discrepancias relativas a la interpretación y aplicación del convenio.

3. La comisión paritaria se reunirá, con carácter ordinario 2 veces al año. Con carácter extraordinario podrá reunirse cuando cualquiera de los miembros de la misma lo solicite.

Este documento incorpora firma electrónica, y es copia auténtica de un documento electrónico archivado por la ULL según la Ley 39/2015.
Su autenticidad puede ser contrastada en la siguiente dirección https://sede.ull.es/validacion/

Identificador del documento: 2483783 Código de verificación: SSH97Xun

Firmado por: Ernesto Pereda de Pablo Fecha: 20/05/2020 13:18:35
UNIVERSIDAD DE LA LAGUNA

En la dirección https://sede.gobcan.es/apjs/verifica_doc puede ser comprobada la autenticidad de esta copia, mediante el número de documento electrónico siguiente:
06hRIWtMLCkqSjtjsILfyf7WU3xVpNOuV

Decimoquinta. PLAZO DE VIGENCIA DEL CONVENIO

El Convenio entrará en vigor el momento de su firma y tendrá una duración de cuatro años.

En cualquier momento antes de la finalización de dicho plazo, los firmantes del convenio podrán acordar unánimemente su prórroga por un periodo de hasta cuatro años adicionales o su extinción.

Decimosexta. RÉGIMEN DE MODIFICACIÓN

La Comisión de Seguimiento del Convenio podrá, en función de los resultados obtenidos, acordar la modificación del Proyecto, añadiendo los subproyectos que se consideren necesarios para la correcta ejecución del mismo.

Cualquier otra modificación del convenio exigirá la tramitación de la correspondiente adenda.

Decimoséptima. CAUSAS Y EFECTOS DE LA RESOLUCIÓN DEL CONVENIO.

El convenio se resolverá cuando concurra alguna de las causas previstas en el art. 51.2 de la Ley 40/2015, con los efectos establecidos en el art. 52 de dicha norma.

Decimooctava, NATURALEZA ADMINISTRATIVA Y RÉGIMEN JURÍDICO DEL CONVENIO

Las partes colaborarán en todo momento de acuerdo con los principios de buena fe y eficacia para que el Proyecto pueda ser realizado con éxito.

Este convenio tiene carácter administrativo y se regirá por sus propios términos y condiciones, así como por lo establecido con carácter básico en la Ley 40/2015, de 1 de octubre, de Régimen Jurídico del Sector Público. y en el Decreto territorial 11/2019, de 11 de febrero, por el que se regula la actividad convencional y se crean y regulan el Registro General Electrónico de Convenios del Sector Público de la Comunidad Autónoma y el Registro Electrónico de Órganos de Cooperación de la Administración Pública de la Comunidad Autónoma de Canarias.

Los principios previstos en la legislación estatal en materia de contratos del sector público se aplicarán a los efectos de resolver las dudas y lagunas que pudieran surgir en relación con su interpretación y aplicación.

Decimonovena.- ORDEN Y ÓRGANO JURISDICCIONAL COMPETENTE

Las cuestiones litigiosas que puedan surgir en la interpretación y cumplimiento del presente Convenio, y que no hayan podido ser resueltas por la comisión de seguimiento, vigilancia y control, serán de conocimiento y competencia del Orden Jurisdiccional Contencioso-Administrativo.

Y para que conste a los efectos oportunos, en prueba de conformidad, las partes firman el presente Convenio.

Este documento incorpora firma electrónica, y es copia auténtica de un documento electrónico archivado por la ULL según la Ley 39/2015.
Su autenticidad puede ser contrastada en la siguiente dirección https://sede.ull.es/validacion/

Identificador del documento: 2483783 Código de verificación: SSH97Xun

Firmado por: Ernesto Pereda de Pablo
UNIVERSIDAD DE LA LAGUNA
Fecha: 20/05/2020 13:18:35

En la dirección https://sede.gobcan.es/apjs/verifica_doc puede ser comprobada la autenticidad de esta copia, mediante el número de documento electrónico siguiente:
06hRIWtMLCkqSjtjsILfyf7WU3xVpNOuV

ANEXO I

RESUMEN DEL PROYECTO DE INVESTIGACIÓN

Título del estudio:

Fiebre de Duración Intermedia en las islas de La Palma y El Hierro

Plataforma de Investigación en Fiebre de Duración Intermedia entre la Universidad de Barcelona, el *Instituto Universitario de Enfermedades Tropicales y Salud Pública de Canarias* de la Universidad de La Laguna, el Área de Salud de La Palma y de la isla de El Hierro.

CÓDIGO: plataformafdi19

Versión 2 del 22 de febrero de 2019

EQUIPO INVESTIGADOR:
Mónica Vélez Tobarias
FEA Medicina Interna. Hospital General de la Palma (HGLP)
Ana Mª Torres Vega
FEA Medicina Interna. Hospital Insular Ntra. Sra. de los Reyes (HINSR)
Dr. Carlos Ascaso Terrén
Departament de Fonaments Clínics, UB - Línea de Investigación, Salud Internacional.
Dra. Emma Carmelo Pascual.
Departamento de Obstetricia y Ginecología, Pediatría, Medicina Preventiva y Salud Pública, Toxicología, Medicina Legal y Forense y Parasitología. Instituto Universitario de Enfermedades Tropicales y Salud Pública de Canarias (IUETSPC). Universidad de la Laguna (ULL).

Este documento incorpora firma electrónica, y es copia auténtica de un documento electrónico archivado por la ULL según la Ley 39/2015.
Su autenticidad puede ser contrastada en la siguiente dirección https://sede.ull.es/validacion/

Identificador del documento: 2483783 Código de verificación: SSH97Xun

Firmado por: Ernesto Pereda de Pablo Fecha: 20/05/2020 13:18:35
UNIVERSIDAD DE LA LAGUNA

En la dirección https://sede.gobcan.es/apjs/verifica_doc puede ser comprobada la autenticidad de esta copia, mediante el número de documento electrónico siguiente:
06hRIWtMLCkqSjtjsILfyf7WU3xVpNOuV

ÍNDICE

1. Preguntas de investigación e

   Introducción 2

2. Materiales y métodos 4

3. Zoonosis emergentes como potenciales causas de Fiebre de Duración

   Intermedia y sus características principales 7

4. Convenio de colaboración 10

5. Aspectos éticos 10

6. Bibliografía relevante 12

Este documento incorpora firma electrónica, y es copia auténtica de un documento electrónico archivado por la ULL según la Ley 39/2015.

Su autenticidad puede ser contrastada en la siguiente dirección https://sede.ull.es/validacion/

Identificador del documento: 2483783 Código de verificación: SSH97Xun

Firmado por: Ernesto Pereda de Pablo Fecha: 20/05/2020 13:18:35

UNIVERSIDAD DE LA LAGUNA

En la dirección https://sede.gobcan.es/apjs/verifica_doc puede ser comprobada la autenticidad de esta copia, mediante el número de documento electrónico siguiente:

06hRIWtMLCkqSjtjsILfyf7WU3xVpNOuV

TÍTULO DEL PROYECTO

Fiebre de Duración Intermedia en la islas de La Palma y El Hierro

1. PREGUNTAS DE INVESTIGACIÓN E INTRODUCCIÓN

(1) ¿Cuáles son las causas infecciosas de FDI en las islas de La Palma y El Hierro y su incidencia?

(2) ¿Cuáles son las características diferenciales entre las distintas etiologías infecciosas más frecuentes de FDI?

La fiebre de duración intermedia (FDI) se define como fiebre mayor de 38ºC de 7 a 28 días de duración que permanece sin diagnóstico a pesar de una correcta anamnesis, exploración física y pruebas complementarias de rutina, incluyendo, radiografía de tórax, sedimento de orina, hemograma y bioquímica básica. El uso de este término aparece por primera vez en España el año 1992 siendo, posteriormente, su uso extendido a nivel nacional desde 1999 gracias a los trabajos de Bernabeu-Wittel[1-2]. Sin embargo, no se menciona este término a nivel internacional en búsquedas realizadas a través de medline. A pesar de ser una de las causas más frecuentes de fiebre en la práctica médica diaria, este concepto tampoco está incluido en los tratados clásicos de Medicina Interna que sólo distinguen entre fiebre de corta duración (FCD) o fiebre aguda y, fiebre de origen desconocido (FOD) [4-5]. El trabajo más amplio de FDI realizado por el grupo de Espinosa et al., en Sevilla, muestra un claro predominio de enfermedades infecciosas, entre las más frecuentes la fiebre Q, brucellosis, tifus murino, fiebre botonosa mediterránea y, menos frecuentes, la fiebre tifoidea, síndrome mononucleósico (incluyendo VEB, CMV y toxoplasmosis) y leptospirosis, un 18.8% de casos son de etiología desconocida y otro porcentaje mucho menor de causas no infecciosas[3]. El espectro etiológico de FDI no es estable, varia en el tiempo y en función de la localidad pero se carece de información por la limitación de estudios en las distintas regiones de España. Estudios similares se han realizado en la Rioja[6]. Los agentes etiológicos más frecuentes de FDI pertenecen a la familia Rickettsiae que incluye a los distintos géneros: *Rickettsia, Ehrlichia, Orienta y Coxiella*[7-8.]

Este documento incorpora firma electrónica, y es copia auténtica de un documento electrónico archivado por la ULL según la Ley 39/2015.
Su autenticidad puede ser contrastada en la siguiente dirección https://sede.ull.es/validacion/

Identificador del documento: 2483783 Código de verificación: SSH97Xun

Firmado por: Ernesto Pereda de Pablo Fecha: 20/05/2020 13:18:35
UNIVERSIDAD DE LA LAGUNA

En la dirección https://sede.gobcan.es/apjs/verifica_doc puede ser comprobada la autenticidad de esta copia, mediante el número de documento electrónico siguiente:
06hRIWtMLCkqSjtjsILfyf7WU3xVpNOuV

No existen estudios que hayan determinado el perfil etiológico de FDI en las islas Canarias aunque si extensa bibliografía sobre fiebre Q[9-15] y, en los últimos años sobre tifus murino[16-20], las cuales figuran entre las causas más frecuentes de FDI. A nivel internacional también se han reportado casos de FDI en viajeros procedentes de las islas Canarias[21-22].

Por otro lado, hay numerosos estudios que describen nuevos agentes emergentes en España causantes de FDI como *Rickettsia felis* [23-26], Ehrlichiosis y anaplasmosis humana[27], *Rickettsia monacensis*[28], *Rickettsia sibirica mongolitimonae*[29], *Rickettsia slovaca*[30.] Los 5 primeros casos de *Rickettsia felis* en las islas Canarias fueron descritos en 2005 por el grupo de Pérez-Arellano[23]. Sin embargo, no se ha podido demostrar presencia de *Anaplasma phagocytophilum*[31] en las islas en humanos. La fiebre Q causada por *Coxiella burnetii* es la causa de FDI más frecuente por lo que se plantea el uso empírico de doxiciclina como tratamiento para esta entidad hasta su diagnóstico serológico definitivo[32].

En la isla de Tenerife hay publicado un estudio clínico-epidemiológico y de características diferenciales de fiebre Q con tifus murino, reportándose 47 y 32 casos, respectivamente, recopilados entre el 1998-2002[17]. Por otro lado, hay publicadas series de casos y casos clínicos aislados referentes a complicaciones clínicas del tifus murino como afectación renal[18], neumonía[19], vasculitis[17] y uveítis anterior[20] en las islas Canarias. Hasta el momento no ha habido estudios que hayan permitido detectar indicadores de riesgo de hospitalización, complicación ni diferencias clínicas ni laboratoriales de cada entidad nosológica previo a su diagnóstico serológico.

Basándonos en que la FDI es el tipo de fiebre por el que más se consulta en la práctica médica diaria además, de su espectro cambiante en tiempo y espacio y, la aparición continua de nuevos agentes etiológicos, nos motiva a profundizar en su estudio en la isla de la Palma y el Hierro, dos de las islas Canarias, caracterizadas todas ellas por su singularidad geográfica. Esta información nos permitiría consensuar protocolos de manejo diagnóstico-terapéutico que fueran costo-efectivos en las islas e, incluso, orientarnos a una mayor vigilancia epidemiológica y prevención de sus causas ya que, en su mayoría, se trata de zoonosis relacionadas con vectores transmisores en el ambiente.

Los estudios respecto a complicaciones y criterios de hospitalización de estas entidades son muy escasos por lo que identificar predictores de riesgo de ambos influiría

Este documento incorpora firma electrónica, y es copia auténtica de un documento electrónico archivado por la ULL según la Ley 39/2015.
Su autenticidad puede ser contrastada en la siguiente dirección https://sede.ull.es/validacion/

Identificador del documento: 2483783 Código de verificación: SSH97Xun

Firmado por: Ernesto Pereda de Pablo Fecha: 20/05/2020 13:18:35
UNIVERSIDAD DE LA LAGUNA

En la dirección https://sede.gobcan.es/apjs/verifica_doc puede ser comprobada la autenticidad de esta copia, mediante el número de documento electrónico siguiente:
06hRIWtMLCkqSjtjsILfyf7WU3xVpNOuV

directamente en la calidad asistencial de los pacientes y, posiblemente, la disminución de costes sanitarios en el manejo de esta patología.

Todo ello supondría un gran avance científico con potencial impacto en el campo de la Salud Pública para esta entidad nosológica, relativamente joven, definida en España.

Impacto: disminuir los diagnósticos inespecíficos con dificultad de tratamiento; introducir como diagnóstico específico patologías no incluidas en la isla; identificar predictores de riesgo y hospitalización por patología; aumentar los tratamientos específicos y el éxito en el tratamiento; establecer programas preventivos de disminución de incidencia de dichas patologías.

Hipótesis

(1) La fiebre Q y el tifus murino son las causas de FDI con mayor tasa en la isla de La Palma y la isla de El Hierro.
(2) Existe un perfil epidemiológico para la FDI.
(3) Existe un perfil etiológico de causas infecciosas FDI en las islas de La Palma y El Hierro distinto al descrito en otras áreas de España.

Objetivos

(1) Definir un perfil etiológico de causas infecciosas de FDI en la isla de La Palma y El Hierro.
(2) Describir características epidemiológicas de los casos de FDI.
(3) Definir si existe un perfil clínico/laboratorio distintivo de cada patología.

2. MATERIALES Y MÉTODOS

Canarias forma parte de una de las diecisiete comunidades autónomas de España y, una de las regiones ultraperiféricas de la Unión Europea. El archipiélago canario está conformado por ocho islas de origen volcánico, cinco islotes y ocho roques. Su clima está influenciado por la interacción de corrientes marinas del banco sahariano y los vientos alisios, resultando ser suave y homogéneo a lo largo del año, de tipo subtropical. Canarias ha sido considerada históricamente un puente de conexión entre tres continentes: Europa, África y América.

Este documento incorpora firma electrónica, y es copia auténtica de un documento electrónico archivado por la ULL según la Ley 39/2015.
Su autenticidad puede ser contrastada en la siguiente dirección https://sede.ull.es/validacion/

Identificador del documento: 2483783 Código de verificación: SSH97Xun

Firmado por: Ernesto Pereda de Pablo Fecha: 20/05/2020 13:18:35
UNIVERSIDAD DE LA LAGUNA

En la dirección https://sede.gobcan.es/apjs/verifica_doc puede ser comprobada la autenticidad de esta copia, mediante el número de documento electrónico siguiente:
06hRIWtMLCkqSjtjsILfyf7WU3xVpNOuV

El estudio se llevará a cabo en dos de las islas Canarias, la isla de La Palma y la isla de El Hierro. La isla de la Palma es la segunda isla más occidental del Archipiélago Canario, de clima subtropical y origen volcánico, situada al norte de África, en el océano Atlántico (Latitud: 28,6 Longitud: -17,7), cerca de las costas del sur de Marruecos y el Sáhara. La distancia mínima a la costa peninsular son de unos 1000 km. Tiene una superficie de 708,33 km² (10% del territorio canario) y una población de 81.350 habitantes a fecha 1 de enero de 2017[33]. El punto más elevado de la isla es el Roque de Los Muchachos, a una altitud de 2.426 m[34].

La isla se divide en 14 municipios: al norte Garafía, Barlovento, Puntagorda y Los Sauces; al sur Fuencaliente; al este Puntallana, Santa Cruz, Breña Alta y Breña Baja, Mazo; al Oeste Tijarafe, Tazacorte, Los Llanos y en el centro el Paso. El único hospital de la isla, llamado Hospital General de la Palma (HGLP) que, atiende a toda la población, está localizado al este, en Breña Alta.

El Hierro es la isla canaria más pequeña, joven, occidental y meridional. Fue declarada por la UNESCO (*United Nations Educational, Scientific and Cultural Organization*) en el año 2000 Reserva Mundial de la Biosfera y Geoparque en 2014. Posee una gran cantidad de acantilados, costas rocosas y muy abruptas entre las que se ubican numerosas piscinas naturales y charcos. La geografía herreña en general es abrupta, presenta uno de los mayores gradientes de desnivel altitudinal de las islas Canarias, conformando un edificio piramidal de base triangular por asociación de tres dorsales volcánicas. Su punto de máxima altura es el Pico de Malpaso situado a 1501,762 m. Cuenta con una superficie de 268,71 kilómetros cuadrados (Km^2) y, según datos del Instituto Canario de Estadística (ISTAC), una población de 10.798 habitantes en 2017. Sus habitantes se distribuyen en tres municipios: Valverde (4.955 habitantes en 2017), La Frontera (4.018 habitantes en 2018) y El Pinar (1.825 habitantes en 2017) con un elevado nivel de dispersión y una densidad media de 40 hab/Km^2.

El Área de Salud de El Hierro, al cual pertenece el Hospital Insular Ntra. Sra. de los Reyes (HINSR), se compone de 2 Zonas Básicas de Salud, que cubren la totalidad del territorio insular: Zona Especial de Salud de Valverde (comprende los municipios de Valverde y El Pinar) y Zona Especial de Salud de Frontera-Valle del Golfo **(**abarca el municipio de La Frontera).

Este documento incorpora firma electrónica, y es copia auténtica de un documento electrónico archivado por la ULL según la Ley 39/2015.
Su autenticidad puede ser contrastada en la siguiente dirección https://sede.ull.es/validacion/

Identificador del documento: 2483783 Código de verificación: SSH97Xun

Firmado por: Ernesto Pereda de Pablo Fecha: 20/05/2020 13:18:35
UNIVERSIDAD DE LA LAGUNA

En la dirección https://sede.gobcan.es/apjs/verifica_doc puede ser comprobada la autenticidad de esta copia, mediante el número de documento electrónico siguiente:
06hRIWtMLCkqSjtjsILfyf7WU3xVpNOuV

Se reclutaran todos los casos con diagnóstico de FDI diagnosticados durante 12 meses consecutivos. Previamente al inicio de reclutamiento se realizará un proyecto piloto para nueva estimación de tamaño muestral ya que el actual esta basado en un estudio retrospectivo de fiebre Q y rickettiosis durante el año 2013.

Para realizar el reclutamiento de los casos se distribuirá un algoritmo de manejo diagnóstico-terapéutico, los criterios de inclusión, exclusión y los criterios de derivación de FDI a todos los Centros de Atención Primaria participantes, especialidades hospitalarias de Medicina Interna y Servicio de Urgencias Hospitalario. Los pacientes que cumplan criterio diagnóstico y criterio de inclusión firmarán el consentimiento informado aceptando la participación en el estudio, se realizarán la extracción de sangre según las instrucciones dadas y, se rellenará el cuestionario inicial con la anamnesis y datos básicos epidemiológicos. El proceso de recogida de muestras seguirá el habitual del Área de Salud de la Palma y El Hierro. Todos aquellos casos que cumplan criterio de FDI podrán recibir tratamiento antibiótico con doxiciclina según criterio médico una vez haya sido extraída la muestra de sangre. Si el resultado de serología y PCR son positivos y concordantes se considerará un caso con diagnóstico positivo. Si el resultado de serología es negativo o no concluyente el paciente será remitido al Servicio de Medicina Interna para ampliar el estudio según sospecha diagnóstica específica y contexto epidemiológico.

Técnicas serológicas empleadas: Las técnicas serológicas utilizadas y los puntos de corte son: IgM ≥1/64 o, IgG≥1/1024 mediante inmunofluorescencia indirecta (Virion, Richland, USA en 1983-1989 y Vitaltech en 2004-2005) para *Coxiella burnetti*; IgM≥1/80 mediante inmunofluorescencia indirecta (Pasteur Laboratories, Marnes, la Coquete, Francia desde 1986 y bioMérieux desde 1987) para *Rickettsia typhi* y *Rickettsia conorii*; técnica de aglutinación con Rosa Bengala (bioMérieux, Lyon, Francia) para *Brucella melitensis*; IgM positiva mediante IQL (inmunoquimioluminiscencia) para CMV y IgM positivas mediante método ELISA para VEB. Los puntos de corte utilizados son compatibles con infección aguda o reciente.

Técnicas de PCR empleadas: un volumen de cada muestra de sangre congelada serán sometidos a purificacion de ADN total mediante Kit comerciales (QiampDNA mini kit de Qiagen o similar). El diagnóstico molecular se realizará mediante PCR según protocolos

Este documento incorpora firma electrónica, y es copia auténtica de un documento electrónico archivado por la ULL según la Ley 39/2015.
Su autenticidad puede ser contrastada en la siguiente dirección https://sede.ull.es/validacion/

Identificador del documento: 2483783 Código de verificación: SSH97Xun

Firmado por: Ernesto Pereda de Pablo
UNIVERSIDAD DE LA LAGUNA
Fecha: 20/05/2020 13:18:35

En la dirección https://sede.gobcan.es/apjs/verifica_doc puede ser comprobada la autenticidad de esta copia, mediante el número de documento electrónico siguiente:
06hRIWtMLCkqSjtjsILfyf7WU3xVpNOuV

establecidos y secuenciación de los fragmentos amplificados[37-40]. Las secuencias serán alineadas mediante MEGA6 y BLAST (NCBI).

El remanente de las alícuotas será destruido por el Sistema de gestión de residuos del IUETSPC una vez hayan sido procesadas.

Las variables cuantitativas se describirán mediante los estadísticos: media, mediana, desviación estándar, máximo y mínimo. Las variables cualitativas se describirán mediante las frecuencias absolutas, relativas y porcentajes. Las asociaciones entre variables se evaluarán mediante pruebas de Chi-Cuadrado, t de Student o coeficiente r de Pearson.

También se usarán modelos lineales generales que incluyan las variables de confusión requeridas en cada objetivo. Las estimaciones se harán con un intervalo de confianza del 95%. Los contrastes de hipótesis se evaluarán con un nivel de confianza del 5%. Los análisis estadísticos se realizarán con los paquetes SPSS versión 21 y R versión 3.0.2

El reclutamiento de los casos de FDI dependerá del grupo de trabajo de Atención Primaria y Servicio de Urgencias, posibles irregularidades en el sistema podrían alterar la total inclusión de nuevos casos FDI. Los pacientes que pudiesen ser atendidos en servicios sanitarios privados no serán incluidos en el estudio. Por todo ello existe el riesgo de que las incidencias se subestimen.

3. ZOONOSIS EMERGENTES COMO POTENCIALES CAUSAS DE FIEBRE DE DURACIÓN INTERMEDIA Y SUS CARACTERÍSTICAS PRINCIPALES:

A continuación se muestra la taxonomía clásica del orden *Rickettsiales* en la cual, inicialmente se incluía a *Coxiella* y *Rickettsia* dentro de la misma familia por sus características fenotípicas (Fig1). Posteriormente, la secuenciación de RNAm y del genoma completo de *Coxiella burnetti* permitió reclasificar al microorganismo dentro de la familia *Legionalleacee* en la cual se mantiene en la actualidad (Fig. 2). *Anaplasma* y *Ehrlichia*, sin embargo, en la taxonomía clásica eran clasificados en distintas familias del orden *Rickettsiales* pero en la taxonomía actual son consideradas distintos géneros de la misma familia Anaplasmataceae que, taxonómicamente siguen perteneciendo al orden Rickettsiales (alfa 1 Proteobacteria)[36].

Por otro lado, en la tabla 1, se muestran las características principales de las zoonosis más frecuentes conocidas como causa de FDI y, además, otras zoonosis emergentes según distribución espacial y vectores principales conocidos.[35]

Este documento incorpora firma electrónica, y es copia auténtica de un documento electrónico archivado por la ULL según la Ley 39/2015.
Su autenticidad puede ser contrastada en la siguiente dirección https://sede.ull.es/validacion/

Identificador del documento: 2483783 Código de verificación: SSH97Xun

Firmado por: Ernesto Pereda de Pablo Fecha: 20/05/2020 13:18:35
UNIVERSIDAD DE LA LAGUNA

En la dirección https://sede.gobcan.es/apjs/verifica_doc puede ser comprobada la autenticidad de esta copia, mediante el número de documento electrónico siguiente:
06hRIWtMLCkqSjtjsILfyf7WU3xVpNOuV

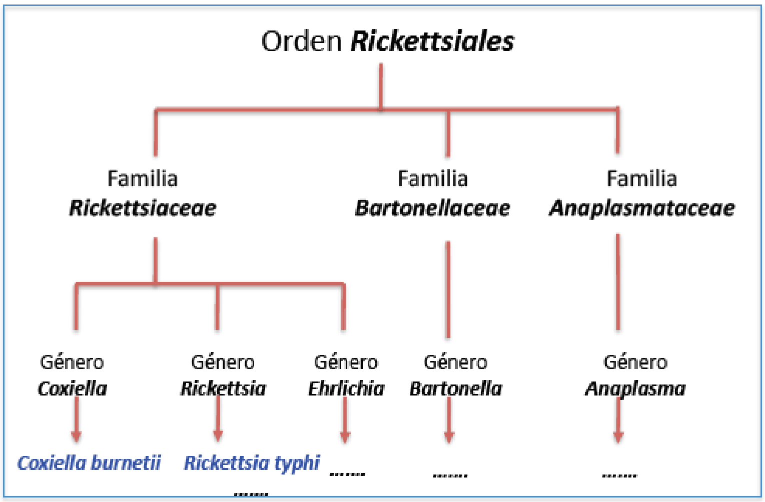

Fig 1. Taxonomía clásica de Coxiella burnetti y Rickettsia Tiphy

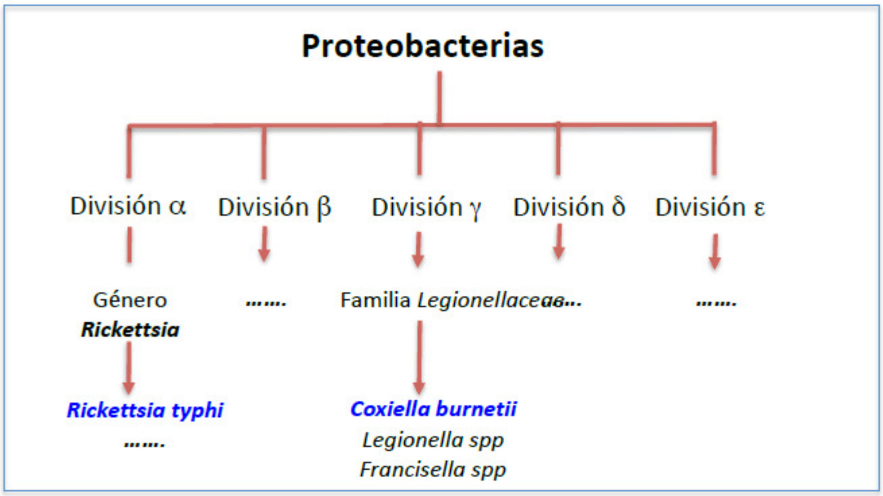

Fig 2. Taxonomía actual de *Rickettsi typhi* y *Coxiella burnetii*

Este documento incorpora firma electrónica, y es copia auténtica de un documento electrónico archivado por la ULL según la Ley 39/2015.
Su autenticidad puede ser contrastada en la siguiente dirección https://sede.ull.es/validacion/

Identificador del documento: 2483783 Código de verificación: SSH97Xun

Firmado por: Ernesto Pereda de Pablo Fecha: 20/05/2020 13:18:35
UNIVERSIDAD DE LA LAGUNA

En la dirección https://sede.gobcan.es/apjs/verifica_doc puede ser comprobada la autenticidad de esta copia, mediante el número de documento electrónico siguiente:
06hRIWtMLCkqSjtjsILfyf7WU3xVpNOuV

Tabla 1. Zoonosis más frecuentes como causa de FDI y otras zoonosis emergentes.

ENFERMEDAD	ESPECIE	VECTOR	DISTRIBUCIÓN
Fiebre Q	*C. burnetii*	Reservorio: ganado, roedores.<br><br>No hay vector.	Mundial
Tifus Murino	*R. typhi*	Pulgas (*Xenopsylla cheopis* and *Ctenocephalides felis*)	Mundial (más prevalente en zonas tropicales y subtropicales)
Fiebre manchada de pulgas	*R. felis*	Pulgas de gato (*Ctenocephalides felis*)	Mundial
FBM[1]	*R. conorii conorii,*<br><br>*R. conorii israelensis*,<br><br>*R. conorii caspia*,<br><br>*R. conorii indica*	*Rhipicephalus* spp. garrapatas	Área mediterránea,<br><br>Europa central, Rusia,<br><br>India y África (no descritos casos clínicos en Canarias)
FBM-like	*R. monacensis*	*Ixodes ricinus* garrapatas	Europa
FBM-like	*R. massiliae*	*Rhipicephalus*<br><br>*sanguineus* garrapatas	Área mediterránea,<br><br>Argentina, USA?
FBM-like	*R. aeschlimannii*	*Hyalomma marginatum*<br><br>garrapatas	Africa, Europa?
DEBONEL / TIBOLA[2]	*R. slovaca*<br><br>*R. rioja*<br><br>*R. raoultii*	*Dermacentor marginatus*<br><br>garrapatas	Europa
LAR[3]	*R. sibirica*<br><br>*mongolitimonae*	*Hyalomma* spp. y<br><br>*Rhipicephalus pusillus* garrapatas	Europa, Africa.
ATBF[4]	*R. africae*	*Amblyomma* spp. garrapata	Africa Sub-Sahariana e India
R. helvetica	*R. helvetica*	*Ixodes ricinus* garrapatas	Norte y Centro Europa, Asia

Este documento incorpora firma electrónica, y es copia auténtica de un documento electrónico archivado por la ULL según la Ley 39/2015.
Su autenticidad puede ser contrastada en la siguiente dirección https://sede.ull.es/validacion/

Identificador del documento: 2483783 Código de verificación: SSH97Xun

Firmado por: Ernesto Pereda de Pablo Fecha: 20/05/2020 13:18:35
UNIVERSIDAD DE LA LAGUNA

En la dirección https://sede.gobcan.es/apjs/verifica_doc puede ser comprobada la autenticidad de esta copia, mediante el número de documento electrónico siguiente:
06hRIWtMLCkqSjtjsILfyf7WU3xVpNOuV

infection			
Ehrlichiosis granulocítica	*E. ewingii*	*A. americanum*	Norteamérica (en perros y humanos inmunodeprimidos)
Ehrlichiosis monocítica canina	*E. canis*	Garrapata marrón del perro (*Riphicephalus sanguineus)*	Mundial
Ehrlichiosis humana monocítica	*E. chaffeensis*	*Amblyomma americanum*<br><br>*Dermacentor variabili*	Norteamérica<br><br>Centroamérica
Anaplasmosis humana granulocítica	*A. phagocytophilum*	*Ixodes ricinus*	Europa<br><br>Norteamérica<br><br>Norte de África
Bartonellosis	*B. bacilliformis*	*Hombre*	Sur América (Perú, Ecuador, Colombia)
Enf arañazo de gato	*B. henselae*	*Pulgas gato doméstico*	Mundial
Fiebre de las Trincheras	*B. quintana*	*Piojos hombre (pediculus corporis)*	Mundial

1MSF: Fiebre Botonosa Mediterránea; 2DEBONEL/TIBOLA: *Dermacentor*-borne, necrosis, erythema, lymphadenopathy/Tick-borne lymphadenopathy; 3LAR: Lymphangitis-associated rickettsiosis; 4ATBF: African tick-bite fever.

4. ASPECTOS ÉTICOS

El presente proyecto pasará los comités de ética del Hospital Universitario de Canarias.

Las muestras bilógicas se destruirán inmediatamente después de realizar las PCR de diagnóstico.

El estudio se realizará en conformidad con los principios de la Declaración de Helsinki adoptada por la 18ª Asamblea Médica Mundial, Helsinki, Finlandia en 1964 y enmendada en Tokio (1975), Venecia (1983), Hong Kong (1989), Sudáfrica (1996), Edimburgo (2000), Washington (2002), Tokio (2004), Seúl (2008), Brasil (2013); y las Leyes y Reglamentos vigentes en Europa y España.

El paciente debe otorgar su consentimiento antes de ser admitido en el estudio clínico. El médico habrá de explicar la naturaleza, propósitos y posibles consecuencias del estudio, de

Este documento incorpora firma electrónica, y es copia auténtica de un documento electrónico archivado por la ULL según la Ley 39/2015.
Su autenticidad puede ser contrastada en la siguiente dirección https://sede.ull.es/validacion/

Identificador del documento: 2483783 Código de verificación: SSH97Xun

Firmado por: Ernesto Pereda de Pablo Fecha: 20/05/2020 13:18:35
UNIVERSIDAD DE LA LAGUNA

En la dirección https://sede.gobcan.es/apjs/verifica_doc puede ser comprobada la autenticidad de esta copia, mediante el número de documento electrónico siguiente:
06hRIWtMLCkqSjtjsILfyf7WU3xVpNOuV

una manera comprensible al paciente. La información proporcionada por el médico deberá ser también registrada.

El sujeto del estudio otorgará su consentimiento, firmando el modelo correspondiente que también deberá llevar la firma del investigador.

El investigador no iniciará ninguna investigación correspondiente al estudio hasta que haya obtenido el consentimiento del paciente.

Con el fin de garantizar la confidencialidad de los datos de los pacientes participantes en el estudio, sólo tendrán acceso a los mismos el investigador y su equipo de colaboradores. Los datos serán codificados para asegurar el anonimato.

El tratamiento, la comunicación y la cesión de los datos de carácter personal de todos los sujetos participantes se ajustará a lo dispuesto en la Ley Orgánica 3/2018, de 5 de diciembre de Protección de Datos Personales y garantía de los derechos digitales, y a la aplicación de del Reglamento (UE) 2016/679 del Parlamento europeo y del Consejo de 27 de abril de 2016 de Protección de Datos (RGPD).

5. BIBLIOGRAFÍA

1. Bernabeu-Wittel M, Cordero E, Viciana P, Pachón J. **Etiología y criterios de ingreso del síndrome febril sin focalidad.** Med Clin (Barc). 1999;113:718–9.

2. Bernabeu-Wittel M, Pachón J, Alarcón A, López-Cortés LF, Viciana P, Jiménez-Mejías ME, Villanueva JL, Torronteras R, Caballero-Granado FJ. **Murine typhus as a common cause of fever of intermediate duration: a 17-year study in the south of Spain.** Arch Intern Med. 1999 Apr 26;159(8):872-6.

3. Espinosa N, Cañas E, Bernabeu-Wittel M, Martín A, Viciana P, Pachón J**. The changing etiology of fever of intermediate duration.**.Enferm Infecc Microbiol Clin. 2010 Aug-Sep;28(7):416-20. doi: 10.1016/j.eimc.2009.07.014. Epub 2010 Feb 12.

4. Tudela Hita P, Urrutia de Diego A. **Fiebre aguda y fiebre de origen desconocido.** XVI edición. En: Farreras-Rozman Medicina Interna. Elsevier España SL, 2010; 321:2574–80.

Este documento incorpora firma electrónica, y es copia auténtica de un documento electrónico archivado por la ULL según la Ley 39/2015.
Su autenticidad puede ser contrastada en la siguiente dirección https://sede.ull.es/validacion/

Identificador del documento: 2483783   Código de verificación: SSH97Xun

Firmado por: Ernesto Pereda de Pablo   Fecha: 20/05/2020 13:18:35
UNIVERSIDAD DE LA LAGUNA

En la dirección https://sede.gobcan.es/apjs/verifica_doc puede ser comprobada la autenticidad de esta copia, mediante el número de documento electrónico siguiente:
06hRIWtMLCkqSjtjsILfyf7WU3xVpNOuV

5. **Mackowiak PA, Durack DT. In: Mandell, Douglas, and Bennett's, Principles and Practice of Infectious Diseases. Mandell GL, Bennett JE, Dolin R, eds. 7th. Edition. Philadelphia: Elsevier; 2010. 51:779-89.**
6. José A. Oteo. **Fever of intermediate duration: New times, new tools and change of spectrum**. Enferm Infecc Microbiol Clin. 2010;28(7):407–408.
7. David H. Walker. **.** Chapter 38: **Rickettsiae**. 4th edition. Baron S, editor. Medical Microbiology. Galveston (TX): University of Texas medical Branch at Galeston; 1996.
8. Aránzazu Portillo and José A. Oteo. **Rickettsiosis as Threat for Traveller.** Cap 1. Current Topics in Tropical Medicina. Edited by Dr. Alfonso Rodríguez-Morales. 2012. www.intechopen.com
9. European Journal of Epidemiology 2003 March:18(3): 259-262. **Seroprevalence of infection by Coxiella burnetii in Canary Islands (Spain).** Bolaños M, Santana OE, Ángel-Moreno A, Pérez-Arellano JL, Limiñana JM, Serra-Majem L, Martín-Sánchez AM.
10. Rev Clin Esp. 1997 Jan;197(1):69. **A review of Q fever in the Canary Islands.** Pascual Velasco F.
11. Scand J Infect Dis. 1996;28(5):533-4. **Clinical presentation of acute Q fever in lanzarote (Canary Islands): a 2-year prospective study.** Pascual Velasco F(1), Borobio Enciso MV, González Lama Z, Carrascosa Porras M. (1)Service of Internal Medicine, Hospital of Laredo, Cantabria, Spain.
12. Enferm Infecc Microbiol Clin. 2003 Jan;21(1):20-3. **Q fever in Gran Canaria: 40 new cases.** Bolaños M(1), Santana OE, Pérez-Arellano JL, Angel-Moreno A, Moreno G, Burgazzoli JL, Martín-Sánchez AM.
13. An Med Interna. 1989 Oct;6(10):527-30. **Q fever on the island of La Palma. A review of 35 patients.** Millán Mon A, Argany Fajardo A, Febles Bethencourt J, González Caloca C, Vento Remedios TE, Fernández Cabrera M.
14. An Med Interna 1991 May; 8P85r9:233-4. **Prevalence of antibodies against Coxiella burnetii in Healthy population in Lanzarote (Canary Islands).** Pascual Veleasco F, Otero Ferrio I, Borobio Enciso MV.

Este documento incorpora firma electrónica, y es copia auténtica de un documento electrónico archivado por la ULL según la Ley 39/2015.
Su autenticidad puede ser contrastada en la siguiente dirección https://sede.ull.es/validacion/

Identificador del documento: 2483783 Código de verificación: SSH97Xun

Firmado por: Ernesto Pereda de Pablo *UNIVERSIDAD DE LA LAGUNA* Fecha: 20/05/2020 13:18:35

En la dirección https://sede.gobcan.es/apjs/verifica_doc puede ser comprobada la autenticidad de esta copia, mediante el número de documento electrónico siguiente:
06hRIWtMLCkqSjtjsILfyf7WU3xVpNOuV

15. An Med Inerna. 1992 Sep;9(9):428-32. **Seroprevalence of Q fever among th adult population of Lanzarote (Canary Islands).** Pascual Velasco F, Rodríguez Pérez JC, Otero Ferrio I, Borobio Enciso MV.
16. Bolaños-Rivero M, Santana-Rodríguez E, Ángel-Moreno A, Hernández-Cabrera M, Limiñana-Canal JM, Carranza-Rodríguez C, Martín-Sánchez AM, Pérez-Arellano JL. **Seroprevalence of Rickettsi typhi and Rickettsi conorii infections in the Canary Islands (Spain).** International Journal of Infectiuous Diseases. 2011; 15:481-485.
17. Miguélez M, Laynez P, Linares M, Hayek M, Abella L, Marañez I. Murine typhus in Tenerife. **Clinicoepidemiological study and differential clinical features with Q fever.** Med Clin (Barc) 2003; 121:613-5.
18. Hernández-Cabrera M, Angel-Moreno A, Santan E, Bolaños M, Frncés A, Martín-Sánchez MS, et al. **Murine typhus with renal involvement in Canary Islands, Spain.** Emerg Infect Dis 2004;10:740-3.
19. Enferm Infecc Microbiol Clin. 2012 Aug;30(7):427-8. doi: 10.1016/j.eimc.2012.02.008. Epub 2012 Apr 12. **Rickettsia typhi. A new causative agent of round pneumonia in adults.** Velasco-Tirado V, Hernández-Cabrera M, Pisos-Álamo E, Pérez-Arellano JL. Hospital Insular de Gran Canaria, Spain.
20. Enferm Infecc Microbiol Clin. 2011:29(3):232-242. **Uveitis anterior bilateral e infección por Rickettsia typhi.** Beltrán LM, García S, Vallejo AJ, Bernabeu-Wittel M. Int. J. Environ. Res. Public Health 2009, 6, 2526-2533;doi:10.3390/ijerph6102526.
21. Lancet 2006 Apr 1;367 (9516): 1116. **A souvenir from the Canary Islands.** Somasundaram R, Loddenkemper C, Zeitz M, Schneider T.
22. Internist (Berl.) 2007 Apr;48(4):413-9. **Fever of intermediate duration after return from the Canary Islands.** Basrai D, Pox C, Schmiegel W.
23. Emerging Infectious Diseases • www.cdc.gov/eid • Vol. 11, No. 12, December 2005. **Human Rickettsia felis Infection, Canary Islands, Spain.** Jose-Luis Pérez-Arellano, Florence Fenollar,Alfonso Angel-Moreno, Margarita Bolaños, Michele Hernández, Evora Santana, Marion Hemmersbach-Miller, Antonio-M Martín, and Didier Raoul
24. Oteo JA, Portillo A, Santibañez S, Blanco JR, Pérez L, Ibarra V. Human **Rickettsia felis infections diagnosed by PCR in Spain.** J Clin Microbiol. 2006;44:2669–71.

Este documento incorpora firma electrónica, y es copia auténtica de un documento electrónico archivado por la ULL según la Ley 39/2015.
Su autenticidad puede ser contrastada en la siguiente dirección https://sede.ull.es/validacion/

Identificador del documento: 2483783 Código de verificación: SSH97Xun

Firmado por: Ernesto Pereda de Pablo Fecha: 20/05/2020 13:18:35
UNIVERSIDAD DE LA LAGUNA

En la dirección https://sede.gobcan.es/apjs/verifica_doc puede ser comprobada la autenticidad de esta copia, mediante el número de documento electrónico siguiente:
06hRIWtMLCkqSjtjsILfyf7WU3xVpNOuV

25. Eur J Clin Microbiol Infect Dis. 2006 Jun;25(6):375-81.**Seroepidemiological study of Rickettsia felis, Rickettsia typhi, and Rickettsia conorii infection among the population of southern Spain.** Bernabeu-Wittel M(1), del Toro MD, Nogueras MM, Muniain MA, Cardeñosa N, Márquez. FJ, Segura F, Pachón J.Department of Infectious Diseases, Hospitales Universitarios Virgen del Rocío. Spain.

26. Am. J. Trop. Med. Hyg. 74(1), 2006, pp. 123-126. **Short report: serological evidence of infection with Rickettsia typhi and Rickettsia felis among the human population of Catalonia, in the northeast of spain.** Nogueras M, Cardeñosa N, Sanfeliu I, Muñóz T, Font B, Segura F. Infectious Diseases Program. Department of Internal Medicine, Corporació Sanitaria Parc Taulí, Sabadell, Barcelona, Spain.

27. Oteo JA, Brouqui P. **Ehrlichiosis y anaplasmosis humana.** Enferm Infecc Microbiol Clin. 2005;23:375–80.

28. Jado I, Oteo JA, Aldamiz M, Gil H, Escudero R, Ibarra V, et al. **Rickettsia monacensis.** A new pathogen causing human disease. Emerg Infect Dis. 2007;13:1405–7.

29. Aguirrebengoa K, Portillo A, Santibañez S, Marín JJ, Montejo M, Oteo JA. **First human Rickettsia sibirica mongolitimonae infection in Spain**. Emerg Infect Dis. 2008;14:528–9.

30. Oteo JA, Ibarra V, Blanco JR, Martínez de Artola V, Márquez FJ, Portillo A, et al. ***Dermacentor*-borne necrosis erythema and lymphadenopathy: clinical and epidemiological features of a new tickborne disease.** Clin Microbiol Infect. 2004;10:327–31.

31. Clin Microbiol Infect. 2009 Dec;15 Suppl 2:6-7. doi: 10.1111/j.1469-0691.2008.02729.x. Epub 2009 Apr 3.**Anaplasma phagocytophilum is not an aetiological agent of fever of intermediate duration in Gran Canaria (Spain).** Bolaños M(1), Santana E, Carranza C, Anda P, Jado I, Hernández-Cabrera M, Martín-Sánchez AM, Pérez-Arellano JL.

32. Sánchez-Tejero E, García-Sánchez E. **Empirical treatment with doxycycline for fever of intermediate duration?.**Enferm Infecc Microbiol Clin. 2004 Jun-Jul;22(6):365; author reply 365-6.

Este documento incorpora firma electrónica, y es copia auténtica de un documento electrónico archivado por la ULL según la Ley 39/2015.
Su autenticidad puede ser contrastada en la siguiente dirección https://sede.ull.es/validacion/

Identificador del documento: 2483783 Código de verificación: SSH97Xun

Firmado por: Ernesto Pereda de Pablo *UNIVERSIDAD DE LA LAGUNA* Fecha: 20/05/2020 13:18:35

En la dirección https://sede.gobcan.es/apjs/verifica_doc puede ser comprobada la autenticidad de esta copia, mediante el número de documento electrónico siguiente:
06hRIWtMLCkqSjtjsILfyf7WU3xVpNOuV

33. Instituto Nacional de Estadística. Cifras de población oficial resultante del Padrón municipal en la Isla de la Palma [internet]: 2017. [consultado 09 Feb 2019]. Disponible en: https://www3.gobiernodecanarias.org/istac/statistical-visualizer/visualizer/data.html?resourceType=indicator&resourceId=POBLACION&measure=ABSOLUTE&geo=ES70#visualization/table

34. Wikipedia. The free Encyclopedia. Canary Islands [internet]:2001. [consultado 10 Ag 2018]. Disponible en: https://en.wikipedia.org/w/index.php?title=Canary_Islands&oldid=767377423

35. Aránzazu Portillo and José A. Oteo. **Rickettsiosis as Threat for Traveller.** Cap 1. Current Topics in Tropical Medicina. Edited by Dr. Alfonso Rodríguez-Morales. 2012. www.intechopen.com

36. Tesis Doctoral presentada por Dña. Margarita Bolaños Rivero. Dirigida por los Profesores Dr. Antonio Manuel Martín Sánchez, Dra. O. Évora Santana Rodríguez y Dr. José Luis Pérez Arellano. **Epidemiología, Agentes causales y Métodos diagnósticos en pacientes con Fiebre de Duración Intermedia en Gran Canaria**. 23 de Nov. 2015

37. Oteo el al. Journal of Clinical Microbiology, July 2006, p. 2669–2671

38. Jado et al. Journal of Clinical Microbiology, Dec. 2006, p. 4572–4576

39. Yanes et al., Microb Ecol (2018) 75:264–27

40. M. Berri, K. Laroucau, A. Rodolakis**. The detection of *Coxiella burnetii* from ovine genital swabs, milk and fecal samples by the use of a single touchdown polymerase chain reaction**. Vet Microbiol., 72 (2000), pp. 285-329

Este documento incorpora firma electrónica, y es copia auténtica de un documento electrónico archivado por la ULL según la Ley 39/2015.
Su autenticidad puede ser contrastada en la siguiente dirección https://sede.ull.es/validacion/

Identificador del documento: 2483783 Código de verificación: SSH97Xun

Firmado por: Ernesto Pereda de Pablo Fecha: 20/05/2020 13:18:35
UNIVERSIDAD DE LA LAGUNA

En la dirección https://sede.gobcan.es/apjs/verifica_doc puede ser comprobada la autenticidad de esta copia, mediante el número de documento electrónico siguiente:
06hRIWtMLCkqSjtjsILfyf7WU3xVpNOuV

ANEXO II

I.- RESPONSABLES DE LA EJECUCIÓN DEL PROYECTO Y FORMA DE CONTACTO.

El responsable del Proyecto por parte de la Universitat de Barcelona será el Dr. Carlos Ascaso Terren, con DNI núm. 18158013L, profesor titular del Departament de Fonaments Clínics, de la Facultat de Medicina i Ciències de la Salut, campus Clínic, en calidad de director del Proyecto.

El responsable del Proyecto por parte de la Universidad de la Laguna será D./Dña. Emma Carmelo Pascual, con DNI núm. 42859474D, en calidad de codirectora del Proyecto.

La responsable del Proyecto por parte del Área de Salud de La Palma será D./Dña Mónica Vélez Tobarias, con DNI núm.40347791H.

La responsable del Proyecto por parte del Área de Salud de El Hierro será D./Dña Ana Maria Torres Vega, con DNI núm. 78498830Z.

Todo aviso, solicitud o comunicación que las Partes deban dirigirse en virtud del presente convenio, se efectuará a las siguientes direcciones:

Comunicaciones de carácter científico-técnico:

UNIVERSIDAD DE LA LAGUNA	HOSPITAL GENERAL DE LA PALMA y El HIERRO	UNIVERSITAT DE BARCELONA
Instituto Universitario de Enfermedades Tropicales y Salud Pública de Canarias (IUETSPC)	Servicio de Medicina Interna.	Departament de Fonaments Clínics<br><br>(campus Clínic)
A/A: Emma Carmelo Pascual	A/A: Mónica Vélez Tobarias (HGLP)<br><br>A/A: Ana Mª Torres Vega (HINSR)	A/A: Dr. Carlos Ascaso Terren
Avda Astrofísico Fco. Sánchez s/n.	HGLP: Ctra de la Cumbre, 28<br><br>HINSR: Calle Barriales, 1	Casanova, 143<br><br>08036 Barcelona
Email: ecarmelo@ull.edu.es	Email: mveltob@gobiernodecanarias.org<br><br>atorveg@gobiernodecanarias.org	Email: carlosascaso@ub.edu

Este documento incorpora firma electrónica, y es copia auténtica de un documento electrónico archivado por la ULL según la Ley 39/2015.
Su autenticidad puede ser contrastada en la siguiente dirección https://sede.ull.es/validacion/

Identificador del documento: 2483783 Código de verificación: SSH97Xun

Firmado por: Ernesto Pereda de Pablo Fecha: 20/05/2020 13:18:35
UNIVERSIDAD DE LA LAGUNA

En la dirección https://sede.gobcan.es/apjs/verifica_doc puede ser comprobada la autenticidad de esta copia, mediante el número de documento electrónico siguiente:
06hRIWtMLCkqSjtjsILfyf7WU3xVpNOuV

Teléfono(s): 922 316502 ext. 6109	Teléfono(s):922185160 (HGLP)<br>922553537 (HINSR)	Teléfono: 934035272

Resto de comunicaciones:

UNIVERSIDAD DE LA LAGUNA	SERVICIO CANARIO DE SALUD	UNIVERSITAT DE BARCELONA
Oficina de Transferencia de Resultados de Investigación (OTRI)	Coordinadora de Investigación	Oficina de Recerca (campus Clínic)
A/A	A/A: Dra. Carmen Mora	A/A: Àfrica Cabecerán
C/ Delgado Barreto, s/n. Edificio Central. 38200, La Laguna, Tenerife, España	Dirección del Servicio Canario de la Salud	Casanova, 143<br>08036 Barcelona
Email: otri@ull.edu.es	Email: carmenmora.fdez@gmail.com<br>mmorfers@gobiernodecanarias.org	Email: ofirecerca.medicina@ub.edu
Teléfono: 922316502 (ext. 6437)	Teléfono(s): 922600566<br>679731140	Teléfono: 934039320

II.- COMPONENTES DEL EQUIPO DE TRABAJO

Por parte, de la Universidad de La Laguna, además de la Responsable del Proyecto, formará parte del equipo investigador D. José Antonio Pérez Pérez, Profesor Titular del Área de Genética

Por parte del **Servicio Canario de Salud**, formará parte del equipo de trabajo, el personal de este organismo que determinen en cada momento las personas responsables del Proyecto por el Área de Saldud de La Palma y de EL Hierro, a quienes corresponderá mantener actualizado el correspondiente listado.

Por parte de la **Universitat de Barcelona**,formará parte del equipo de trabajo, el personal que en cada momento las personas que determine en cada momento el responsable del Proyecto, a quien corresponderá mantener actualizado el correspondiente listado.

En caso de que, en aras del buen desarrollo del Proyecto se considere necesaria la modificación del equipo de trabajo, ha de comunicarse a la Comesión de Seguimiento del Convenio.

Este documento incorpora firma electrónica, y es copia auténtica de un documento electrónico archivado por la ULL según la Ley 39/2015.
Su autenticidad puede ser contrastada en la siguiente dirección https://sede.ull.es/validacion/

Identificador del documento: 2483783 Código de verificación: SSH97Xun

Firmado por: Ernesto Pereda de Pablo Fecha: 20/05/2020 13:18:35
UNIVERSIDAD DE LA LAGUNA

En la dirección https://sede.gobcan.es/apjs/verifica_doc puede ser comprobada la autenticidad de esta copia, mediante el número de documento electrónico siguiente:
06hRIWtMLCkqSjtjsILfyf7WU3xVpNOuV

ANEXO III

Cláusula informativa en cumplimiento del deber de información y transparencia del tratamiento.

Las partes informarán a los firmantes que actúan en nombre y representación de cada una de ellas y a las personas de contacto, que los datos de carácter personal que faciliten en virtud del mismo o aquellos que proporcionen con posterioridad, serán tratados por cada una de las partes con la finalidad de gestionar el mantenimiento, cumplimiento, desarrollo, control y ejecución de lo dispuesto en el presente Convenio, siendo la base de legitimación la relación jurídica de las partes. A tal efecto, se incluyen en este anexo las correspondientes cláusulas informativas.

Sin perjuicio de las obligaciones en materia de publicidad activa y derecho de acceso a la información pública previstas en la Ley 19/2013, de 9 de diciembre, de transparencia, acceso a la información pública y buen gobierno, y en la legislación autonómica, las partes se comprometen a respetar la confidencialidad de la información que se suministren en la ejecución del presente Convenio.

Con respecto al tratamiento de datos personales que pudiera derivar de la ejecución del Presente Acuerdo las partes se obligan expresamente a cumplir el Reglamento (UE) 2016/679 del Parlamento y del Consejo, de 27 de abril de 2016, relativo a la protección de las personas físicas por lo que respecta al tratamiento de datos personales y a la libre circulación de estos datos y por el que se deroga la Directiva 95/46/CE (Reglamento general de protección de datos) y La Ley Orgánica 3/2018, de 5 de diciembre, de protección de datos personales y garantía de los derechos digitales.

La **Universitat de Barcelona** como responsable tratará los datos personales de los firmantes del Contrato y de las personas de contacto en los siguientes términos:

1. El responsable del tratamiento de sus datos personales es la Secretaría General de la Universidad de Barcelona, con dirección postal Gran Vía de las Cortes Catalanas, 585, 08007 Barcelona y dirección de correo electrónico secretaria.general@ub.edu.

2. La finalidad del tratamiento de los datos personales es gestionar la ejecución del convenio.

3. La base jurídica para el tratamiento de sus datos personales es la ejecución de un contrato.

4. Sus datos personales se conservarán durante el tiempo necesario para cumplir la finalidad para la que fueron recogidos y para determinar las posibles responsabilidades que se pudieran derivar.

5. La destinataria de los datos es la propia universidad y, en su defecto, los encargados de tratamiento. No se contempla la cesión de datos a terceros, salvo que sea obligación legal. En este supuesto, únicamente se enviarán los datos necesarios.

6. Puede acceder a sus datos, solicitar su rectificación, supresión, la oposición, la portabilidad o la limitación, mediante escrito dirigido a la Secretaría General de la Universidad de Barcelona por correo postal (Gran Vía las Cortes Catalanas, 585, 08007 Barcelona), o por correo electrónico (secretaria.general@ub.edu). Hay que adjuntar una fotocopia del DNI o de otro documento válido que lo identifique.

7. Si considera que sus derechos no se han atendido adecuadamente puede comunicarlo al Delegado de Protección de Datos de la UB por correo postal (Travessera de les Corts, 131-159, Pabellón Rosa, 08028 Barcelona), o por correo electrónico (protecciodedades@ub.edu).

8. También tiene derecho a presentar una reclamación ante la Autoridad Catalana de Protección de Datos.

El **Servicio Canario de la Salud** como responsable tratará los datos personales de los firmantes del Contrato y de las personas de contactoen los siguientes términos:

a) Tratamiento: Gestión de Expedientes Varios del Servicio de Normativa y Estudios

b) Responsable del tratamiento: Secretaría General del Servicio Canario de la Salud

Este documento incorpora firma electrónica, y es copia auténtica de un documento electrónico archivado por la ULL según la Ley 39/2015.
Su autenticidad puede ser contrastada en la siguiente dirección https://sede.ull.es/validacion/

Identificador del documento: 2483783 Código de verificación: SSH97Xun

Firmado por: Ernesto Pereda de Pablo
UNIVERSIDAD DE LA LAGUNA
Fecha: 20/05/2020 13:18:35

En la dirección https://sede.gobcan.es/apjs/verifica_doc puede ser comprobada la autenticidad de esta copia, mediante el número de documento electrónico siguiente:
06hRIWtMLCkqSjtjsILfyf7WU3xVpNOuV

c) Finalidad del tratamiento: La gestión de los Gestión de los expedientes tramitados por el Servicio de normativa y Estudios, con excepción de los expedientes de Responsabilidad Patrimonial.

d) Derechos de las personas interesadas: Acceder, rectificar, opnerse al tratamiento y suprimir los datos, así como otros derechos, como se explica en la información adicionaL.

Información adicional: http://www.gobiernodecanarias.org/cpj/dgmcs/temas/proteccion/tratamientos/cs/scs/sg/normativa-y-estudios/

La **Universidad de La Laguna** como responsable tratará los datos personales de los firmantes del Convenio y de las personas de contacto en los siguientes términos:

a) Responsable

Identidad: UNIVERSIDAD DE LA LAGUNA – Q3818001D

Dirección: Calle Padre Herrera S/n (apartado Postal 456) 38200 – La Laguna

Teléfono: 922319000

Web: www.ull.es

b) Datos de contacto del delegado de protección de datos

Puede contactarse con el delegado de protección de datos mediante correo electrónico remitido a la cuenta dpd@ull.es o mediante escrito dirigido a:

DELEGADO DE PROTECCIÓN DE DATOS

Pabellón de Gobierno, C/ Padre Herrera s/n.

Apartado Postal 456, 38200.

San Cristóbal de La Laguna. S/C de Tenerife.

c) Fines y base jurídica del tratamiento

Los datos personales se tratan con motivo de la ejecución de un contrato al amparo de las previsiones del artículo 6.1.b) del Reglamento General de Protección de Datos.

d) Destinatarios

El Convenio, con indicación del nombre y apellidos de los firmantes puede ser objeto de publicación en el Portal de Transparencia de la ULL.

e) No se prevén transferencias internacionales de datos

f) Criterios de conservación

Los datos personales mantendrán durante la vigencia del contrato. No obstante, serán objeto de conservación posterior en aplicación de la legislación española sobre patrimonio histórico y documental.

g) Ejercicio de derechos

Las personas interesadas pueden ejercer sobre sus datos personales los derechos de acceso, rectificación, supresión y limitación al tratamiento, y a la portabilidad de sus datos personales, a la oposición a su tratamiento, ante la Universidad de La Laguna a través del procedimiento electrónico habilitado al efecto y disponible en la Sede electrónica de la ULL en el siguiente enlace:

https://sede.ull.es/ecivilis-site/catalog/showProcedure/230

e) Autoridad de protección de datos

Las personas interesadas tienen el derecho a presentar reclamaciones ante la Agencia Española de Protección de Datos.

Este documento incorpora firma electrónica, y es copia auténtica de un documento electrónico archivado por la ULL según la Ley 39/2015.
Su autenticidad puede ser contrastada en la siguiente dirección https://sede.ull.es/validacion/

Identificador del documento: 2483783 Código de verificación: SSH97Xun

Firmado por: Ernesto Pereda de Pablo Fecha: 20/05/2020 13:18:35
UNIVERSIDAD DE LA LAGUNA

En la dirección https://sede.gobcan.es/apjs/verifica_doc puede ser comprobada la autenticidad de esta copia, mediante el número de documento electrónico siguiente:
06hRIWtMLCkqSjtjsILfyf7WU3xVpNOuV

ANEXO IV

ENCARGO DE TRATAMIENTO DE DATOS PERSONALES ENTRE EL SERVICIO CANARIO DE LA SALUD (SCS) Y LA UNIVERSIDAD DE LA LAGUNA (ULL)

I. Objeto del encargo del tratamiento

El Servicio Canario de la Salud habilita a la Universidad de La Laguna, mediante las presentes cláusulas, para tratar como encargada del tratamiento, por cuenta de la Dirección del Servicio Canario de la Salud, responsable del tratamiento, los datos personales necesarios para la ejecución del proyecto de investigación sobre fiebre de duración intermedia, objeto del convenio de cooperación del que este encargo de tratamiento constituye uno de sus anexos.

II. Identificación de las actividades de tratamientos afectadas

Para la ejecución de las prestaciones derivadas del cumplimiento del objeto de este encargo, la Dirección del Servicio Canario de la Salud, responsable del tratamiento Historia Clínica, autoriza a la Universidad de La Laguna, encargada del tratamiento, para que el personal de la ULL necesario para la correcta ejecución del convenio trate los datos personales de los pacientes por cuenta de este organismo. Las actividades de tratamiento que se autorizan son recogida, registro, conservación y consulta de datos. Las categorías de datos a tratar por el encargado serán: muestras de sangre, resultados analíticos y código resultante de la seudonimización.

A tal fin, el encargado deberá remitir al Servicio Canario de la Salud un listado del personal que tratará los datos, y comunicará puntualmente cualquier alta o baja del mismo que posibilite un adecuado control.

III. Duración del encargo de tratamiento

El presente encargo está ligado al Convenio de Cooperación entre la Universitat de Barcelona, la Universidad de La Laguna y el Servicio Canario de Salud para la ejecución del proyecto de investigación sobre fiebre de duración intermedia, del que forma parte como anexo.

En consecuencia su duración será la misma del convenio, entendiéndose prorrogado el encargo de tratamiento si fuese prorrogado el convenio (cláusula decimoséptima del convenio de cooperación precitado).

Una vez finalice el presente contrato, el encargado del tratamiento procederá al borrado total de los datos existentes en los equipos informáticos por él utilizados. No obstante, el encargado puede conservar una copia, con los datos debidamente bloqueados, mientras puedan derivarse responsabilidades de la ejecución del convenio.

IV. Obligaciones del encargado del tratamiento

El encargado del tratamiento y todo su personal se somete a la normativa de protección de datos, en particular al Reglamento General de Protección de Datos, a las normativas internas del Servicio Canario de la Salud en materia de seguridad y protección de datos, así como a las medidas aplicables del Esquema Nacional de Seguridad y, de forma específica, a las condiciones siguientes que se ajustarán al nivel de seguridad aplicable:

Este documento incorpora firma electrónica, y es copia auténtica de un documento electrónico archivado por la ULL según la Ley 39/2015.
Su autenticidad puede ser contrastada en la siguiente dirección https://sede.ull.es/validacion/

Identificador del documento: 2483783 Código de verificación: SSH97Xun

Firmado por: Ernesto Pereda de Pablo Fecha: 20/05/2020 13:18:35
UNIVERSIDAD DE LA LAGUNA

En la dirección https://sede.gobcan.es/apjs/verifica_doc puede ser comprobada la autenticidad de esta copia, mediante el número de documento electrónico siguiente:
06hRIWtMLCkqSjtjsILfyf7WU3xVpNOuV

a. Utilizar los datos personales objeto de tratamiento, o los que recoja para su inclusión, sólo para la finalidad objeto de este encargo. En ningún caso podrá utilizar los datos para fines propios.

b. Tratar los datos de acuerdo con las instrucciones del responsable del tratamiento. La remisión de

resultados de las pruebas realizadas se remitirán a la persona responsable del proyecto en el Área de

Salud de La Palma, por correo electrónico, mediante archivos encriptados.

Si el encargado del tratamiento considera que alguna de las instrucciones infringe el RGPD o cualquier otra disposición en materia de protección de datos de la Unión o de los Estados miembros, el encargado informará inmediatamente al responsable a través de la cuenta de correo de la Oficina de Seguridad, aepd.scs@gobiernodecanarias.org.

c. Llevar, por escrito, un registro de todas las categorías de actividades de tratamiento efectuadas por cuenta del responsable, que contenga:

1. El nombre y los datos de contacto del encargado o encargados y de cada responsable por cuenta del cual actúe el encargado y, en su caso, del representante del responsable o del encargado y del delegado de protección de datos.

2. Las categorías de tratamientos efectuados por cuenta de cada responsable.

3. Los sistemas de información y comunicación que el encargado utilice para la recogida, almacenamiento, procesamiento y gestión de los datos, así como el correspondiente tratamiento, deberán ubicarse y prestarse dentro del territorio de la Unión Europea

4. Una descripción general de las medidas técnicas y organizativas de seguridad relativas a:

a) La seudoanimización y el cifrado de datos personales.

b) La capacidad de garantizar la confidencialidad, integridad, disponibilidad y resiliencia permanentes de los sistemas y servicios de tratamiento.

c) La capacidad de restaurar la disponibilidad y el acceso a los datos personales de forma rápida, en caso de incidente físico o técnico.

d) El proceso de verificación, evaluación y valoración regulares de la eficacia de las medidas técnicas y organizativas para garantizar la seguridad del tratamiento.

d. No comunicar los datos a terceras personas, salvo que cuente con la autorización expresa del responsable del tratamiento, en los supuestos legalmente admisibles.

El encargado puede comunicar los datos a otros encargados del tratamiento del mismo responsable, de acuerdo con las instrucciones del responsable. En este caso, el responsable identificará, de forma previa y por escrito, la entidad a la que se deben comunicar los datos, los datos a comunicar y las medidas de seguridad a aplicar para proceder a la comunicación.

Los sistemas de información y comunicación que el encargado utilice para la recogida, almacenamiento, procesamiento y gestión de los datos, así como el correspondiente tratamiento, deberán ubicarse y prestarse dentro del territorio de la Unión Europea.

e. Subcontratación. No se autoriza subcontratar ninguna de las prestaciones que formen parte del objeto de este encargo que comporten el tratamiento de datos personales, salvo los servicios auxiliares necesarios para el normal funcionamiento de los servicios del encargado.

Este documento incorpora firma electrónica, y es copia auténtica de un documento electrónico archivado por la ULL según la Ley 39/2015.
Su autenticidad puede ser contrastada en la siguiente dirección https://sede.ull.es/validacion/

Identificador del documento: 2483783 Código de verificación: SSH97Xun

Firmado por: Ernesto Pereda de Pablo Fecha: 20/05/2020 13:18:35
UNIVERSIDAD DE LA LAGUNA

En la dirección https://sede.gobcan.es/apjs/verifica_doc puede ser comprobada la autenticidad de esta copia, mediante el número de documento electrónico siguiente:
06hRIWtMLCkqSjtjsILfyf7WU3xVpNOuV

f. Mantener el deber de secreto respecto a los datos de carácter personal a los que haya tenido acceso en virtud del presente encargo, incluso después de que finalice su objeto.

g. Garantizar que las personas autorizadas para tratar datos personales se comprometan, de forma expresa y por escrito, a respetar la confidencialidad y a cumplir las medidas de seguridad correspondientes, de las que hay que informarles convenientemente.

h. Mantener a disposición del responsable la documentación acreditativa del cumplimiento de la obligación establecida en el apartado anterior.

i. Garantizar la formación necesaria en materia de protección de datos personales de las personas autorizadas para tratar datos personales.

j. Asistir al responsable del tratamiento en la respuesta al ejercicio de los derechos de:

1. Acceso, rectificación, supresión y oposición

2. Limitación del tratamiento

3. Portabilidad de datos

4. A no ser objeto de decisiones individualizadas automatizadas (incluida la elaboración de perfiles)

Cuando las personas afectadas ejerzan los derechos de acceso, rectificación, supresión y oposición, limitación del tratamiento, portabilidad de datos y a no ser objeto de decisiones individualizadas automatizadas, ante el encargado del tratamiento, éste debe comunicarlo por correo electrónico a la dirección aepd.scs@gobiernodecanarias.org. La comunicación debe hacerse de forma inmediata y en ningún caso más allá del día laborable siguiente al de la recepción de la solicitud, juntamente, en su caso, con otras informaciones que puedan ser relevantes para resolver la solicitud.

k. Notificación de violaciones de la seguridad de los datos. El encargado del tratamiento notificará al responsable del tratamiento, sin dilación indebida, y en cualquier caso antes del plazo máximo de 24 horas, desde su conocimiento, a través de la cuenta de la Oficina de Seguridad, aepd.scs@gobiernodecanarias.org, las violaciones de la seguridad de los datos personales a su cargo de las que tenga conocimiento, juntamente con toda la información relevante para la documentación y comunicación de la incidencia.

Si se dispone de ella se facilitará, como mínimo, la información siguiente:

a) Descripción de la naturaleza de la violación de la seguridad de los datos personales, inclusive, cuando sea posible, las categorías y el número aproximado de interesados afectados, y las categorías y el número aproximado de registros de datos personales afectados.

b) El nombre y los datos de contacto del delegado de protección de datos o de otro punto de contacto en el que pueda obtenerse más información.

c) Descripción de las posibles consecuencias de la violación de la seguridad de los datos personales.

d) Descripción de las medidas adoptadas o propuestas para poner remedio a la violación de la seguridad de los datos personales, incluyendo, si procede, las medidas adoptadas para mitigar los posibles efectos negativos.

Este documento incorpora firma electrónica, y es copia auténtica de un documento electrónico archivado por la ULL según la Ley 39/2015.
Su autenticidad puede ser contrastada en la siguiente dirección https://sede.ull.es/validacion/

Identificador del documento: 2483783 Código de verificación: SSH97Xun

Firmado por: Ernesto Pereda de Pablo Fecha: 20/05/2020 13:18:35
UNIVERSIDAD DE LA LAGUNA

En la dirección https://sede.gobcan.es/apjs/verifica_doc puede ser comprobada la autenticidad de esta copia, mediante el número de documento electrónico siguiente:
06hRIWtMLCkqSjtjsILfyf7WU3xVpNOuV

Si no es posible facilitar la información simultáneamente, y en la medida en que no lo sea, la información se facilitará de manera gradual sin dilación indebida.

Corresponde al responsable del tratamiento comunicar las violaciones de la seguridad de los datos a la Autoridad de Protección de Datos.

l. Dar apoyo al responsable del tratamiento en la realización de las evaluaciones de impacto y las consultas previas a la autoridad de control, cuando proceda.

m. Poner disposición del responsable toda la información necesaria para demostrar el cumplimiento de sus obligaciones, así como para la realización de las auditorías o las inspecciones que realicen el responsable u otro auditor autorizado por él.

n. Implantar las medidas de seguridad previstas en el Anexo II (Medidas de seguridad) del Real Decreto 3/2010, de 8 de enero, por el que se regula el Esquema Nacional de Seguridad en el ámbito de la Administración Electrónica:

n.1 Control del acceso físico a los datos

Se denegará cualquier acceso no autorizado a los sistemas de tratamiento de datos que traten y utilicen datos personales.

Medidas: Se protegerán los edificios con sistemas de control de acceso razonables en función del nivel de seguridad y las directrices de autorización de acceso a los mismos correspondientes. Todos los edificios estarán protegidos con medidas de control del acceso. Los bienes, edificios y determinadas áreas concretas deberán contar con medidas de protección adicionales en función de su nivel de seguridad. Entre dichas medidas se incluyen perfiles de acceso especiales, terminales con contraseña numérica, sistemas de videovigilancia y personal de seguridad. Los derechos de acceso para las personas autorizadas se asignarán de forma individual de conformidad con los criterios establecidos. Esta condición también se aplicará al personal externo.

n.2 Control de acceso a los sistemas de datos

No se permitirá al personal no autorizado utilizar los sistemas de tratamiento de datos.

Medidas: Solo se otorgará acceso a los sistemas de tratamiento de datos a los usuarios autentificados sobre la base de directrices de autorización de cada rol usando las siguientes medidas: cifrado de datos, asignación de contraseñas individualizadas de al menos 10 caracteres que caduquen con regularidad y de forma automática, protectores de pantalla protegidos por contraseña para inactividad, sistemas de detección y prevención de intrusiones, software antivirus actualizado con regularidad y filtros de spyware en la red y en los equipos y dispositivos móviles individuales.

n.3 Control de acceso a los datos

Deberá garantizarse que aquellos usuarios autorizados para utilizar un sistema de tratamiento de datos únicamente puedan acceder a los datos según su autorización de acceso y que ningún usuario no autorizado pueda leer, copiar, modificar ni eliminar los datos personales durante su tratamiento, uso o almacenamiento.

Este documento incorpora firma electrónica, y es copia auténtica de un documento electrónico archivado por la ULL según la Ley 39/2015.
Su autenticidad puede ser contrastada en la siguiente dirección https://sede.ull.es/validacion/

Identificador del documento: 2483783 Código de verificación: SSH97Xun

Firmado por: Ernesto Pereda de Pablo Fecha: 20/05/2020 13:18:35
UNIVERSIDAD DE LA LAGUNA

En la dirección https://sede.gobcan.es/apjs/verifica_doc puede ser comprobada la autenticidad de esta copia, mediante el número de documento electrónico siguiente:
06hRIWtMLCkqSjtjsILfyf7WU3xVpNOuV

Medidas: El acceso a los datos personales se concede sobre la base de directrices de autorización específicas de cada rol. Además, se evitará el acceso no autorizado a los datos personales por medio del cifrado adecuado de los datos. Se configurará la administración de usuarios a fin de asignar el acceso o la salida de los usuarios a las autorizaciones correspondientes. Los cambios quedan registrados en un historial.

n.4 Control de divulgación de datos

Debe garantizarse que los datos personales no se puedan leer, copiar, cambiar ni eliminar sin autorización al transferirlos de forma electrónica ni durante su transporte o almacenamiento en un soporte de datos. Asimismo, la ubicación a la que se vayan a transferir los datos mediante equipos de transmisión de datos deberá poder verificarse y determinarse.

Medidas: Protección de los canales de transmisión electrónica por medio de equipos, redes cerradas y procedimientos para el cifrado de datos. Si el soporte de datos es un transportador físico, disponibilidad de procesos de transporte verificables a fin de proteger los datos ante posibles accesos no autorizados o pérdidas. Los soportes de datos se destruyen de conformidad con la ley de protección de datos

n.5 Control de entrada de datos

Debe garantizarse que posteriormente se pueda comprobar y determinar si se han introducidos datos personales, así como la identidad del usuario que los haya introducido, modificado o eliminado en el sistema de tratamiento de datos.

Medidas: Únicamente se permitirá a los usuarios autentificados el acceso a los datos personales sobre la base de directrices de autorización específicas para cada rol. El acceso a los datos personales, así como la creación, modificación o eliminación de los mismos, quedará registrada debidamente en los archivos de registro.

n.6 Control de orden de datos

Los datos personales que sean accedidos por un tercero en virtud de un encargo de tratamiento se tratarán únicamente conforme a las instrucciones documentadas del SCS.

Medidas: El SCS debe documentar las instrucciones dadas a los contratistas con acceso a datos de carácter personal.

n.7 Control de disponibilidad de datos

Se debe garantizar que los datos personales se encuentren protegidos contra la destrucción accidental o la pérdida de información.

Medidas: Si se almacenan los datos personales, se deberán proteger en sistemas redundantes de recuperación de conformidad con su estado de desarrollo y en función de su nivel de seguridad. Asimismo, se utilizarán sistemas de alimentación ininterrumpida (como SAI, UPS, baterías o generadores) a fin de proteger la fuente de alimentación del centro de datos. Igualmente, deberán proporcionarse directrices exhaustivas por escrito para situaciones de emergencia. También se efectuarán pruebas con regularidad de los procesos y sistemas habilitados para las situaciones de emergencia. Debe contarse con firewalls y otras técnicas de seguridad de red. Asimismo, se dispondrá de software antivirus y filtros de spyware actualizados con regularidad en la red y en todos los sistemas de tratamiento de datos.

n.8 Control de separación de datos

Debe garantizarse que todos los datos tratados para fines distintos se puedan tratar por separado.

Este documento incorpora firma electrónica, y es copia auténtica de un documento electrónico archivado por la ULL según la Ley 39/2015.
Su autenticidad puede ser contrastada en la siguiente dirección https://sede.ull.es/validacion/

Identificador del documento: 2483783   Código de verificación: SSH97Xun

Firmado por: Ernesto Pereda de Pablo   Fecha: 20/05/2020 13:18:35
UNIVERSIDAD DE LA LAGUNA

En la dirección https://sede.gobcan.es/apjs/verifica_doc puede ser comprobada la autenticidad de esta copia, mediante el número de documento electrónico siguiente:
06hRIWtMLCkqSjtjsILfyf7WU3xVpNOuV

Medidas: El SCS debe garantizar que los datos personales se traten de forma tan independiente como sea posible a través de directrices de autorización específicas de cada rol que regulen el acceso a los mismos. De igual manera, deberán separarse los datos de los afectados tanto en los equipos físicos como en los programas informáticos. Los datos personales deberán estar adecuadamente cifrados cuando se almacenen. Los sistemas de pruebas y los sistemas productivos deberán estar separados en unidades lógicas independientes.

En todo caso, deberá implantar mecanismos para:

a) Garantizar la confidencialidad, integridad, disponibilidad y resiliencia permanentes de los sistemas y servicios de tratamiento.

b) Restaurar la disponibilidad y el acceso a los datos personales de forma rápida, en caso de incidente físico o técnico.

c) Verificar, evaluar y valorar, de forma regular, la eficacia de las medidas técnicas y organizativas implantadas para garantizar la seguridad del tratamiento.

d) Seudonimizar y cifrar los datos personales, en su caso.

o. Designar un delegado de protección de datos y comunicar su identidad y datos de contacto al responsable.

p. Destino de los datos. Devolver al encargado que designe por escrito el responsable del tratamiento, los datos de carácter personal y, si procede, los soportes donde consten, una vez cumplida prestación.

La devolución debe comportar el borrado total de los datos existentes en los equipos informáticos utilizados por el encargado.

No obstante, el encargado puede conservar una copia, con los datos debidamente bloqueados, mientras puedan derivarse responsabilidades de la ejecución de la prestación.

V. Obligaciones del responsable del tratamiento

Corresponde al responsable del tratamiento:

a) Entregar al encargado los datos a los que se refiere la cláusula II de este documento. En la entrega de muestras quedará garantizada la seguridad del proceso de remisión.

b) Realizar una evaluación del impacto en la protección de datos personales de las operaciones de tratamiento a realizar por el encargado.

c) Realizar las consultas previas que corresponda.

d) Velar, de forma previa y durante todo el tratamiento, por el cumplimiento del RGPD por parte del encargado.

e) Supervisar el tratamiento, incluida la realización de inspecciones y auditorías.

En prueba de cuanto antecede y en cumplimiento de lo dispuesto en el artículo 28 del RGPD, las partes firman el presente contrato en la fecha que figura en la firma electrónica.

Este documento incorpora firma electrónica, y es copia auténtica de un documento electrónico archivado por la ULL según la Ley 39/2015.
Su autenticidad puede ser contrastada en la siguiente dirección https://sede.ull.es/validacion/

Identificador del documento: 2483783 Código de verificación: SSH97Xun

Firmado por: Ernesto Pereda de Pablo
UNIVERSIDAD DE LA LAGUNA
Fecha: 20/05/2020 13:18:35

Firmado digitalmente por JUAN ELIAS (R:Q0818001J)
Fecha: 2020.05.27 10:54:12 +02'00'

Este documento ha sido firmado electrónicamente por:
ANTONIO JOSE OLIVERA HERRERA - VICECONSEJERO DE LA PRESIDENCIA
Fecha: 28/05/2020 - 18:46:03

En la dirección https://sede.gobcan.es/apjs/verifica_doc puede ser comprobada la autenticidad de esta copia, mediante el número de documento electrónico siguiente:
06hRIWtMLCkqSjtjsILfyf7WU3xVpNOuV

El presente documento ha sido descargado el 01/06/2020 - 09:27:50
