# Supplementary material for: Murine typhus as the leading cause of non-focalized fever in the Canary Islands
Source: Eur J Clin Microbiol Infect Dis. 2024 Nov 29;44(2):323–32. doi: 10.1007/s10096-024-04976-8 (PMC11754304; doi:10.1007/s10096-024-04976-8)
Supplement: Supplementary file 2 — Supplementary file2 (PDF 53 KB) [file 10096_2024_4976_MOESM2_ESM.pdf]

Servicio Canario de la Salud

Gobierno de Canarias

El estudio de investigación titulado: "**Fiebre de Duración Intermedia en la isla de La Palma y la isla de El Hierro**", versión **2, del 22 de febrero de 2019,** con **código 2017_81**, del que son Investigadores Principales la Dra. MONICA VELEZ TOBARIAS y la Dra. ANA Mª TORRES VEGA, ha sido evaluado por el Comité de Ética de la Investigación con medicamentos del Complejo Hospitalario Universitario de Canarias (Provincia de Santa Cruz de Tenerife) en su sesión del **11/04/2019**, y considera que:

Se cumplen los requisitos necesarios de idoneidad del Protocolo con los objetivos del estudio.

El procedimiento para obtener el consentimiento informado, incluyendo la hoja de información para los sujetos y el consentimiento informado**, versión 2, del 22 de febrero de 2019,** es adecuado.

La capacidad del Investigador y los medios disponibles son adecuados para llevar a cabo el estudio y no interfiere con el respeto a los postulados éticos.

Por todo ello, el Comité de Ética de la Investigación con medicamentos del Complejo Hospitalario Universitario de Canarias (Provincia de Santa Cruz de Tenerife) emite dictamen **FAVORABLE** para la realización de este estudio en el Hospital General de La Palma (HGLP) y en el Hospital Insular Ntra. Sra. de los Reyes (HINSR).

Secretaria Técnica del CEIm
Complejo Hospitalario Universitario de Canarias

Este documento ha sido firmado electrónicamente por:	
CONSUELO MARIA RODRIGUEZ JIMENEZ - F.E.A. FARMACOLOGIA CLINICA	Fecha: 25/04/2019 - 14:21:38
En la dirección https://sede.gobcan.es/sede/verifica_doc puede ser comprobada la autenticidad de esta copia, mediante el número de documento electrónico siguiente: 0U2Zjn7d4Qkfh3YA2IaCz71uV5mqeK5q3	
El presente documento ha sido descargado el 25/04/2019 - 14:36:12	
