# Supplementary material for: Murine typhus as the leading cause of non-focalized fever in the Canary Islands
Source: Eur J Clin Microbiol Infect Dis. 2024 Nov 29;44(2):323–32. doi: 10.1007/s10096-024-04976-8 (PMC11754304; doi:10.1007/s10096-024-04976-8)
Supplement: Supplementary file 5 — Supplementary file5 (PDF 248 KB) [file 10096_2024_4976_MOESM5_ESM.pdf]

HOJA DE INFORMACIÓN AL PACIENTE PARA MENORES DE 18 AÑOS

TÍTULO DEL ESTUDIO:
Fiebre de Duración Intermedia en la isla de La Palma y la isla de El Hierro
Versión 2 del 22 de febrero de 2019

INVESTIGADOR PRINCIPAL LOCAL: Mónica Vélez Tobarias – Médico Interna- HGLP – 922185160.
Ana Mª Torres Vega – Médico Internista – HINSR - 922553537

CENTRO:

INTRODUCCION

Nos dirigimos a usted para informarle sobre un estudio de investigación en el que se le invita a participar. El estudio ha sido aprobado por el Comité de Ética de la Investigación correspondiente.

Nuestra intención es tan solo que usted reciba la información correcta y suficiente para que pueda evaluar y juzgar si quiere o no participar en este estudio. Para ello lea esta hoja informativa con atención y nosotros le aclararemos las dudas que le puedan surgir después de la explicación. Además, puede consultar con las personas que considere oportuno.

PARTICIPACIÓN VOLUNTARIA

Debe saber que su participación en este estudio es voluntaria y que puede decidir no participar o cambiar su decisión y retirar el consentimiento en cualquier momento, sin que por ello se altere la relación con su médico ni se produzca perjuicio alguno en su tratamiento.

DESCRIPCIÓN GENERAL DEL ESTUDIO:

La fiebre de duración intermedia (FDI) se define como fiebre mayor de 38ºC de 7 a 28 días de duración que permanece sin diagnóstico a pesar de una correcta evaluación médica y, habiendo incluido las pruebas complementarias de rutina (radiografía de tórax, orina y analítica básica).
La causa de FDI más frecuente conocida hasta el momento, es la fiebre Q, sin embargo, hay otras enfermedades que pueden producir un cuadro clínico similar, algunas de ellas no conocidas y otras poco conocidas pero, con casos demostrados en las islas Canarias como el tifus murino.
El estudio presente pretende incluir a todos los pacientes que presenten fiebre compatible con FDI en un protocolo dirigido que incluye extracción sanguínea y un cuestionario clínico inicial con el correspondiente seguimiento habitual por parte de su Médico de Atención Primaria y, en caso de necesidad, por parte del médico especialista en Medicina Interna.
Este protocolo pretende lograr un tratamiento más rápido y eficaz, proporcionando mayor beneficio para los pacientes con FDI y, evitando los inconvenientes de su desconocimiento.
Su colaboración en este estudio contribuirá a una mayor aportación científica tanto a nivel local, nacional como internacional para mejorar el conocimiento de las causas de FDI, su mejor manejo clínico y la posible mejoría de su prevención en el futuro.

OBTENCIÓN DE MUESTRAS BIOLÓGICAS:

Con el fin de colaborar en este aspecto del estudio, se precisa una muestra biológica de sangre. En el servicio rutinario de extracción de muestras correspondiente a su Centro de Salud, se le realizará la extracción sanguínea habitual para estudio de FDI mediante proceso de venopunción. En el mismo proceso, se obtendrá además, 2 ml de sangre extra en un tubo con EDTA que se mandará al Instituto Universitario de Enfermedades Tropicales y Salud Pública de Canarias en la isla de Tenerife (IUETSPC) para estudio de PCR. Este procedimiento implicará los riesgos propios de la extracción sanguínea habitual, es decir, posibilidad de dolor con la venopunción, riesgo de extravasación sanguínea, etc.
Estas pruebas no sustituirán al estudio diagnóstico habitual mediante serología ya que por el momento sólo son parte de un proyecto de investigación y usted tiene total libertad para participar.
Además le garantizamos que:
1. El ADN será utilizado exclusivamente para los fines del estudio, es decir, el estudio de las diferentes causas de FDI. No podrá ser utilizado para ningún otro estudio sin su autorización expresa, y quedará custodiado en el IUETSPC.
2. La muestra será custodiada con un código interno que no permite por sí mismo identificarle. Las personas que tienen acceso a ese código son los investigadores principales del proyecto, la procesa y la registra en una aplicación informática. Esta aplicación cumple la normativa vigente de confidencialidad de la información.
3. Si usted lo solicita, sus muestras pueden ser destruidas en cualquier momento del proceso. En ese caso se le informará adecuadamente y en su momento de la fecha de destrucción del material.
4. La información que se derive del análisis es confidencial, y no podrá ser utilizada con otros fines diferentes a los del estudio.
5. Por las características de este estudio no se espera un resultado inesperado que pueda repercutir sobre su esperanza y calidad de vida, o la de sus familiares. En caso de que ocurriera en un futuro, le notificaríamos tal posibilidad para que usted decida si desea recibir o no la información. Es conveniente por tanto, que usted transmita esta posibilidad a sus familiares antes de participar en el estudio.
6. Bajo ningún concepto y en ningún momento las muestras serán motivo de lucro directo o transacción comercial, bien sea por la venta del material o de los derechos para realizar estudios sobre los mismos.

CONFIDENCIALIDAD

El tratamiento, la comunicación y la cesión de los datos de carácter personal de todos los sujetos participantes se ajustará a lo dispuesto en la Ley Orgánica 3/2018, de 5 de diciembre de Protección de Datos Personales y garantía de los derechos digitales, y a la aplicación de del Reglamento (UE) 2016/679 del Parlamento europeo y del Consejo de 27 de abril de 2016 de Protección de Datos (RGPD), por lo que es importante que conozca la siguiente información:

- Además de los derechos que ya conoce (acceso, modificación, oposición y cancelación de datos) ahora también puede limitar el tratamiento de datos que sean incorrectos, solicitar una copia o que se trasladen a un tercero (portabilidad) los datos que usted. ha facilitado para el estudio. Para ejercitar sus derechos, diríjase al investigador principal del estudio. Le recordamos que los datos no se pueden eliminar aunque deje de participar en el estudio para garantizar la validez de la investigación y cumplir con los deberes legales y los requisitos de

autorización de medicamentos. Así mismo tiene derecho a dirigirse a la Agencia de Protección de Datos si no quedara satisfecho/

- Tanto el Centro como el Promotor y el Investigador son responsables respectivamente del tratamiento de sus datos y se comprometen a cumplir con la normativa de protección de datos en vigor. Los datos recogidos para el estudio estarán identificados mediante un código, de manera que no se incluya información que pueda identificarle, y sólo su médico del estudio/colaboradores podrá relacionar dichos datos con usted y con su historia clínica. Por lo tanto, su identidad no será revelada a ninguna otra persona salvo a las autoridades sanitarias, cuando así lo requieran o en casos de urgencia médica. Los Comités de Ética de la Investigación, los representantes de la Autoridad Sanitaria en materia de inspección y el personal autorizado por el Promotor, únicamente podrán acceder para comprobar los datos personales, los procedimientos del estudio clínico y el cumplimiento de las normas de buena práctica clínica (siempre manteniendo la confidencialidad de la información).

El Investigador y el Promotor están obligados a conservar los datos recogidos para el estudio al menos hasta 25 años tras su finalización. Posteriormente, su información personal solo se conservará por el centro para el cuidado de su salud y por el promotor para otros fines de investigación científica si usted hubiera otorgado su consentimiento para ello, y si así lo permite la ley y requisitos éticos aplicables.

INFORMACIÓN ADICIONAL

Tal y como exige la ley, para participar deberá firmar y fechar el documento de consentimiento informado.

El investigador responsable de este estudio en este centro es el Dr……………………
Si durante la realización de este estudio le surge alguna cuestión relacionada con el mismo, puede consultar con el Dr……………………..del Servicio de…….…………
del hospital………….
en el número de teléfono 922.................

Universitat de Barcelona

Servicio Canario de la Salud

Instituto Universitario de Enfermedades Tropicales y Salud Pública de Canarias
Universidad de La Laguna

CONSENTIMIENTO INFORMADO A MENORES DE 18 AÑOS

Paciente (nombre y apellidos)

....

Representante legal (padre- madre o tutor)

Nombre y apellidos: ……………………………………………………………..

DNI: ………………………………………………………………………………

He leído la hoja de información que se me ha entregado.
He podido hacer preguntas sobre el estudio.
He recibido suficiente información sobre el estudio.

He hablado con:

....
(nombre del investigador)

Comprendo que mi participación es voluntaria.
Comprendo que puedo retirarme del estudio:

1º Cuando quiera
2º Sin tener que dar explicaciones.
3º Sin que esto repercuta en mis cuidados médicos.

- Presto libremente mi conformidad para participar en el estudio y doy mi consentimiento para el acceso y utilización de mis datos en las condiciones detalladas en la hoja de información.

Firma del representante legal:
Nombre:
Fecha:

Firma del investigador:
Nombre:
Fecha:
