# Supplementary material for: Murine typhus as the leading cause of non-focalized fever in the Canary Islands
Source: Eur J Clin Microbiol Infect Dis. 2024 Nov 29;44(2):323–32. doi: 10.1007/s10096-024-04976-8 (PMC11754304; doi:10.1007/s10096-024-04976-8)
Supplement: Supplementary file 6 — Supplementary file6 (PDF 573 KB) [file 10096_2024_4976_MOESM6_ESM.pdf]

Título del estudio:

Fiebre de Duración Intermedia en la isla de La Palma y la isla de El Hierro

Plataforma de Investigación en Fiebre de Duración Intermedia entre la Universidad de Barcelona, el *Instituto Universitario de Enfermedades Tropicales y Salud Pública de Canarias* de la Universidad de La Laguna, el Área de Salud de La Palma y de la isla del Hierro.

CÓDIGO: plataformafdi19
VERSIÓN: 2; 16/11/2017

EQUIPO INVESTIGADOR:
Mónica Vélez Tobarias
FEA Medicina Interna. Hospital General de la Palma (HGLP)
Ana Mª Torres Vega
FEA Medicina Interna. Hospital Insular Ntra. Sra. de los Reyes (HINSR)
Dr. Carlos Ascaso Terrén
Departament de Fonaments Clínics, UB - Línea de Investigación, Salud Internacional.
Dra. Emma Carmelo Pascual.
Departamento de Obstetricia y Ginecología, Pediatría, Medicina Preventiva y Salud Pública, Toxicología, Medicina Legal y Forense y Parasitología. Instituto Universitario de Enfermedades Tropicales y Salud Pública de Canarias (IUETSPC). Universidad de la Laguna (ULL).

Este documento incorpora firma electrónica, y es copia auténtica de un documento electrónico archivado por la ULL según la Ley 39/2015.
La autenticidad de este documento puede ser comprobada en la dirección: https://sede.ull.es/validacion/

Identificador del documento: 1764975 Código de verificación: 37vTZjpe

Firmado por: Emma Carmelo Pascual Fecha: 21/02/2019 14:10:51
UNIVERSIDAD DE LA LAGUNA

ÍNDICE

Preguntas de investigación e Introducción 2

Hipótesis 4

Objetivos 5

Materiales y métodos 5

Localización del estudio... 5

Población de estudio 5

Diseño 6

Tamaño de muestra 7

Proceso de reclutamiento 7

Criterios inclusión 8

Criterio exclusión 8

Criterios de hospitalización 9

Seguimiento en Atención Primaria 9

Zoonosis emergentes como potenciales causas de Fiebre de Duración Intermedia y sus características principales 10

Algoritmo diagnóstico y tratamiento 13

Algoritmo proceso reclutamiento 14

Variables de estudio 15

Instrumentos usados en el estudio 16

Análisis estadístico 17

Limitaciones de estudio 17

Convenio de colaboración 17

Aspectos éticos 17

Resultados esperados.. 18

Bibliografía relevante 20

Este documento incorpora firma electrónica, y es copia auténtica de un documento electrónico archivado por la ULL según la Ley 39/2015.
La autenticidad de este documento puede ser comprobada en la dirección: https://sede.ull.es/validacion/

Identificador del documento: 1764975 Código de verificación: 37vTZjpe

Firmado por: Emma Carmelo Pascual Fecha: 21/02/2019 14:10:51
UNIVERSIDAD DE LA LAGUNA

Título del proyecto

Fiebre de Duración Intermedia en la isla de La Palma y la isla del Hierro

1. Preguntas de investigación e Introducción

(1)¿Cuáles son las causas infecciosas de FDI en la isla de La Palma y El Hierro y su incidencia?

(2)¿Cuales son las características diferenciales entre las distintas etiologías infecciosas más frecuentes de FDI?

a. ¿Qué patologías presentan más hospitalización?
b. ¿Existe asociación entre patologías y complicaciones?
c. ¿Existe un perfil de síntomas/signos/laboratorio asociado a cada patología?
d. ¿Existen diferencias de signos/síntomas/laboratorio entre pacientes hospitalizados versus no hospitalizados dentro de cada patología?
e. ¿Existen diferencias de signos/síntomas/laboratorio entre pacientes con complicación versus no complicación dentro de cada patología?

La fiebre de duración intermedia (FDI) se define como fiebre mayor de 38ºC de 7 a 28 días de duración que permanece sin diagnóstico a pesar de una correcta anamnesis, exploración física y pruebas complementarias de rutina, incluyendo, radiografía de tórax, sedimento de orina, hemograma y bioquímica básica. El uso de este término aparece por primera vez en España el año 1992 siendo, posteriormente, su uso extendido a nivel nacional desde 1999 gracias a los trabajos de Bernabeu-Wittel[1-2]. Sin embargo, no se menciona este término a nivel internacional en búsquedas realizadas a través de medline. A pesar de ser una de las causas más frecuentes de fiebre en la práctica médica diaria, este concepto tampoco está incluido en los tratados clásicos de Medicina Interna que sólo distinguen entre fiebre de corta

Este documento incorpora firma electrónica, y es copia auténtica de un documento electrónico archivado por la ULL según la Ley 39/2015.
La autenticidad de este documento puede ser comprobada en la dirección: https://sede.ull.es/validacion/

Identificador del documento: 1764975 Código de verificación: 37vTZjpe

Firmado por: Emma Carmelo Pascual Fecha: 21/02/2019 14:10:51
UNIVERSIDAD DE LA LAGUNA

duración (FCD) o fiebre aguda y, fiebre de origen desconocido (FOD) [4-5]. El trabajo más amplio de FDI realizado por el grupo de Espinosa et al., en Sevilla, muestra un claro predominio de enfermedades infecciosas, entre las más frecuentes la fiebre Q, brucellosis, tifus murino, fiebre botonosa mediterránea y, menos frecuentes, la fiebre tifoidea, síndrome mononucleósico (incluyendo VEB, CMV y toxoplasmosis) y leptospirosis, un 18.8% de casos son de etiología desconocida y otro porcentaje mucho menor de causas no infecciosas[3]. El espectro etiológico de FDI no es estable, varia en el tiempo y en función de la localidad pero se carece de información por la limitación de estudios en las distintas regiones de España. Estudios similares se han realizado en la Rioja[6]. Los agentes etiológicos más frecuentes de FDI pertenecen a la familia Rickettsiae que incluye a los distintos géneros: *Rickettsiae, Ehrlichia, Orienta y Coxiella*[7-8].

No existen estudios que hayan determinado el perfil etiológico de FDI en las islas Canarias aunque si extensa bibliografía sobre fiebre Q [9-15] y, en los últimos años sobre tifus murino[16-20], las cuales figuran entre las causas más frecuentes de FDI. A nivel internacional también se han reportado casos de FDI en viajeros procedentes de las islas Canarias[21-22].

Por otro lado, hay numerosos estudios que describen nuevos agentes emergentes en España causantes de FDI como *Rickettsia felis* [23-26], Ehrlichiosis y anaplasmosis humana[27], *Rickettsia monacensis*[28], *Rickettsia sibirica mongolitimonae*[29], *Rickettsia slovaca*[30]. Los 5 primeros casos de *Rickettsia felis* en las islas Canarias fueron descritos en 2005 por el grupo de Pérez-Arellano[23]. Sin embargo, no se ha podido demostrar presencia de *Anaplasma phagocytophilum*[31] en las islas en humanos. La fiebre Q causada por *Coxiella burnetii* es la causa de FDI más frecuente por lo que se plantea el uso empírico de doxiciclina como tratamiento para esta entidad hasta su diagnóstico serológico definitivo[32].

En la isla de Tenerife hay publicado un estudio clínico-epidemiológico y de características diferenciales de fiebre Q con tifus murino, reportándose 47 y 32 casos, respectivamente, recopilados entre el 1998-2002[17]. Por otro lado, hay publicadas series de casos y casos clínicos aislados referentes a complicaciones clínicas del tifus murino como afectación renal[18], neumonía[19], vasculitis[17] y uveítis anterior[20] en las islas Canarias. Hasta el momento no ha habido estudios que hayan permitido detectar indicadores de riesgo de

Este documento incorpora firma electrónica, y es copia auténtica de un documento electrónico archivado por la ULL según la Ley 39/2015.
La autenticidad de este documento puede ser comprobada en la dirección: https://sede.ull.es/validacion/

Identificador del documento: 1764975 Código de verificación: 37vTZjpe

Firmado por: Emma Carmelo Pascual Fecha: 21/02/2019 14:10:51
UNIVERSIDAD DE LA LAGUNA

hospitalización, complicación ni diferencias clínicas ni laboratoriales de cada entidad nosológica previo a su diagnóstico serológico.

Basándonos en que la FDI es el tipo de fiebre por el que más se consulta en la práctica médica diaria además, de su espectro cambiante en tiempo y espacio y, la aparición continua de nuevos agentes etiológicos, nos motiva a profundizar en su estudio en la isla de la Palma y el Hierro, dos de las islas Canarias, caracterizadas todas ellas por su singularidad geográfica. Esta información nos permitiría consensuar protocolos de manejo diagnóstico-terapéutico que fueran costo-efectivos en las islas e, incluso, orientarnos a una mayor vigilancia epidemiológica y prevención de sus causas ya que, en su mayoría, se trata de zoonosis relacionadas con vectores transmisores en el ambiente.

Los estudios respecto a complicaciones y criterios de hospitalización de estas entidades son muy escasos por lo que identificar predictores de riesgo de ambos influiría directamente en la calidad asistencial de los pacientes y, posiblemente, la disminución de costes sanitarios en el manejo de esta patología.

Todo ello supondría un gran avance científico con potencial impacto en el campo de la Salud Pública para esta entidad nosológica, relativamente joven, definida en España.

Impacto: disminuir los diagnósticos inespecíficos con dificultad de tratamiento; introducir como diagnóstico específico patologías no incluidas en la isla; identificar predictores de riesgo y hospitalización por patología; aumentar los tratamientos específicos y el éxito en el tratamiento; establecer programas preventivos de disminución de incidencia de dichas patologías.

Hipótesis

(1) La fiebre Q y el tifus murino son las causas de FDI con mayor tasa en la isla de La Palma y la isla de El Hierro.
(2) Existe un perfil epidemiológico para la FDI.
(3) Existe un perfil etiológico de causas infecciosas FDI en la isla de La Palma y El Hierro distinto al descrito en otras áreas de España.

Este documento incorpora firma electrónica, y es copia auténtica de un documento electrónico archivado por la ULL según la Ley 39/2015.
La autenticidad de este documento puede ser comprobada en la dirección: https://sede.ull.es/validacion/

Identificador del documento: 1764975 Código de verificación: 37vTZjpe

Firmado por: Emma Carmelo Pascual Fecha: 21/02/2019 14:10:51
UNIVERSIDAD DE LA LAGUNA

(4) Existe un perfil de síntomas/signos/laboratorio distinto para las diferentes causas de FDI.
(5) La tasa de hospitalización y el tipo de complicaciones son distintos según la causa etiológica de la FDI.
(6) Existen diferencias de signos/síntomas/laboratorio de pacientes hospitalizados versus no hospitalizados dentro de cada patología.

Objetivos

(1) Definir un perfil etiológico de causas infecciosas de FDI en la isla de La Palma y El Hierro.
(2) Describir características epidemiológicas de los casos de FDI.
(3) Definir si existe un perfil clínico/laboratorio distintivo de cada patología.
(4) Identificar qué patologías presentan más riesgo de hospitalización y de complicaciones.
(5) Identificar indicadores predictores de riesgo para cada patología.

2. METODOLOGÍA

Localización del estudio:

Canarias forma parte de una de las diecisiete comunidades autónomas de España y, una de las regiones ultraperiféricas de la Unión Europea. El archipiélago canario está conformado por ocho islas de origen volcánico, cinco islotes y ocho roques. Su clima está influenciado por la interacción de corrientes marinas del banco sahariano y los vientos alisios, resultando ser suave y homogéneo a lo largo del año, de tipo subtropical. Canarias ha sido considerada históricamente un puente de conexión entre tres continentes: Europa, África y América.

El estudio se llevará a cabo en dos de las islas Canarias, la isla de La Palma y la isla de El Hierro. La isla de la Palma es la segunda isla más occidental del Archipiélago Canario, de clima subtropical y origen volcánico, situada al norte de África, en el océano Atlántico (Latitud: 28,6 Longitud: -17,7), cerca de las costas del sur de Marruecos y el Sáhara. La distancia mínima a la costa peninsular son de unos 1000 km. Tiene una superficie de 708,33 km² (10% del territorio

Este documento incorpora firma electrónica, y es copia auténtica de un documento electrónico archivado por la ULL según la Ley 39/2015.
La autenticidad de este documento puede ser comprobada en la dirección: https://sede.ull.es/validacion/

Identificador del documento: 1764975 Código de verificación: 37vTZjpe

Firmado por: Emma Carmelo Pascual Fecha: 21/02/2019 14:10:51
UNIVERSIDAD DE LA LAGUNA

canario) y una población de 81.350 habitantes a fecha 1 de enero de 2017[33]. El punto más elevado de la isla es el Roque de Los Muchachos, a una altitud de 2.426 m[34].

La isla se divide en 14 municipios: al norte Garafía, Barlovento, Puntagorda y Los Sauces; al sur Fuencaliente; al este Puntallana, Santa Cruz, Breña Alta y Breña Baja, Mazo; al Oeste Tijarafe, Tazacorte, Los Llanos y en el centro el Paso. El único hospital de la isla, llamado Hospital General de la Palma (HGLP) que, atiende a toda la población, está localizado al este, en Breña Alta.

El Hierro es la isla canaria más pequeña, joven, occidental y meridional. Fue declarada por la UNESCO (*United Nations Educational, Scientific and Cultural Organization*) en el año 2000 Reserva Mundial de la Biosfera y Geoparque en 2014. Posee una gran cantidad de acantilados, costas rocosas y muy abruptas entre las que se ubican numerosas piscinas naturales y charcos. La geografía herreña en general es abrupta, presenta uno de los mayores gradientes de desnivel altitudinal de las islas Canarias, conformando un edificio piramidal de base triangular por asociación de tres dorsales volcánicas. Su punto de máxima altura es el Pico de Malpaso situado a 1501,762 m. Cuenta con una superficie de 268,71 kilómetros cuadrados (Km^2) y, según datos del Instituto Canario de Estadística (ISTAC), una población de 10.798 habitantes en 2017. Sus habitantes se distribuyen en tres municipios: Valverde (4.955 habitantes en 2017), La Frontera (4.018 habitantes en 2018) y El Pinar (1.825 habitantes en 2017) con un elevado nivel de dispersión y una densidad media de 40 hab/Km^2.

El Área de Salud de El Hierro, al cual pertenece el Hospital Insular Ntra. Sra. de los Reyes (HINSR), se compone de 2 Zonas Básicas de Salud, que cubren la totalidad del territorio insular: Zona Especial de Salud de Valverde (comprende los municipios de Valverde y El Pinar) y Zona Especial de Salud de Frontera-Valle del Golfo **(**abarca el municipio de La Frontera).

Población de estudio: toda la población de la isla de La Palma y El Hierro.

Diseño: Se llevará a cabo un estudio descriptivo transversal de los casos diagnosticados de FDI durante 12 meses consecutivos a partir de la fecha de inicio del estudio. Dicho estudio incluirá toda la población de la isla de La Palma y El Hierro.

Este documento incorpora firma electrónica, y es copia auténtica de un documento electrónico archivado por la ULL según la Ley 39/2015.
La autenticidad de este documento puede ser comprobada en la dirección: https://sede.ull.es/validacion/

Identificador del documento: 1764975 Código de verificación: 37vTZjpe

Firmado por: Emma Carmelo Pascual
UNIVERSIDAD DE LA LAGUNA
Fecha: 21/02/2019 14:10:51

Tamaño de la muestra:

Todos los casos con diagnóstico de FDI diagnosticados durante 12 meses consecutivos. Se estima un reclutamiento medio de 10-14 casos al mes y una muestra final de tamaño 120-168 casos. Previamente al inicio de reclutamiento se realizará un proyecto piloto durante 2 semanas para nueva estimación de tamaño muestral ya que el actual esta basado en un estudio retrospectivo de fiebre Q y rickettiosis durante el año 2013.

Proceso de reclutamiento de casos y envío de las muestras:

Para realizar el reclutamiento de los casos se distribuirá un algoritmo de manejo diagnóstico-terapéutico, los criterios de inclusión, exclusión y los criterios de derivación de FDI a todos los Centros de Atención Primaria participantes, especialidades hospitalarias de Medicina Interna y Servicio de Urgencias Hospitalario. Los pacientes que cumplan criterio diagnóstico y criterio de inclusión firmarán un consentimiento informado aceptando la participación en el estudio, la extracción de sangre y rellenar un cuestionario inicial con la anamnesis y datos básicos epidemiológicos. Tras firma de consentimiento informado se solicitará extracción sanguínea de 2 tubos de sangre. Un tubo para estudio de serología y otro para estudio de PCR. El estudio serológico inicial incluirá: *Coxiella burnetii, Rickettsia typhi, Citomegalovirus, virus Epstein-Barr* (estos estudios se incluyen dentro de los rutinarios en el HGLP). En el segundo tubo con EDTA se guardarán 2 ml de sangre para remitir al Instituto Universitario de Enfermedades Tropicales y Salud Pública de Canarias (IUETSPC) en Tenerife para los estudios diagnósticos de PCR que no se incluyen entre los habituales. El proceso de recogida de muestras seguirá el habitual del Área de Salud de la Palma y El Hierro. La muestras serán recogidas en los centros de salud y transportadas al HGLP y al HINSR, respectivamente. Una vez lleguen al hospital, las muestras para serología seguirán el procedimiento habitual y las muestras reservadas para estudio de PCR serán congeladas -30-35º. Según número de muestras acumuladas serán remitidas semanal o bisemanalmente al IUETSPC por los medios de transporte habituales conservándose las muestras con hielo seco y evitando la congelación/descongelación de las mismas. Una vez en el IUETSPC, las muestras serán almacenadas a -80ºC hasta su procesamiento para detección molecular de ADN. Los estudios

Este documento incorpora firma electrónica, y es copia auténtica de un documento electrónico archivado por la ULL según la Ley 39/2015.
La autenticidad de este documento puede ser comprobada en la dirección: https://sede.ull.es/validacion/

Identificador del documento: 1764975 Código de verificación: 37vTZjpe

Firmado por: Emma Carmelo Pascual Fecha: 21/02/2019 14:10:51
UNIVERSIDAD DE LA LAGUNA

de PCR que se realizarán en el IUETSPC incluirán: *Coxiella burnetti, Rickettsia tiphy* y otras rickettsiosis emergentes*: Rickettsia felis, Rickettsia africae, Rickettsia massiliae, Rickettsia monacensis, Bartonella spp, Erlichia spp.* entre otros. Todos aquellos casos que cumplan criterio de FDI podrán recibir tratamiento antibiótico con doxiciclina según criterio médico una vez haya sido extraída la muestra de sangre. Si el resultado de serología y PCR son positivos y concordantes se considerará un caso con diagnóstico positivo. Si el resultado de serología es negativo o no concluyente el paciente será remitido al Servicio de Medicina Interna para ampliar el estudio según sospecha diagnóstica específica y contexto epidemiológico mediante la siguiente serología: VIH, *Parvovirus B19, Brucella mellitensis, Rickettsi conorii, Leptospira, Legionella, Chlamydia, Yersinia spp, Toxoplasma gondii, VHB, test tuberculina, RPR y FTA* (estos estudios se realizarán según las técnicas y procedimientos habituales en el HGLP y HINSR, respectivamente). A los casos con serología positiva para *Coxiella Burnetti, Rickettsia typhi o Rickettsia conorii* se les realizará serología seriada de control a los 14 días según la práctica habitual.

Criterios de inclusión:

Fiebre>38ºC, sin foco aparente de 7 a 28 días de duración.

1) No ingreso hospitalario previo.
2) Sin enfermedad crónica o inmunodeficiencia conocidas.
3) No diagnóstico a pesar de estudios preliminares básicos, incluyendo historia clínica, hemograma, sedimento orina, función renal y radiografía de tórax.
1) Inmigrantes con más de 1 año de residencia.
2) VIH no conocidos
3) Pacientes sin patología oncológica activa o tratamientos oncológicos inmunosupresores.

Este documento incorpora firma electrónica, y es copia auténtica de un documento electrónico archivado por la ULL según la Ley 39/2015.
La autenticidad de este documento puede ser comprobada en la dirección: https://sede.ull.es/validacion/

Identificador del documento: 1764975 Código de verificación: 37vTZjpe

Firmado por: Emma Carmelo Pascual Fecha: 21/02/2019 14:10:51
UNIVERSIDAD DE LA LAGUNA

4) Pacientes que no están en diálisis o con catéteres intravasculares.
5) No ADVP conocidos.
6) No viajar en los últimos 3 meses fuera de España.
7) Se incluyen a pacientes pediátricos.

Criterios de ingreso hospitalario:

1) Imposibilidad de manejo ambulatorio por falta de soporte social adecuado
2) Intolerancia digestiva
3) Enfermedades subyacentes crónicas de difícil control en presencia de fiebre prolongada
4) Afectación grave del estado general
5) Existencia de alguno de los siguientes criterios de gravedad:
    a. Alteración aguda de funciones superiores
    b. Presión arterial sistólica < 90 mm Hg o reducción > 40 mm Hg respecto a la presión arterial basal
    c. Signos de hipoperfusión periférica

Seguimiento en Atención primaria:

1) Reclutamiento de casos e inclusión en el estudio.
2) Consentimiento informado y petición analítica según protocolo.
3) Anamnesis dirigida según protocolo.
4) Nueva valoración clínica en la 1ª semana.
5) Derivar a CCEE de Medicina Interna si a pesar de todos los estudios realizados persiste sin diagnóstico o sin mejoría clínica.

Este documento incorpora firma electrónica, y es copia auténtica de un documento electrónico archivado por la ULL según la Ley 39/2015.
La autenticidad de este documento puede ser comprobada en la dirección: https://sede.ull.es/validacion/

Identificador del documento: 1764975 Código de verificación: 37vTZjpe

Firmado por: Emma Carmelo Pascual Fecha: 21/02/2019 14:10:51
UNIVERSIDAD DE LA LAGUNA

6) Derivar a Servicio de Urgencias Hospitalario los casos graves que cumplan criterio de ingreso.

8) Zoonosis emergentes como potenciales causas de Fiebre de Duración Intermedia y sus características principales:

A continuación se muestra la taxonomía clásica del orden *Rickettsiales* en la cual, inicialmente se incluía a *Coxiella* y *Rickettsia* dentro de la misma familia por sus características fenotípicas (Fig1). Posteriormente, la secuenciación de RNAm y del genoma completo de *Coxiella burnetti* permitió reclasificar al microorganismo dentro de la familia *Legionalleacee* en la cual se mantiene en la actualidad (Fig. 2). *Anaplasma* y *Ehrlichia*, sin embargo, en la taxonomía clásica eran clasificados en distintas familias del orden *Rickettsiales* pero en la taxonomía actual son consideradas distintos géneros de la misma familia Anaplasmataceae que, taxonómicamente siguen perteneciendo al orden Rickettsiales (alfa 1 Proteobacteria)[36].
Por otro lado, en la tabla 1, se muestran las características principales de las zoonosis más frecuentes conocidas como causa de FDI y, además, otras zoonosis emergentes según distribución espacial y vectores principales conocidos.[35]

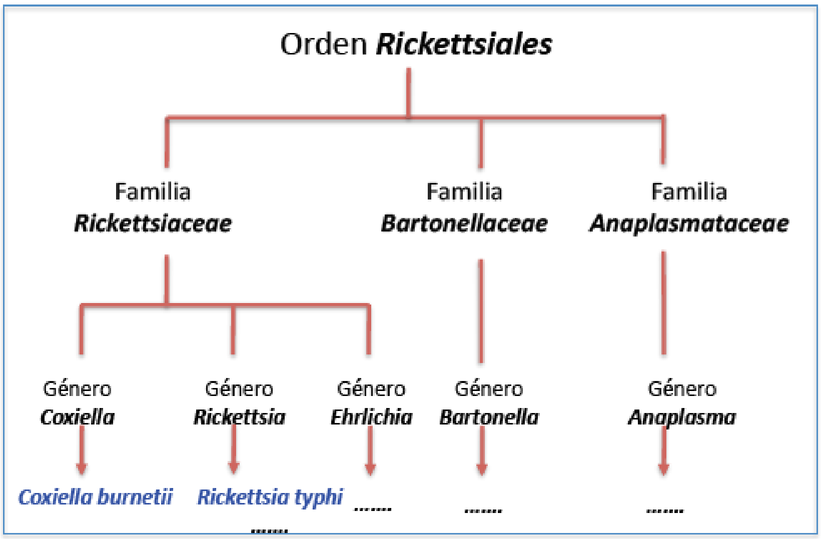

Fig 1. Taxonomía clásica de Coxiella burnetti y Rickettsia Tiphy

Este documento incorpora firma electrónica, y es copia auténtica de un documento electrónico archivado por la ULL según la Ley 39/2015.
La autenticidad de este documento puede ser comprobada en la dirección: https://sede.ull.es/validacion/

Identificador del documento: 1764975 Código de verificación: 37vTZjpe

Firmado por: Emma Carmelo Pascual Fecha: 21/02/2019 14:10:51
UNIVERSIDAD DE LA LAGUNA

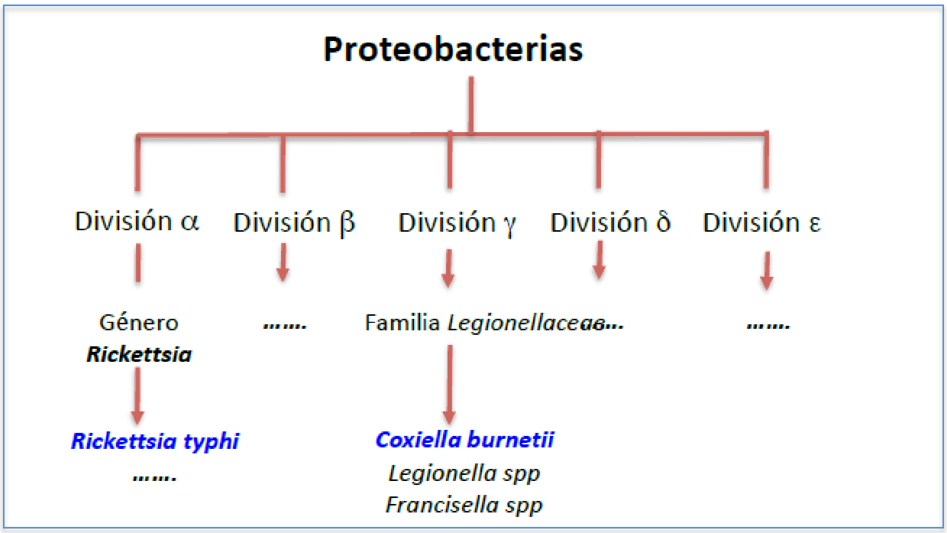

Fig 2. Taxonomía actual de *Rickettsi typhi* y *Coxiella burnetii*

Tabla 1. Zoonosis más frecuentes como causa de FDI y otras zoonosis emergentes.

ENFERMEDAD	ESPECIE	VECTOR	DISTRIBUCIÓN
Fiebre Q	*C. burnetii*	Reservorio: ganado, roedores. No hay vector.	Mundial
Tifus Murino	*R. typhi*	Pulgas (*Xenopsylla cheopis* and *Ctenocephalides felis*)	Mundial (más prevalente en zonas tropicales y subtropicales)
Fiebre manchada de pulgas	*R. felis*	Pulgas de gato (*Ctenocephalides felis*)	Mundial
FBM[1]	*R. conorii conorii*, *R. conorii israelensis*, *R. conorii caspia*, *R. conorii indica*	*Rhipicephalus* spp. garrapatas	Área mediterránea, Europa central, Rusia, India y África (no descritos casos clínicos en Canarias)
FBM-like	*R. monacensis*	*Ixodes ricinus* garrapatas	Europa
FBM-like	*R. massiliae*	*Rhipicephalus sanguineus* garrapatas	Área mediterránea, Argentina, USA?
FBM-like	*R. aeschlimannii*	*Hyalomma marginatum* garrapatas	Africa, Europa?
DEBONEL / TIBOLA[2]	*R. slovaca* *R. rioja* *R. raoultii*	*Dermacentor marginatus* garrapatas	Europa
LAR[3]	*R. sibirica mongolitimonae*	*Hyalomma* spp. y *Rhipicephalus pusillus* garrapatas	Europa, Africa.

Este documento incorpora firma electrónica, y es copia auténtica de un documento electrónico archivado por la ULL según la Ley 39/2015.
La autenticidad de este documento puede ser comprobada en la dirección: https://sede.ull.es/validacion/

Identificador del documento: 1764975 Código de verificación: 37vTZjpe

Firmado por: Emma Carmelo Pascual Fecha: 21/02/2019 14:10:51
UNIVERSIDAD DE LA LAGUNA

ATBF4	*R. africae*	*Amblyomma* spp. garrapata	Africa Sub-Sahariana e India
R. helvetica infection	*R. helvetica*	*Ixodes ricinus* garrapatas	Norte y Centro Europa, Asia
Ehrlichiosis granulocítica	*E. ewingii*	*A. americanum*	Norteamérica (en perros y humanos inmunodeprimidos)
Ehrlichiosis monocítica canina	*E. canis*	Garrapata marrón del perro (*Riphicephalus sanguineus)*	Mundial
Ehrlichiosis humana monocítica	*E. chaffeensis*	*Amblyomma americanum* *Dermacentor variabili*	Norteamérica Centroamérica
Anaplasmosis humana granulocítica	*A. phagocytophilum*	*Ixodes ricinus*	Europa Norteamérica Norte de África
Bartonellosis	*B. bacilliformis*	*Hombre*	Sur América (Perú, Ecuador, Colombia)
Enf arañazo de gato	*B. henselae*	*Pulgas gato doméstico*	Mundial
Fiebre de las Trincheras	*B. quintana*	*Piojos hombre (pediculus corporis)*	Mundial

1MSF: Fiebre Botonosa Mediterránea; 2DEBONEL/TIBOLA: *Dermacentor*-borne, necrosis, erythema, lymphadenopathy/Tick-borne lymphadenopathy; 3LAR: Lymphangitis-associated rickettsiosis; 4ATBF: African tick-bite fever.

Este documento incorpora firma electrónica, y es copia auténtica de un documento electrónico archivado por la ULL según la Ley 39/2015.
La autenticidad de este documento puede ser comprobada en la dirección: https://sede.ull.es/validacion/

Identificador del documento: 1764975 Código de verificación: 37vTZjpe

Firmado por: Emma Carmelo Pascual Fecha: 21/02/2019 14:10:51
UNIVERSIDAD DE LA LAGUNA

Algoritmo de diagnóstico-tratamiento:

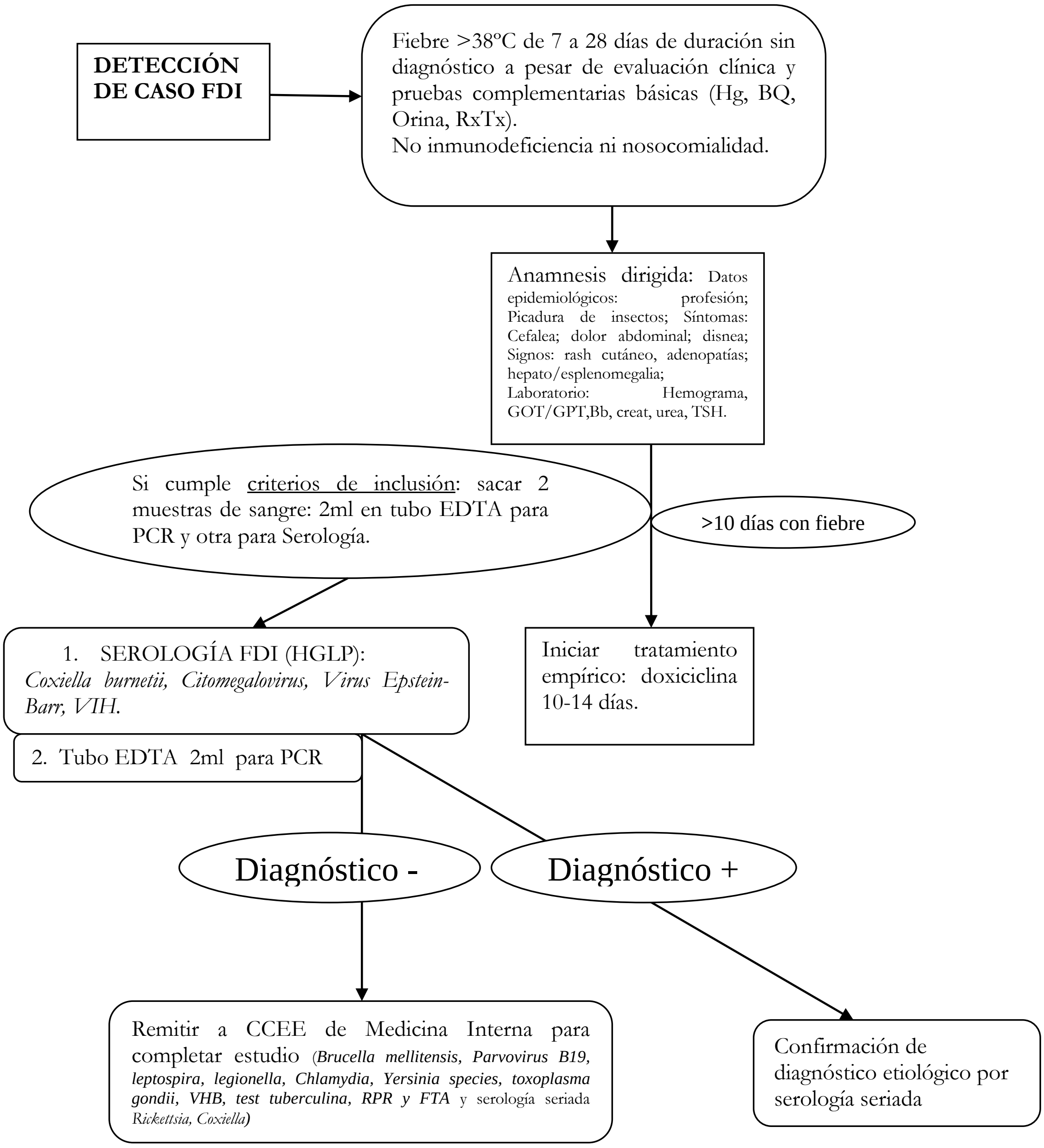

Este documento incorpora firma electrónica, y es copia auténtica de un documento electrónico archivado por la ULL según la Ley 39/2015.
La autenticidad de este documento puede ser comprobada en la dirección: https://sede.ull.es/validacion/

Identificador del documento: 1764975 Código de verificación: 37vTZjpe

Firmado por: Emma Carmelo Pascual Fecha: 21/02/2019 14:10:51
UNIVERSIDAD DE LA LAGUNA

Algoritmo proceso reclutamiento:

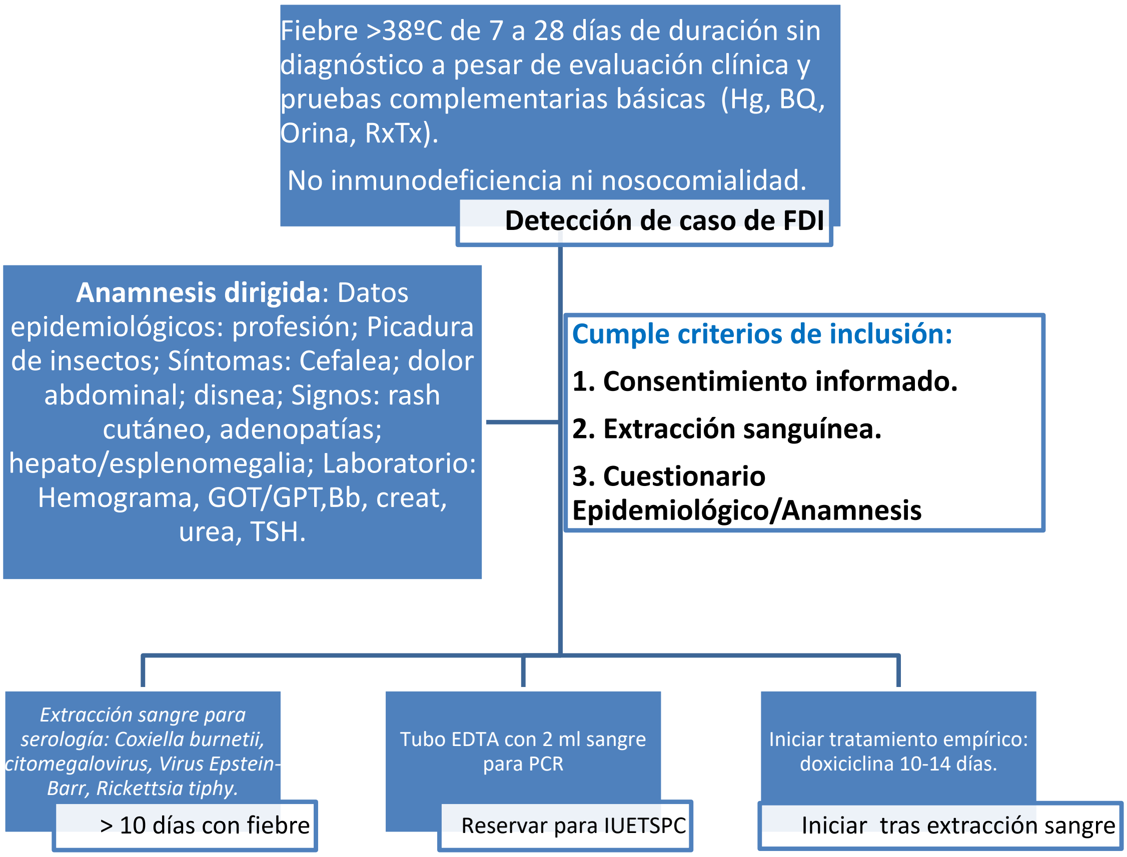

Este documento incorpora firma electrónica, y es copia auténtica de un documento electrónico archivado por la ULL según la Ley 39/2015.
La autenticidad de este documento puede ser comprobada en la dirección: https://sede.ull.es/validacion/

Identificador del documento: 1764975 Código de verificación: 37vTZjpe

Firmado por: Emma Carmelo Pascual Fecha: 21/02/2019 14:10:51
UNIVERSIDAD DE LA LAGUNA

Variables a estudio:

a. DATOS DEMOGRÁFICOS:
   i. Edad
   ii. Género
   iii. Localidad
b. DATOS EPIDEMIOLÓGICOS:
   i. Profesión.
   ii. Antecedente de picaduras o contacto con animales.
c. SÍNTOMAS CLÍNICOS
   i. Cefalea intensa, no intensa, no cefalea
   ii. Dolor abdominal (si/no)
   iii. Disnea (sí/no)
d. SIGNOS CLÍNICOS
   i. Picaduras, escara.
   ii. Rash cutáneo.
   iii. Adenopatías.
   iv. Hepato/esplenomegalia.
e. DATOS DE LABORATORIO
   i. Hemograma (leucocitos, plaquetas, hb)
   ii. Hipertransaminemia (GOT/GPT, GGT)
   iii. Colestasis (Bilirrubina total – directa- indirecta- Fosfatasa alcalina).
   iv. Función renal (Creatinina, urea).
   v. Función tiroidea (TSH).
   vi. Reactantes fase aguda: PCR, VSG, fibrinógeno, dímero-D.
f. ETIOLOGÍA
   i. ***Coxiella burnetii, Rickettsia typhi, Rickettsia conorii,*** *Citomegalovirus, virus Epstein-Barr, VIH*.
   ii. *Brucella mellitensis, Parvovirus B19, leptospira, legionella, Chlamydia, Yersinia species, toxoplasma gondii, VHB, test tuberculina, RPR y FTA, Borrelia Burgdorferi*.
   iii. ***Rickettsia felis, Rickettsia massiliae, Rickettsia africae, Rickettsia monacensis**, **Bartonella spp, Ehrlichia spp, Anaplasma spp.***
   iv. ***Congruencia entre resultado PCR y serología***.
g. Criterio de HOSPITALIZACIÓN (sí o no)
h. COMPLICACIONES (Diagnósticos al alta)
   i. Afectación respiratoria (neumonía/neumonitis/derrame pleural).

Este documento incorpora firma electrónica, y es copia auténtica de un documento electrónico archivado por la ULL según la Ley 39/2015.
La autenticidad de este documento puede ser comprobada en la dirección: https://sede.ull.es/validacion/

Identificador del documento: 1764975 Código de verificación: 37vTZjpe

Firmado por: Emma Carmelo Pascual Fecha: 21/02/2019 14:10:51
UNIVERSIDAD DE LA LAGUNA

ii. Afectación cardiaca (insuficiencia cardiaca/miocarditis).
iii. Afectación neurológica (meningitis…)
iv. Insuficiencia renal.
v. Afectación tiroidea (hiper/hipotiroidismo).
vi. Afectación ocular (uveítis anterior).
vii. Afectación cutánea (vasculitis).
viii. Otros.

Instrumentos usados en el estudio:

Instrumentos para recoger las muestras

Extracción de sangre: tubo para serología + tubo con EDTA para PCR

Consentimiento informado.

Petición de laboratorio.

Procesamiento de las muestras

Técnicas serológicas empleadas: Las técnicas serológicas utilizadas y los puntos de corte son: IgM ≥1/64 o, IgG≥1/1024 mediante inmunofluorescencia indirecta (Virion, Richland, USA en 1983-1989 y Vitaltech en 2004-2005) para *Coxiella burnetti*; IgM≥1/80 mediante inmunofluorescencia indirecta (Pasteur Laboratories, Marnes, la Coquete, Francia desde 1986 y bioMérieux desde 1987) para *Rickettsia typhi* y *Rickettsia conorii*; técnica de aglutinación con Rosa Bengala (bioMérieux, Lyon, Francia) para *Brucella melitensis*; IgM positiva mediante IQL (inmunoquimioluminiscencia) para CMV y IgM positivas mediante método ELISA para VEB. Los puntos de corte utilizados son compatibles con infección aguda o reciente.

Técnicas de PCR empleadas: 200 μl de cada muestra de sangre congelada serán sometidos a purificacion de ADN total mediante Kit comerciales (QiampDNA mini kit de Qiagen o similar). El diagnóstico molecular se realizará mediante PCR según protocolos establecidos y secuenciación de los fragmentos amplificados[37-40]. Las secuencias serán alineadas mediante MEGA6 y BLAST (NCBI).

El remanente de las alícuotas será destruido por el Sistema de gestión de residuos del IUETSPC una vez hayan sido procesadas.

Este documento incorpora firma electrónica, y es copia auténtica de un documento electrónico archivado por la ULL según la Ley 39/2015.
La autenticidad de este documento puede ser comprobada en la dirección: https://sede.ull.es/validacion/

Identificador del documento: 1764975 Código de verificación: 37vTZjpe

Firmado por: Emma Carmelo Pascual Fecha: 21/02/2019 14:10:51
UNIVERSIDAD DE LA LAGUNA

Análisis estadístico:

Las variables cuantitativas se describirán mediante los estadísticos: media, mediana, desviación estándar, máximo y mínimo. Las variables cualitativas se describirán mediante las frecuencias absolutas, relativas y porcentajes. Las asociaciones entre variables se evaluarán mediante pruebas de Chi-Cuadrado, t de Student o coeficiente r de Pearson.

Las hipótesis 2,3 y 4 se evaluarán usando modelos lineales generales que incluyan las variables de confusión requeridas en cada objetivo. Las estimaciones se harán con un intervalo de confianza del 95%. Los contrastes de hipótesis se evaluarán con un nivel de confianza del 5%. Los análisis estadísticos se realizarán con los paquetes SPSS versión 21 y R versión 3.0.2

Limitaciones de estudio:

El reclutamiento de los casos de FDI dependerá del grupo de trabajo de Atención Primaria y Servicio de Urgencias, posibles irregularidades en el sistema podrían alterar la total inclusión de nuevos casos FDI. Los pacientes que pudiesen ser atendidos en servicios sanitarios privados no serán incluidos en el estudio. Por todo ello existe el riesgo de que las incidencias se subestimen.

Convenio de colaboración:

Se ha realizado un convenio de colaboración entre el Área de Salud de la Palma y de El Hierro, el Instituto Universitario de Enfermedades Tropicales y Salud Pública de Canarias (IUETSPC) de la Universidad de La Laguna y la Universidad de Barcelona en el que se establece la interrelación de cada una de las partes en el proyecto**.** En el momento actual dicho convenio está en manos de la administración jurídica, en proceso de evaluación y solicitud de firmas.

3. ASPECTOS ÉTICOS

El presente proyecto debe recibir aprobación por parte del Comité de Ética del Hospital Universitario de Canarias antes de comenzar su desarrollo.

Las muestras bilógicas se destruirán inmediatamente después de realizar las PCR de diagnóstico.

Este documento incorpora firma electrónica, y es copia auténtica de un documento electrónico archivado por la ULL según la Ley 39/2015.
La autenticidad de este documento puede ser comprobada en la dirección: https://sede.ull.es/validacion/

Identificador del documento: 1764975 Código de verificación: 37vTZjpe

Firmado por: Emma Carmelo Pascual Fecha: 21/02/2019 14:10:51
UNIVERSIDAD DE LA LAGUNA

El estudio se realizará en conformidad con los principios de la Declaración de Helsinki adoptada por la 18ª Asamblea Médica Mundial, Helsinki, Finlandia en 1964 y enmendada en Tokio (1975), Venecia (1983), Hong Kong (1989), Sudáfrica (1996), Edimburgo (2000), Washington (2002), Tokio (2004), Seúl (2008), Brasil (2013); y las Leyes y Reglamentos vigentes en Europa y España.

El paciente debe otorgar su consentimiento antes de ser admitido en el estudio clínico. El médico habrá de explicar la naturaleza, propósitos y posibles consecuencias del estudio, de una manera comprensible al paciente. La información proporcionada por el médico deberá ser también registrada.
El sujeto del estudio otorgará su consentimiento, firmando el modelo correspondiente que también deberá llevar la firma del investigador.
El investigador no iniciará ninguna investigación correspondiente al estudio hasta que haya obtenido el consentimiento del paciente.

Con el fin de garantizar la confidencialidad de los datos de los pacientes participantes en el estudio, sólo tendrán acceso a los mismos el investigador y su equipo de colaboradores. Los datos serán codificados para asegurar el anonimato.

El tratamiento, la comunicación y la cesión de los datos de carácter personal de todos los sujetos participantes se ajustará a lo dispuesto en la Ley Orgánica 3/2018, de 5 de diciembre de Protección de Datos Personales y garantía de los derechos digitales, y a la aplicación de del Reglamento (UE) 2016/679 del Parlamento europeo y del Consejo de 27 de abril de 2016 de Protección de Datos (RGPD).

4. RESULTADOS ESPERADOS

Este estudio tendrá el siguiente impacto positivo en la población de la isla de La Palma, El Hierro y la Comunidad Autónoma de Canarias:

- Conocimiento de tasas de las distintas etiologías de FDI, temporalidad, género/edad, distribución espacial por CAP/municipios.
- Extrapolar un perfil etiológico de FDI en las islas Canarias y conocer sus características diferenciales respecto al perfil etiológico de FDI en la península.

Este documento incorpora firma electrónica, y es copia auténtica de un documento electrónico archivado por la ULL según la Ley 39/2015.
La autenticidad de este documento puede ser comprobada en la dirección: https://sede.ull.es/validacion/

Identificador del documento: 1764975 Código de verificación: 37vTZjpe

Firmado por: Emma Carmelo Pascual Fecha: 21/02/2019 14:10:51
UNIVERSIDAD DE LA LAGUNA

- Se incrementará el número de diagnósticos positivos de FDI gracias a la aplicación de un protocolo dirigido y al trabajo en equipo entre los distintos profesionales sanitarios de las islas y las distintas instituciones implicadas. Implementar un protocolo de FDI adecuado a las características diferenciales de las islas Canarias.
- Mejorar la prevención de nuevos casos gracias al mejor conocimiento de su etiología permitiendo evaluar la necesidad de estrategias de salud pública encaminadas a controlar los vectores transmisores implicados en dichas zoonosis.

Impacto positivo a nivel nacional e internacional:

- Primer estudio de incidencias de FDI a nivel nacional. Probablemente las causas más frecuentes de FDI sean las mismas que las detectadas en otros estudios a nivel nacional, la fiebre Q y el tifus murino, sin embargo, hasta ahora en la literatura científica sólo se han aportado estudios de prevalencia.
- Este estudio pretende apoyar toda la evidencia científica española sobre FDI con la intención de hegemonizar el uso de dicho término a nivel internacional, tanto desde el punto de vista conceptual como su uso en la práctica médica diaria.
- El mayor conocimiento de las diferencias en el perfil clínico y analítico de las distintas causas de FDI nos permitirá precisar la sospecha diagnóstica y anticipar nuestra actitud terapéutica antes de conocer los resultados serológicos definitivos.
- El conocimiento de las patologías con mayor riesgo de hospitalización y complicaciones nos permitirá establecer protocolos de actuación para mejorar su tratamiento y prevención y establecer factores de riesgo predictores de hospitalización y complicaciones.
- Se ampliará el número de diagnósticos nuevos implementando métodos de diagnóstico molecular que incluirán etiologías no consideradas hasta el momento y, como consecuencia, mejoraremos su atención y tratamiento.

Este documento incorpora firma electrónica, y es copia auténtica de un documento electrónico archivado por la ULL según la Ley 39/2015.
La autenticidad de este documento puede ser comprobada en la dirección: https://sede.ull.es/validacion/

Identificador del documento: 1764975 Código de verificación: 37vTZjpe

Firmado por: Emma Carmelo Pascual Fecha: 21/02/2019 14:10:51
UNIVERSIDAD DE LA LAGUNA

5. BIBLIOGRAFIA

1. Bernabeu-Wittel M, Cordero E, Viciana P, Pachón J. **Etiología y criterios de ingreso del síndrome febril sin focalidad.** Med Clin (Barc). 1999;113:718–9.
2. Bernabeu-Wittel M, Pachón J, Alarcón A, López-Cortés LF, Viciana P, Jiménez-Mejías ME, Villanueva JL, Torronteras R, Caballero-Granado FJ. **Murine typhus as a common cause of fever of intermediate duration: a 17-year study in the south of Spain.** Arch Intern Med. 1999 Apr 26;159(8):872-6.
3. Espinosa N, Cañas E, Bernabeu-Wittel M, Martín A, Viciana P, Pachón J**. The changing etiology of fever of intermediate duration.**.Enferm Infecc Microbiol Clin. 2010 Aug-Sep;28(7):416-20. doi: 10.1016/j.eimc.2009.07.014. Epub 2010 Feb 12.
4. Tudela Hita P, Urrutia de Diego A. **Fiebre aguda y fiebre de origen desconocido.** XVI edición. En: Farreras-Rozman Medicina Interna. Elsevier España SL, 2010; 321:2574–80.
5. Mackowiak PA, Durack DT. In: Mandell, Douglas, and Bennett's, Principles and **Practice of Infectious Diseases. Mandell GL, Bennett JE, Dolin R, eds. 7th. Edition.** Philadelphia: Elsevier; 2010. 51:779-89.
6. José A. Oteo. **Fever of intermediate duration: New times, new tools and change of spectrum**. Enferm Infecc Microbiol Clin. 2010;28(7):407–408.
7. David H. Walker. **.** Chapter 38: **Rickettsiae**. 4th edition. Baron S, editor. Medical Microbiology. Galveston (TX): University of Texas medical Branch at Galeston; 1996.
8. Aránzazu Portillo and José A. Oteo. **Rickettsiosis as Threat for Traveller.** Cap 1. Current Topics in Tropical Medicina. Edited by Dr. Alfonso Rodríguez-Morales. 2012. www.intechopen.com
9. European Journal of Epidemiology 2003 March:18(3): 259-262. **Seroprevalence of infection by Coxiella burnetii in Canary Islands (Spain).** Bolaños M, Santana OE, Ángel-Moreno A, Pérez-Arellano JL, Limiñana JM, Serra-Majem L, Martín-Sánchez AM.
10. Rev Clin Esp. 1997 Jan;197(1):69. **A review of Q fever in the Canary Islands.** Pascual Velasco F.
11. Scand J Infect Dis. 1996;28(5):533-4. **Clinical presentation of acute Q fever in lanzarote (Canary Islands): a 2-year prospective study.** Pascual Velasco F(1), Borobio Enciso MV,

Este documento incorpora firma electrónica, y es copia auténtica de un documento electrónico archivado por la ULL según la Ley 39/2015.
La autenticidad de este documento puede ser comprobada en la dirección: https://sede.ull.es/validacion/

Identificador del documento: 1764975 Código de verificación: 37vTZjpe

Firmado por: Emma Carmelo Pascual Fecha: 21/02/2019 14:10:51
UNIVERSIDAD DE LA LAGUNA

González Lama Z, Carrascosa Porras M. (1)Service of Internal Medicine, Hospital of Laredo, Cantabria, Spain.

12. Enferm Infecc Microbiol Clin. 2003 Jan;21(1):20-3. **Q fever in Gran Canaria: 40 new cases.** Bolaños M(1), Santana OE, Pérez-Arellano JL, Angel-Moreno A, Moreno G, Burgazzoli JL, Martín-Sánchez AM.
13. An Med Interna. 1989 Oct;6(10):527-30. **Q fever on the island of La Palma. A review of 35 patients.** Millán Mon A, Argany Fajardo A, Febles Bethencourt J, González Caloca C, Vento Remedios TE, Fernández Cabrera M.
14. An Med Interna 1991 May; 8P85r9:233-4. **Prevalence of antibodies against Coxiella burnetii in Healthy population in Lanzarote (Canary Islands).** Pascual Veleasco F, Otero Ferrio I, Borobio Enciso MV.
15. An Med Inerna. 1992 Sep;9(9):428-32. **Seroprevalence of Q fever among th adult population of Lanzarote (Canary Islands).** Pascual Velasco F, Rodríguez Pérez JC, Otero Ferrio I, Borobio Enciso MV.
16. Bolaños-Rivero M, Santana-Rodríguez E, Ángel-Moreno A, Hernández-Cabrera M, Limiñana-Canal JM, Carranza-Rodríguez C, Martín-Sánchez AM, Pérez-Arellano JL. **Seroprevalence of Rickettsi typhi and Rickettsi conorii infections in the Canary Islands (Spain).** International Journal of Infectiuous Diseases. 2011; 15:481-485.
17. Miguélez M, Laynez P, Linares M, Hayek M, Abella L, Marañez I. Murine typhus in Tenerife. **Clinicoepidemiological study and differential clinical features with Q fever.** Med Clin (Barc) 2003; 121:613-5.
18. Hernández-Cabrera M, Angel-Moreno A, Santan E, Bolaños M, Frncés A, Martín-Sánchez MS, et al. **Murine typhus with renal involvement in Canary Islands, Spain.** Emerg Infect Dis 2004;10:740-3.
19. Enferm Infecc Microbiol Clin. 2012 Aug;30(7):427-8. doi: 10.1016/j.eimc.2012.02.008. Epub 2012 Apr 12. **Rickettsia typhi. A new causative agent of round pneumonia in adults.** Velasco-Tirado V, Hernández-Cabrera M, Pisos-Álamo E, Pérez-Arellano JL. Hospital Insular de Gran Canaria, Spain.

Este documento incorpora firma electrónica, y es copia auténtica de un documento electrónico archivado por la ULL según la Ley 39/2015.
La autenticidad de este documento puede ser comprobada en la dirección: https://sede.ull.es/validacion/

Identificador del documento: 1764975 Código de verificación: 37vTZjpe

Firmado por: Emma Carmelo Pascual *UNIVERSIDAD DE LA LAGUNA* Fecha: 21/02/2019 14:10:51

20. Enferm Infecc Microbiol Clin. 2011:29(3):232-242. **Uveitis anterior bilateral e infección por Rickettsia typhi.** Beltrán LM, García S, Vallejo AJ, Bernabeu-Wittel M. Int. J. Environ. Res. Public Health 2009, 6, 2526-2533;doi:10.3390/ijerph6102526.
21. Lancet 2006 Apr 1;367 (9516): 1116. **A souvenir from the Canary Islands.** Somasundaram R, Loddenkemper C, Zeitz M, Schneider T.
22. Internist (Berl.) 2007 Apr;48(4):413-9. **Fever of intermediate duration after return from the Canary Islands.** Basrai D, Pox C, Schmiegel W.
23. Emerging Infectious Diseases • www.cdc.gov/eid • Vol. 11, No. 12, December 2005. **Human Rickettsia felis Infection, Canary Islands, Spain.** Jose-Luis Pérez-Arellano, Florence Fenollar,Alfonso Angel-Moreno, Margarita Bolaños, Michele Hernández, Evora Santana, Marion Hemmersbach-Miller, Antonio-M Martín, and Didier Raoul
24. Oteo JA, Portillo A, Santibañez S, Blanco JR, Pérez L, Ibarra V. Human **Rickettsia felis infections diagnosed by PCR in Spain.** J Clin Microbiol. 2006;44:2669–71.
25. Eur J Clin Microbiol Infect Dis. 2006 Jun;25(6):375-81.**Seroepidemiological study of Rickettsia felis, Rickettsia typhi, and Rickettsia conorii infection among the population of southern Spain.** Bernabeu-Wittel M(1), del Toro MD, Nogueras MM, Muniain MA, Cardeñosa N, Márquez. FJ, Segura F, Pachón J.Department of Infectious Diseases, Hospitales Universitarios Virgen del Rocío. Spain.
26. Am. J. Trop. Med. Hyg. 74(1), 2006, pp. 123-126. **Short report: serological evidence of infection with Rickettsia typhi and Rickettsia felis among the human population of Catalonia, in the northeast of spain.** Nogueras M, Cardeñosa N, Sanfeliu I, Muñóz T, Font B, Segura F. Infectious Diseases Program. Department of Internal Medicine, Corporació Sanitaria Parc Taulí, Sabadell, Barcelona, Spain.
27. Oteo JA, Brouqui P. **Ehrlichiosis y anaplasmosis humana.** Enferm Infecc Microbiol Clin. 2005;23:375–80.
28. Jado I, Oteo JA, Aldamiz M, Gil H, Escudero R, Ibarra V, et al. **Rickettsia monacensis.** A new pathogen causing human disease. Emerg Infect Dis. 2007;13:1405–7.
29. Aguirrebengoa K, Portillo A, Santibañez S, Marín JJ, Montejo M, Oteo JA. **First human Rickettsia sibirica mongolitimonae infection in Spain**. Emerg Infect Dis. 2008;14:528–9.

Este documento incorpora firma electrónica, y es copia auténtica de un documento electrónico archivado por la ULL según la Ley 39/2015.
La autenticidad de este documento puede ser comprobada en la dirección: https://sede.ull.es/validacion/

Identificador del documento: 1764975 Código de verificación: 37vTZjpe

Firmado por: Emma Carmelo Pascual *UNIVERSIDAD DE LA LAGUNA* Fecha: 21/02/2019 14:10:51

30. Oteo JA, Ibarra V, Blanco JR, Martínez de Artola V, Márquez FJ, Portillo A, et al. ***Dermacentor*-borne necrosis erythema and lymphadenopathy: clinical and epidemiological features of a new tickborne disease.** Clin Microbiol Infect. 2004;10:327–31.
31. Clin Microbiol Infect. 2009 Dec;15 Suppl 2:6-7. doi: 10.1111/j.1469-0691.2008.02729.x. Epub 2009 Apr 3.**Anaplasma phagocytophilum is not an aetiological agent of fever of intermediate duration in Gran Canaria (Spain).** Bolaños M(1), Santana E, Carranza C, Anda P, Jado I, Hernández-Cabrera M, Martín-Sánchez AM, Pérez-Arellano JL.
32. Sánchez-Tejero E, García-Sánchez E. **Empirical treatment with doxycycline for fever of intermediate duration?.**Enferm Infecc Microbiol Clin. 2004 Jun-Jul;22(6):365; author reply 365-6.
33. Instituto Nacional de Estadística. Cifras de población oficial resultante del Padrón municipal en la Isla de la Palma [internet]: 2017. [consultado 09 Feb 2019]. Disponible en: https://www3.gobiernodecanarias.org/istac/statistical-visualizer/visualizer/data.html?resourceType=indicator&resourceId=POBLACION&measure=ABSOLUTE&geo=ES70#visualization/table
34. Wikipedia. The free Encyclopedia. Canary Islands [internet]:2001. [consultado 10 Ag 2018]. Disponible en: https://en.wikipedia.org/w/index.php?title=Canary_Islands&oldid=767377423
35. Aránzazu Portillo and José A. Oteo. **Rickettsiosis as Threat for Traveller.** Cap 1. Current Topics in Tropical Medicina. Edited by Dr. Alfonso Rodríguez-Morales. 2012. www.intechopen.com
36. Tesis Doctoral presentada por Dña. Margarita Bolaños Rivero. Dirigida por los Profesores Dr. Antonio Manuel Martín Sánchez, Dra. O. Évora Santana Rodríguez y Dr. José Luis Pérez Arellano. **Epidemiología, Agentes causales y Métodos diagnósticos en pacientes con Fiebre de Duración Intermedia en Gran Canaria**. 23 de Nov. 2015
37. Oteo el al. Journal of Clinical Microbiology, July 2006, p. 2669–2671
38. Jado et al. Journal of Clinical Microbiology, Dec. 2006, p. 4572–4576
39. Yanes et al., Microb Ecol (2018) 75:264–27

Este documento incorpora firma electrónica, y es copia auténtica de un documento electrónico archivado por la ULL según la Ley 39/2015.
La autenticidad de este documento puede ser comprobada en la dirección: https://sede.ull.es/validacion/

Identificador del documento: 1764975 Código de verificación: 37vTZjpe

Firmado por: Emma Carmelo Pascual Fecha: 21/02/2019 14:10:51
UNIVERSIDAD DE LA LAGUNA

40. M. Berri, K. Laroucau, A. Rodolakis. **The detection of *Coxiella burnetii* from ovine genital swabs, milk and fecal samples by the use of a single touchdown polymerase chain reaction**. Vet Microbiol., 72 (2000), pp. 285-329

Este documento incorpora firma electrónica, y es copia auténtica de un documento electrónico archivado por la ULL según la Ley 39/2015.
La autenticidad de este documento puede ser comprobada en la dirección: https://sede.ull.es/validacion/

Identificador del documento: 1764975 Código de verificación: 37vTZjpe

Firmado por: Emma Carmelo Pascual Fecha: 21/02/2019 14:10:51
UNIVERSIDAD DE LA LAGUNA
